# Dual‐Mode Thio‐MacMillan Organocatalysts: Stereoselective Diels–Alder Reactions or Sacrificial Self‐Cyclization to *N*‐Bridged Bicyclic Lactams

**DOI:** 10.1002/chem.202503017

**Published:** 2025-12-18

**Authors:** Marian S. R. Ebeling, Luca V. Parziale, Marc Sachsenhauser, Christoph J. B. Seifert, Nathalie J. Kurrle, Oliver Trapp

**Affiliations:** ^1^ Department of Chemistry Ludwig‐Maximilians‐University Munich Munich Germany; ^2^ Max‐Planck‐Institute for Astronomy Heidelberg Germany

**Keywords:** enantioselectivity, fused‐ring systems, nitrogen heterocycles, organocatalysis, thiolactams

## Abstract

Chiral secondary amines in lactams are commonly used as chiral organocatalysts (MacMillan catalysts) in a broad range of transformations. The related thiolactam derivatives are less explored although possessing some significantly differing properties originating from the stereoelectronic characteristics of sulfur. In the present study, we present a streamlined synthesis and application of imidazolidine‐4‐thiones from naturally abundant L‐phenylalanine. Suitable substituents enable asymmetric Diels–Alder reactions with high enantioselectivity and yield. Here, we discovered the formation of a novel bicyclic thiolactam aldehyde motif in absence of the diene. The obtained hetero‐bicycle, with four consecutive stereocenters, is formed in high yield and stereoselectivity from imidazolidine‐4‐thione and two cinnamaldehyde units, whereas the first gen. MacMillan catalyst shows no reactivity. We propose a mechanism of formation which is supported by DFT calculations, revealing a combination of thermodynamic and kinetic factors for the observed selectivity. Our results demonstrate the surprising versatility of imidazolidine‐4‐thiones, as this compound class can not only engage as a catalyst but can simultaneously participate as a reagent to form complex structures. The hetero‐bicyclic skeleton is accessible in a single step and allows for facile structural modifications.

## Graphical abstract

The study of the Diels–Alder reaction of cinnamaldehyde and cyclopentadiene, catalyzed by chiral imidazolidine‐4‐thiones, led to the discovery of a highly regio‐ and stereoselective Michael‐addition and consecutive ring closure. Here, the imidazolidine‐4‐thione not only catalytically activates cinnamaldehyde but also participates as the nucleophile to form a complex *N*‐bridged bicyclic system, as elucidated by DFT calculations.

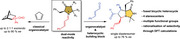

## 1 Introduction

**FIGURE 1:**
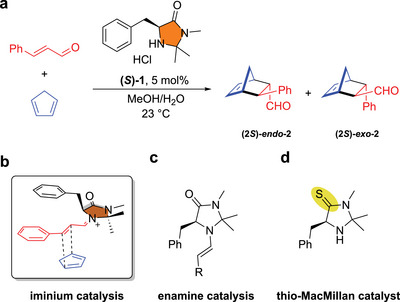
First generation MacMillan catalyst **(*S*)‐1** enables various reaction modes. (a) Asymmetric Diels–Alder reaction of *trans*‐cinnamaldehyde and cyclopentadiene to **2,** catalyzed by imidazolidin‐4‐one catalyst. (b) Catalytically active iminium ion intermediate in the cycloaddition transition state. (c) Catalytically active enamine species. (d) thio‐MacMillan catalyst.

The groundbreaking contributions to enantioselective Diels–Alder and aldol reactions have established asymmetric organocatalysis [1, 2, 3, 4] conceptually and in its broad synthetic applications as a cornerstone of modern asymmetric catalysis. List's L‐proline‐ and MacMillan's amino acid‐derived imidazolidin‐4‐one organocatalysts, proved to be a universal platform for many different applications. Particularly MacMillan's chiral imidazolin‐4‐ones enabled a variety of asymmetric transformations such as α‐alkylation,[5, 6] and ‐halogenation [7, 8] of aldehydes, as well as 1,3‐dipolar cycloadditions,[9] hydride reductions,[10] Friedel–Crafts alkylations,[11] aldol reactions [12], or 1,4‐addition to α,β‐unsaturated aldehydes [13] based on enamine and iminium ion catalysis. These reactions are either based on LUMO activation by formation of an electron deficient iminium‐ion, or on enamine formation with an energetic increase of the HOMO orbital (Figure 1b,c).

As imidazolidin‐4‐ones, such as **1**, were utilized in various organocatalytic reactions, Su et al. investigated imidazolidine‐4‐thiones (**Im4t**) for their catalytical efficacy, namely the thionated MacMillan catalyst based on **(*S*)‐1** (Figure 1d) [14]. In the thiolactam, the imine thiol tautomer is more favored (compared to lactams), resulting in a more rigid backbone [15]. These systems catalyzed asymmetric α‐oxyaminations, Friedel–Crafts alkylations and Diels–Alder reactions of aldehydes with high selectivity and conversion, however no substantial improvement over the MacMillan system was found [14, 16, 17, 18].

Independently, our group found imidazolidine‐4‐thiones (*N* ^3^ as a secondary thiolactam) to be prebiotically plausible organocatalysts that may have emerged on the early Earth and applied these in the α‐alkylation of aldehydes under prebiotic conditions.[19, 20, 21, 22] In this work, we broaden the structural variety of the catalysts while simplifying their synthesis. Additionally, the application of these structures in the organocatalyzed Diels–Alder reaction was envisioned to investigate their efficacy and the influence of the *N* ^3^‐methylation on the catalytic activity.

## 2 Results and Discussion

To access imidazolidine‐4‐thione catalysts **3a–3c** in a more efficient and modular approach compared to the previously described prebiotic route,[19] we developed a straightforward synthesis, starting from the amino acid phenylalanine, to access various chiral catalysts with excellent enantiomeric excess. First, Boc‐proctected phenylalanine **4** was activated by formation of a mixed anhydride. Ammonolysis with (methyl‐)ammonium chloride yields the corresponding (*N*‐methyl‐)amide **5** [23]. Subsequent thionation using Lawesson's reagent gives thioamide **6**[24] which is isolated as the hydrochloride salt **7** after acidic Boc‐deprotection. This salt is air stable and was used for the synthesis of imidazolidine‐4‐thiones **3** through condensation with various aldehydes or ketones. In case of the nonalkylated thiolactam **(*S*)‐3b**, an *S*‐alkylation with MeI or BrCH_2_CN to the catalysts **8** or **9** was also conducted. Notably, these *S*‐alkylated products should exhibit a planar ring structure by enforcing the endocyclic double bond character.

**FIGURE 2:**
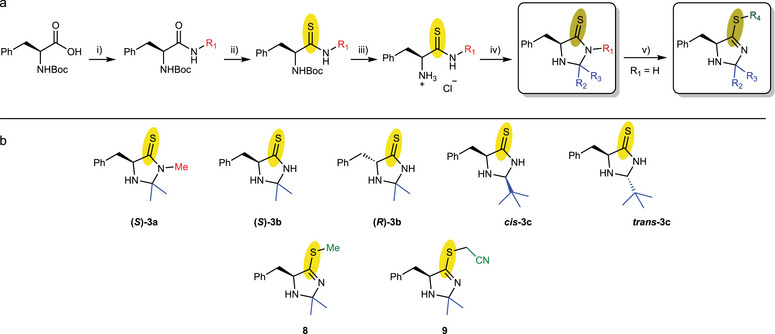
General synthetic pathway to (substituted) imidazolidine‐4‐thiones. (a) Synthesis sequence starting from D‐ or L‐Boc‐Phe‐OH: (i) ethyl chloroformate, NEt_3_ in THF, then ammonium chloride (for R_1_ = H) and methyl‐ammonium chloride (for R_1_ = Me) (ii) Lawesson's reagent in THF (iii) HCl (4 M) in dioxane (iv) carbonyl and *p*‐TsOH in MeOH v) alkylhalide and DBU in MeOH. (b) Synthesized catalyst library.

Previously, catalysts **3b–c** could be synthesized from the corresponding aldehydes or ketones, HCN, KOH, H_2_S and NH_3_ under prebiotic conditions in a Strecker‐type reaction [19], while **(*S*)**‐**3a** has been accessed by Su through thionation of the MacMillan catalyst **(*S*)**‐**1** [14]. In contrast, our modular synthesis enables straightforward diversification of the scope in the last, ring‐closing step (Figure 2a). To evaluate the capabilities of our catalyst library (Figure 2b) in comparison with published screenings, we applied it in the organocatalyzed Diels–Alder reaction of cyclopentadiene and *trans*‐cinnamaldehyde under reaction conditions adopted from the MacMillan group [4, 14].

When using the (5*S*)‐, or (5*R*)‐MacMillan catalyst **1** (Table 1, entries 1–2) under these conditions, we obtained comparable yields of up to 91% and *ee*’s of up to 94%. For example, with the thionated catalyst **(*S*)‐3a** (entry 3) a yield of 85% and an *ee* of up to 90% was obtained. For catalysts **(*R*/*S*)‐3b** without the *N* ^3^‐methyl group (R_1_ = H, entries 4–5), yields dropped to 75–77 % and the *ee* was in the range of 80%. The significant decrease in *ee* and yield for **(*S*)‐3b** and **(*R*)‐3b** can be explained by partial racemization of the catalyst and a remarkable side reaction described below. Under comparable reaction conditions, Su reported a yield of 96% and up to 95% *ee* for the catalyst **(*S*)‐3a**. These results show that the *N* ^3^‐methyl group is increasing the catalyst stability and thus improving yield and selectivity.

**TABLE 1:** Results of Diels–Alder reactions catalyzed by imidazolidine‐4‐thione or imidazolidin‐4‐one catalysts.

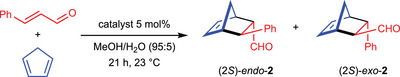					
Entry	Catalyst^a^	Yield^b^ [%]	*exo*:*endo* ^b^	(2*S*)*‐exo ee* [%]	(2*S*)*‐endo ee* [%]
1	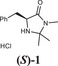	86.4	1.32: 1	92.0	93.9
2	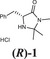	90.8	1.55: 1	93.2^c^	92.4^c^
3	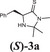	84.8	1.34: 1	88.3	90.3
4	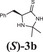	76.9	1.33: 1	81.8	82.8
5	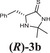	74.5	1.41: 1	78.1^c^	75.5^c^
6	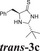	50.8	1.99: 1	55.9	6.0
7	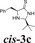	56.3	2.07: 1	56.1	8.5
8	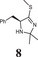	39.8	1.31: 1	77.6	76.9
9	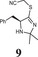	57.2	1.35: 1	79.1	81.2

^a^ Reaction time 21 h, catalyst loading 5 mol%, 1 M MeOH/H_2_O (95:5). Each reaction was performed thrice and the results were averaged, the standard deviation is given in the Supporting Information.

^b^ The *exo*:*endo* ratio and the *ee* were determined by GC (Figure S1, Table S2) while the yields were determined by referencing to an internal standard (Table S1, Figure S2).

^c^ The *ee* corresponds to the respective (2*R*) isomer as the major product.

The introduction of the bulky *t*‐Bu‐groups in *trans*‐**3c** and *cis*‐**3c** (entries 6–7) significantly decreased the yield to 51–56 %, presumably due to more pronounced steric shielding of the secondary amine. The absolute configuration of the *t*‐Bu‐group had no influence on the product (*exo ee* 56 %). However, the (2*S*)‐*exo*‐**2** product was favored significantly with a ratio of around 3.5:1 compared to the other three stereoisomers. When the *S*‐alkylated thiolactams **8** and **9** were applied (entries 8–9), the yields were 40% and 57%, respectively, with comparable *ee*’s to entries 4–5. Analysis of the reaction mixture revealed *in situ* decomposition of the catalysts by partial hydrolysis of **8** to the free thiolactam **3b**. This might explain the slightly diminished overall yield, and the maintained enantioselectivity.

**FIGURE 3:**
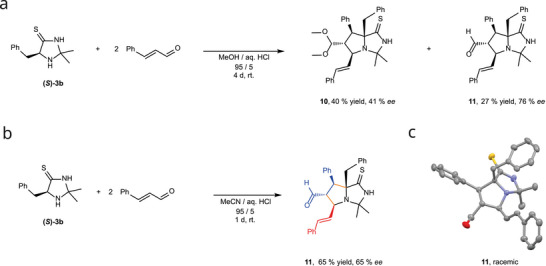
Elucidation of the addition of **(*S*)**‐**3b** and trans‐cinnamaldehyde. (a) Reaction of the thiolactam **(*S*)‐3b** and two equivalents of *trans*‐cinnamaldehyde to the bicyclic acetal **10** and aldehyde **11**. (b) Optimized reaction condition towards the aldehyde **11**. (c) Crystal structure of racemic bicycle **11**. Thermal ellipsoids are drawn at the 25 % probability level and hydrogen atoms were omitted for clarity.

To probe the mode of addition of the aldehyde to the catalyst, *trans*‐cinnamaldehyde was treated with stoichiometric amounts of imidazolidine‐4‐thione **(*S*)‐3b** in the absence of the diene. Analysis revealed the unexpected formation of two new major products. After isolation, we elucidated the structures (Figure 3a) by NMR and HRMS analysis and identified a bicyclic fused methanol acetal **10** and its unprotected aldehyde **11**. The structures were verified by X‐ray crystallography of **11** (Figures 3b and S3). While the single crystal diffraction showed a racemic cocrystallisate, chiral HPLC analysis revealed the product **11** to be an enantioenriched mixture with an *ee* of 76%. To avoid the formation of the methanol acetal **10**, we changed the solvent to MeCN, which enabled the isolation of product **11** in 65% yield and an *ee* of 65% (Figure 3c). This aldehyde **11** ((5*S*,6*R*,7*S*,7a*R*)‐7a‐benzyl‐3,3‐dimethyl‐7‐phenyl‐5‐((*E*)‐styryl)‐1‐thioxohexahydro‐1*H*‐pyrrolo[1,2‐c]imidazole‐6‐carbaldehyde) was converted into the acetal **10** in MeOH under acidic conditions at 60 °C (Figure S4). In comparison, the reaction with the MacMillan catalyst **(*S*)‐1** showed no conversion to the *N*‐methylated derivative of **11** even after 7 d. Only minor amounts of the bicyclic product were detected by highly sensitive HRMS analysis (Figure S5).

**FIGURE 4:**
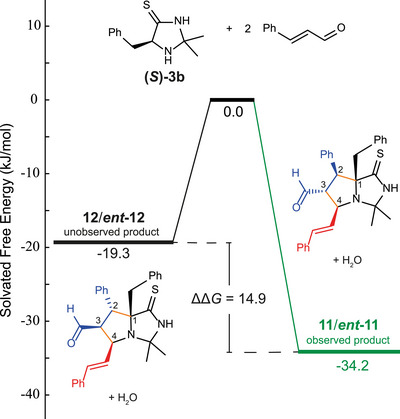
Thermodynamic energetic profile of the formation of aldehyde‐endo product **11** and its aldehyde‐*exo* diastereomer **12** from imidazoline‐4‐thione **(*S*)‐3b** and two units of cinnamaldehyde based on DFT calculations. Numbering of the newly formed ring does not correspond to IUPAC nomenclature.

Next, we investigated the mechanism and origin of the stereoselectivity in the formation of the unusual bicycle **11** by DFT calculations. First, we were interested in the diastereoselectivity of the reaction, specifically the exclusive formation of the aldehyde‐*endo* products **11** and its enantiomer ***ent*‐11** over the aldehyde‐*exo* products **12** and ***ent‐*12** (see Figure 4). To investigate a potential thermodynamic explanation, the structure of the reactants (cinnamaldehyde, **Im4t**) and the products (diastereomers **11**, **12,** and water) were optimized in ORCA 6.0.1 [25], using the PBEh‐3c composite method [26] and implicit solvation in acetonitrile [27, 28] with accurate electronic energies obtained from single‐point calculations at the ωB97X‐D4 [29, 30, 31]/def2‐QZVP [32] level of theory. The obtained geometries are given in the Supporting Information. Comparison of the energies confirms that the obtained aldehyde‐*endo* enantiomers **11**/***ent*‐11** are indeed thermodynamically favored over the aldehyde‐*exo* products **12**/***ent*‐12** with a considerable ΔΔ*G* of 14.9 kJ/mol (Figure 4).

From a structural viewpoint, this energetic difference can be attributed to an unfavorable pseudo‐axial orientation of the phenyl ring in the 2‐position of product **12**. This comes with an increased steric crowding on the *endo*‐face of the annulated ring system when compared to structure **11**, in which the smaller aldehyde moiety is oriented to the *endo*‐face (Figure S6).

**FIGURE 5:**
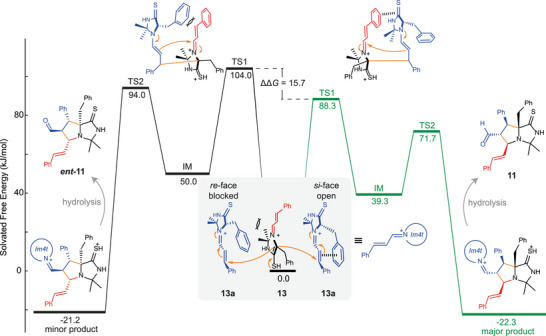
Energetic profile of the cyclization of **13** with **13a** based on DFT calculations. The endpoints are the Im4t‐condensed derivatives of products **11** and ***ent*‐11**. TS1 and TS2 denote the first and second transition state, respectively, while IM denotes the intermediate.

Next, we turned to an investigation of the reaction mechanism and, correspondingly, the origin of the experimentally observed enantioselectivity. Structurally, the product consists of **Im4t**‐activated cinnamaldehyde to which another unit of cinnamaldehyde (marked blue in Figure 4) is attached at the α‐position of the thiolactam. This motive indicates the involvement of thioenol‐intermediate **13** as one of the reactants (see Figure 5). Since the α‐position in thiolactams is more acidic compared to lactams, a higher degree of tautomerization is reached under acidic catalysis [33, 34]. This planar thioenol **13** is achiral, meaning another chiral component is required to induce the stereoselectivity observed in the reaction. We assume, analogous to the organocatalyzed Diels–Alder reaction, another activated cinnamaldehyde **13a** to be involved as a chiral auxiliary, whose **Im4t** unit is hydrolyzed after cyclization to give the final product. This mechanistic proposal was the basis of the computational study of the mechanism for the formation of bicycle **11** and its enantiomer ***ent*‐11**.

Simultaneously scanning the formed bonds (marked orange in Figure 4) in reverse and geometric optimization of the transition state indicated a two‐step reaction process. IRC following at the such obtained first transition state (TS1) to an intermediate (IM) and a nudged elastic band algorithm‐based search led to the second transition state (TS2). Mechanistically, the thus obtained pathway starts with a Michael‐type addition of thioenol **13** at the α‐position to the thiolactam to the activated γ‐position of the iminium‐ion **13a**. This leads to the intermediate (IM), which then cyclizes *via* a nucleophilic back‐attack of the formed enamine at the electrophilic iminium center, forming the bicycle **11** (Figure 5).

**FIGURE 6:**
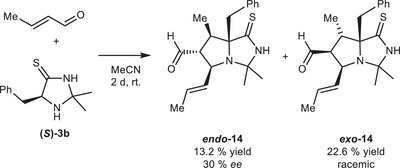
Test reaction of **(*S*)‐3a** with *trans*‐crotonaldehyde, giving a mixture of ***endo*‐14** and ***exo*‐14** with low stereoselectivity.

For both products, the activation barrier for the first attack (TS1) was higher than for the second cyclization step (TS2), revealing the Michael‐type addition as the likely rate determining step. When comparing the relative energies between the routes leading to product **11** and ***ent*‐11**, all barriers are higher for the latter (minor enantiomer). However, the diastereomeric products of **11** and ***ent*‐11,** before hydrolysis, are comparable in energy (ΔΔ*G*  =  1.1 kJ/mol). Thus, the enantioselectivity arises from the differing kinetic profile of the reaction pathway. Especially the barrier for the rate determining Michael‐addition is significantly higher in the ***ent*‐11** than in the **11** transition state (104.0 vs. 88.3 kJ/mol; *ΔΔG*_TS1_ = 15.7 kJ/mol). This difference can be reasoned structurally by examining the *re*‐ and *si*‐face of the Michael acceptor **13a**. In analogy to observations by MacMillan et al. [4, 9, 11, 35], the *re*‐face of the electrophile is shielded by the benzyl group at the chiral center, while the *si*‐face remains open to nucleophilic attack. This favoring of a *si*‐attack is then also visible in the TS1 of both pathways as a steric interaction of the “benzyl group” with the cinnamaldehyde subunit of the electrophile. (Figure S7–8). Furthermore, the TS1 toward **11** shows a favorable π‐stacking of the cinnamaldehyde‐aryl‐unit in the electrophile **13a** and the C═S bond of nucleophile **13**, whereas this interaction is blocked by the directing benzyl group for the TS1 toward ***ent‐*11**. These two factors are thus the likely causes for the observed enantioselectivity of the bicyclization. To test the proposed influence of the π‐stacking of cinnamaldehyde, we conducted another reaction using crotonaldehyde instead. Analysis of the product mixture revealed unconsumed thiolactame **(*S*)‐3b**, and only trace amounts of the desired monoaryl bicyle **14**. The low yield is presumably caused by the decomposition of the crotonaldehyde. A modified procedure without acid catalyst and with an excess of crotonaldehyde (Figure 6) resulted in a mixture of ***exo*‐14** and ***endo*‐14** (*exo*/*endo* 1.7 : 1, 36 % yield). In comparison, when using cinnamaldehyde, only the aldehyde‐*endo* product **11**/***ent*‐11** was observed. In addition, the enantioselectivity of the reaction was very low, since ***exo*‐14** was racemic and ***endo*‐14** showed only 30% *ee*. Therefore, we conclude that the stereoselectivity is highly dependent on the substrate structure, where the *endo*/*exo* selectivity and the enantioselectivity are both strongly influenced by secondary π‐interactions of the aryl‐substrate and the catalyst.

## 3 Conclusion

In conclusion, we demonstrated a simplified, modular route toward prebiotically plausible and catalytically active chiral imidazolidine‐4‐thiones based on the selection of an amino acid and a carbonyl. These structures enabled not only stereoselective Diels–Alder reactions of cyclopentadiene with cinnamaldehyde with high to medium *ee* and yield, depending on the substitution pattern.

We found that the first generation MacMillan catalysts **(*S*)‐1** and **(*R*)‐1** performed best (90% yield and 94% *ee*) together with the thionated form **(*S*)‐3a** (84% yield, up to 90% *ee*). The introduction of a *t*‐Bu‐group in *cis*‐/*trans*‐**3c** improved selectivity toward the (2*S*)‐*exo*‐**2** product, but at the cost of a lowered yield of 51–57 %. *S*‐alkylated catalysts **8** and **9** performed worse due to possible decomposition (as low as 40% yield, 77% *ee*). When the *N^3^‐*methyl group was omitted in the catalyst, we observed a decreased yield and *ee* (entry 4, 76% yield, 81% *ee*).

Serendipitously, we discovered a novel self‐cyclization when the diene was omitted. The bicyclic product aldehyde **11**, notable for its *N*‐heterobicyclic core structure with four consecutive stereocenters, was formed in 65% yield and an *ee* of up to 76%. DFT calculations revealed the aldehyde‐*endo* enantiomers as the thermodynamic products over the unobserved *exo* products. A reaction mechanism was proposed, involving a two‐step Michael‐addition of a thioenol lactam **13** to the activated cinnamaldehyde **13a**, followed by intramolecular cyclization through an activated enamine. The enantioselectivity was explained by steric hindrance in the conjugate addition step (TS1).

These findings show that imidazolidine‐4‐thiones exhibit a different reactivity compared to the classical MacMillan catalyst, originating from thionation. They fulfil a triple role in the form of a heterocyclic building block, a chiral auxiliary and an activator to form complex structures in a stereospecific manner. The application of this selective mechanism should be extended to more building blocks and conditions, to study general applicability and selectivity. Future research needs to be performed on this topic, to fully harness the potential of imidazolidine‐4‐thiones.

## Conflicts of Interest

The authors declare no conflict of interest.

## Supporting information



The authors have cited additional references within the Supporting Information [36, 37, 38, 39, 40, 41, 42, 43, 44, 45, 46, 47, 48]. **Supporting File 1**: chem70572‐sup‐0001‐SuppMat.docx

**Supporting File 2**: chem70572‐sup‐0002‐SuppMat.cif

**Supporting File 3**: chem70572‐sup‐0003‐SuppMat.docx

## Data Availability

The data that support the findings of this study are available in the supplementary material of this article.
