# Supplementary material for: Dual‐Mode Thio‐MacMillan Organocatalysts: Stereoselective Diels–Alder Reactions or Sacrificial Self‐Cyclization to N‐Bridged Bicyclic Lactams
Source: Chemistry. 2025 Dec 18;32(5):e03017. doi: 10.1002/chem.202503017 (PMC12865155; doi:10.1002/chem.202503017)
Supplement: Supplementary file 1 — The authors have cited additional references within the Supporting Information [36, 37, 38, 39, 40, 41, 42, 43, 44, 45, 46, 47, 48]. Supporting File 1: chem70572‐sup‐0001‐SuppMat.docx [file CHEM-32-e03017-s003.docx]

Supplementary Information

Dual-Mode Thio-MacMillan Organocatalysts:
Stereoselective Diels-Alder Reactions or Sacrificial Self-Cyclization to *N*-Bridged Bicyclic Lactams

Marian S. R. Ebeling,^a,+^ Luca V. Parziale,^a,+^ Marc Sachsenhauser,^a^ Christoph J. B. Seifert,^a^ Nathalie J. Kurrle,^a^ and Oliver Trapp*^a,b^

^a^ Department of Chemistry, Ludwig-Maximilians-University Munich, Munich, Germany.

^b^ Max Planck Institute for Astronomy, Heidelberg, Germany.

^+^ These authors contributed equally to this work

* Corresponding author: Email: [oliver.trapp@cup.uni-muenchen.de](mailto:oliver.trapp@cup.uni-muenchen.de)

**List of numbered structures**

|  | **(*S*)-1** |  | **4** |  | | **12** |
| --- | --- | --- | --- | --- | --- | --- |
|  | **(*R*)-1** |  | **5** |  | | ***ent*-12** |
|  | **(2*S*)-*endo*-2** |  | **6** |  | | **13** |
|  | **(2*S*)-*exo*-2** |  | **7** |  | | **13a** |
|  | **(*S*)-3a** |  | **8** |  | | **endo-14** |
|  | **(*S*)-3b** |  | **9** |  | **exo-14** | |
|  | **(*R*)-3b** |  | **10** |  | | |
|  | ***trans*-3c** |  | **11** |  |  |  |
|  | ***cis*-3c** |  | ***ent*-11** |  |  |  |

**Contents**

[1 General experimental details 6](#_Toc207204706)

[1.1 Techniques 6](#_Toc207204707)

[1.2 Solvents, reagents and starting materials 6](#_Toc207204708)

[1.3 Flash column chromatography 6](#_Toc207204709)

[1.4 Nuclear magnetic resonance spectroscopy 6](#_Toc207204710)

[1.5 High resolution mass spectroscopy 7](#_Toc207204711)

[1.6 High performance liquid chromatography 7](#_Toc207204712)

[1.7 Gas chromatography 7](#_Toc207204713)

[1.8 Ultrapure water 7](#_Toc207204714)

[1.9 X-ray crystallography 7](#_Toc207204715)

[2 Synthetic, spectroscopic, and analytical data 8](#_Toc207204716)

[2.1 Pathway to (*S*/*R*)-1-amino-3-phenyl-1-thioxopropan-2-aminium chloride **7a** 8](#_Toc207204717)

[2.2 Pathway to (*S*)-1-(methylamino)-3-phenyl-1-thioxopropan-2-aminium chloride **7b** 14](#_Toc207204718)

[2.3 Synthesis of different imidazolidine-4-thione organocatalysts 17](#_Toc207204719)

[2.4 Diels-Alder reactions 24](#_Toc207204720)

[2.5 Step-wise cyclization of *trans*-cinnamaldehyde and imidazolidine-4-thione **(*S*)-3b** 33](#_Toc207204721)

[3 Computational methods 39](#_Toc207204722)

[3.1 General parameters 39](#_Toc207204723)

[3.2 ORCA 6.0.1 Keywords 40](#_Toc207204724)

[3.3 Energy calculation 41](#_Toc207204725)

[3.4 Energies of the calculated geometries 41](#_Toc207204726)

[3.5 Geometries 41](#_Toc207204727)

[3.6 Supplementary figures 42](#_Toc207204728)

[4 Spectra and X-ray structures 44](#_Toc207204729)

[4.1 NMR spectra of synthesized compounds 44](#_Toc207204730)

[4.2 X-ray crystallography 65](#_Toc207204731)

[5 Literature 69](#_Toc207204732)

Supplementary Tables

[**Table S1** Molar ratios of Diels-Alder product **2** to internal standard C17 and resulting ratios by integration of GC peaks. 28](#_Toc207208244)

[**Table S2** Overview of all catalysts tested for Diels-Alder reaction of trans-cinnamaldehyde and cyclopenta-1,3-diene according to GP1 or GP2. All reaction entries were performed thrice (entry a, b, c). The yield was determined by referencing to the internal standard n-heptadecane in the GC-analysis. The exo:endo ratio and all ee were determined by GC. 30](#_Toc207208245)

[**Table S3** Raw data integrals of Diels-Alder catalyst screening of C17, (2R)-endo-**2**, (2R)-exo-**2**, (2S)-endo-**2** and (2S)-exo-**2.** 32](#_Toc207208246)

[**Table S4:** Total energy, electronic energy, thermal corrections and imaginary modes of the geometries calculated in this work. 41](#_Toc207208247)

Supplementary Figures

[**Figure S1** Exemplary GC-Analysis of the crude Diels-Alder products with n-heptadecane (C17) at 80.1 min. 27](#_Toc207208248)

[**Figure S2** Plot of **Table S1**: molar ratio of product **2**/C17 against GC ratio (product **2**/C17) and the resulting linear fit. 29](#_Toc207208249)

[**Figure S3** Crystal structure of aldehyde **11**. Compound **11** and its enantiomer **ent-11** cocrystallize as a racemate. Only component **11** is shown above. 35](#_Toc207208250)

[**Figure S4** Stacked NMR spectra of the acetal protection of the aldehyde **11** (no. 3) to the acetal **10**. Crude product signals (no. 1) match with the reference spectrum of **10** (no. 2). 37](#_Toc207208251)

[**Figure S5** HRMS spectrum of a reaction mixture of **(S)-1** and trans-cinnamaldehyde in MeOH/H_2_O (95:05, 0.4 M) after 7 d. 38](#_Toc207208252)

[**Figure S6** Visualisation of diastereomeric products **12** and **11**. 42](#_Toc207208253)

[**Figure S7** Visualisation of electrophilic iminium ion **13a** with blocked re-face. 42](#_Toc207208254)

[**Figure S8** Visualisation of transition states (TS1) of leading to **ent-11** and **11**. 43](#_Toc207208255)

List of abbreviations

| DBU | 1,8-Diazabicyclo[5.4.0]undec-7-ene |
| --- | --- |
| DCM | Dichloromethane |
| DMSO | Dimethyl sulfoxide |
| *p-*TsOH | *para*-Toluenesulfonic acid |
| *dr* | Diastereomeric ratio |
| *ee* | Enantiomeric excess |
| EI | Electron ionisation |
| ESI | Electrospray ionisation |
| FT | Film thickness |
| FID | Flame Ionisation Detector |
| GC | Gas chromatography |
| HOMO | Highest occupied molecular orbital |
| HPLC | High performance liquid chromatography |
| HRMS | High resolution mass spectrometry |
| ID | Inner diameter |
| IRC | Intrinsic reaction coordinate |
| LUMO | Lowest unoccupied molecular orbital |
| NMR | Nuclear magnetic resonance |
| R*_f_* | Retention factor |
| SOMO | Singly occupied molecular orbital |
| THF | Tetrahydrofuran |
| TFA | Trifluoroacetic acid |
| TLC | Thin layer chromatography |
| UV | Ultraviolet radiation |

# General experimental details

## Techniques

Air and moisture sensitive reactions were carried out in an argon atmosphere (Ar 5.0) under exclusion of air and moisture. All glassware was flame-dried prior to use and standard Schlenk techniques were employed. Syringes to transfer liquids and solutions were flushed thrice with argon prior to use; solids were added under argon flow. If not stated otherwise, the yields given refer to isolated and purified products.

## Solvents, reagents and starting materials

Anhydrous THF, DCM and Et_2_O were taken from a solvent purification system (MB SPS 5 from MBRAUN) under argon atmosphere. All other solvents and chemicals were purchased from commercial sources and stored according to the respective instructions. Isolated compounds were stored under argon and, if needed, at 5 °C or −20 °C.

## Flash column chromatography

Preparative purification by flash column chromatography was performed using silica gel (technical grade, pore size: 60 Å, particle size: 35-70 μm). Thin-layer chromatography was performed using pre-coated polyester sheets (0.2 mm silica gel with fluorescent indicator). For visualization, the TLC plates were analysed under UV-light or stained by submerging in an aqueous solution of potassium permanganate and developed by heating.

For automated flash column chromatography, a Puriflash XS520 Plus, equipped with a 200-400 nm UV-detector, from Advion Interchim Scientific was used.

## Nuclear magnetic resonance spectroscopy

^1^H- and ^13^C-NMR spectra were measured on a Bruker Avance III HD 400 MHz spectrometer with a CryoProbe and a Bruker Avance III HD 800 MHz spectrometer with a CryoProbe operating at 400 MHz and 800 MHz for proton nuclei and 100 MHz and 200 MHz for carbon nuclei, respectively. For NMR data processing the Mestrelab Resarch software MestReNova v15.0.0-34764 was used. Chemical shifts were calibrated using the residual NMR solvent signals: CDCl_3_: ^1^H NMR *δ*[ppm] = 7.26, ^13^C NMR *δ*[ppm] = 77.16; CD_2_Cl_2_ ^1^H NMR *δ*[ppm] = 5.32, ^13^C NMR *δ*[ppm] = 53.84; CD_3_CN ^1^H NMR *δ*[ppm] = 1.94, ^13^C NMR *δ*[ppm] = 118.26; DMSO-*d*_6_: ^1^H NMR *δ*[ppm] = 2.50, ^13^C NMR *δ*[ppm] = 39.52.^[36]^ All ^13^C spectra were recorded with ^1^H decoupling. The following abbreviations were used for multiplicity: s (singlet), d (doublet), t (triplet), q (quartet), p (pentet), m (multiplet) and br. s (broad singlet). Coupling constants through n bonds (^n^*J*) are provided in Hertz [Hz]. Magnetically inequivalent hydrogen atoms of the same carbon/nitrogen atom are differentiated by *a* and *b*. The assignment of the signals was realized by two-dimensional NMR spectroscopy (^1^H-^1^H-COSY, ^1^H-^13^C-HSQC, ^1^H-^13^C-HMBC, ^1^H-^1^H-NOESY).

## High resolution mass spectroscopy

High resolution mass spectrometric (HRMS) analysis was performed using a Thermo Scientific Q Exactive Plus mass spectrometer coupled to electrospray ionization (ESI). The mass spectrometer was operated with a scan range of 100 to 750 *m/z* in positive mode with a resolution of 140,000 and an automatic gain control target of $1\cdot{10}^{6}$. The auxiliary gas heater was set to 150 °C, the capillary temperature was set to 250 °C and spray voltage was set to 3.5 kV. For measurements, samples were injected in the flow of H_2_O/iPrOH/formic acid (80:20:0.5%).

## High performance liquid chromatography

HPLC measurements were performed on an Agilent Series 1200 Infinity system equipped with a high-performance autosampler model HiP-ALS SL+ and a G1315D photodiode array detector. The separation of different imidazolidine-4-thione derivatives and precursors was performed with a CHIRALPAK IC (4.6 mm Ø x 250 mm, particle size 5 μm) or CHIRALPAK IC-3 (4.6 mm Ø x 150 mm, particle size 3 μm) from Daicel at 20 °C and a flow of 1.00 mL/min. 5 μL of the sample was injected and eluted using a mixture of *n*-hexane/*i-*PrOH (80/20). The peaks were detected at 270 nm.

## Gas chromatography

Gas chromatography analysis was performed on a Trace GC 2000 with an AS 3000 auto sampler and an ISQ mass spectrometer with an EI-source (70 eV). Injection used a split/splitless injector in split mode (splitflow 30 mL/min) at 250 °C. Analytes were separated on a chirasil‑β‑dex column (l = 10 m, ID = 250 μm, FT = 500 nm). Detection was performed by FID with a base temperature of 250 °C, air flow 350 mL/min, H_2_ flow 35 mL/min and makeup gas flow 30 mL/min.

## Ultrapure water

Ultrapure water used for experiments and measurements was obtained from a Puranity PU 15 UV from VWR.

## X-ray crystallography

The X-ray intensity data were measured on a Bruker D8 Venture TXS system equipped with a multilayer mirror monochromator and a Mo Kα rotating anode X-ray tube (*λ* = 0.71073 Å). The frames were integrated with the Bruker SAINT software package.^[37]^ Data were corrected for absorption effects using the Multi-Scan method (SADABS).^[38]^ The structure was solved and refined using the Bruker SHELXTL Software Package.^[39]^ All C-bound hydrogen atoms have been calculated in ideal geometry riding on their parent atoms, the N-bound hydrogen atoms have been refined freely. The figures have been drawn at the 25 % ellipsoid probability level.^[40]^

# Synthetic, spectroscopic, and analytical data

## Pathway to (*S*/*R*)-1-amino-3-phenyl-1-thioxopropan-2-aminium chloride **7a**

### Synthesis of *tert*-butyl (*S*)-(1-amino-1-oxo-3-phenylpropan-2-yl)carbamate **(*S*)-5a**

Boc-l-Phe-OH (7.96 g, 30.0 mmol, 1.00 eq.) was dissolved in dry THF (100 mL) under argon and cooled to 0 °C. After the addition of NEt_3_ (12.5 mL, 90.0 mmol, 3.00 eq.), ethyl chloroformate (4.00 mL, 42.0 mmol, 1.40 eq.) was slowly added at 0 °C and stirred for 30 min. Then aq. NH_4_Cl (1 m; 45.0 mL, 45.0 mmol, 1.50 eq.) was added and stirred for 30 min. The reaction mixture was partitioned between EtOAc (700 mL) and water (100 mL). The aqueous phase was extracted with EtOAc (3 x 100 mL) and the organic phases were combined and washed with water (100 mL) and aq. sat. NaCl (100 mL). The organic phase was dried over Na_2_SO_4_ and concentrated *in vacuo*. The product **(*S*)-5a** (7.54 g, 28.5 mmol, 95 %) was obtained as a white solid.^[22]^

**R*_f_*** (*c*-Hex/EtOAc 1:1) = 0.24.

**^1^H NMR** (400 MHz, CD_2_Cl_2_, ppm): δ = 7.34 - 7.19 (m, 5H, H^15, 16, 17, 18, 19^), 5.91 (br. s, 1H, *N*H^6a/b^), 5.53 (br. s, 1H, *N*H^6a/b^), 5.14 - 4.99 (m, 1H, *N*H^2^), 4.34 (d, *J*= 8.5 Hz, 1H, H^3^), 3.09 (dd, *J* = 13.9, 6.5 Hz, 1H, H^4a^), 2.99 (dd, *J* = 13.9, 7.1 Hz, 1H, H^4b^), 1.38 (s, 9H, H^11, 12, 13^).

**^13^C NMR** (101 MHz, CD_2_Cl_2_, ppm): δ = 173.8 (1C, C^1^), 155.7 (1C, C^7^), 137.3 (1C, C^14^), 129.7 (2C, C^15, 19^), 128.9 (2C, C^16, 18^), 127.2 (1C, C^17^), 80.3 (1C, C^10^), 55.7 (1C, C^3^), 38.5 (1C, C^4^), 28.3 (3C, C^11, 12, 13^).

**HRMS** (ESI): found: [*M*+H]^+^ 265.1545, C_14_H_21_N_2_O_3_^+^ requires 265.1547.

**HPLC** (IC, *n*-hexane/*i*-PrOH 80:20): *t*_R_ = 16.0 min.

### Synthesis of *tert*-butyl (*R*)-(1-amino-1-oxo-3-phenylpropan-2-yl)carbamate **(*R*)-5a**

Boc-d-Phe-OH (796 mg, 3.00 mmol, 1.00 eq.) was dissolved in dry THF (10 mL) under argon and cooled to 0 °C. After the addition of NEt_3_ (1.25 mL, 9.00 mmol, 3.00 eq.), ethyl chloroformate (0.400 mL, 4.20 mmol, 1.40 eq.) was slowly added at 0 °C and stirred for 30 min. Then aq. NH_4_Cl (1 m, 4.50 mL, 4.50 mmol, 1.50 eq.) was added and stirred for 30 min. The reaction mixture was partitioned between EtOAc (70 mL) and water (20 mL). The aqueous phase was extracted with EtOAc (3 x 20 mL) and the organic phases were combined and washed with water (20 mL) and aq. sat. NaCl (20 mL). The organic phase was dried over Na_2_SO_4_ and concentrated *in vacuo*. The product **(*R*)-5a** (713 mg, 2.70 mmol, 90 %) was obtained as a white solid.^[22]^

**R*_f_*** (*c*-Hex/EtOAc 1:1) = 0.24.

**^1^H NMR** (400 MHz, CD_2_Cl_2_, ppm): δ = 7.37 - 7.15 (m, 5H, H^15, 16, 17, 18, 19^), 6.04 (br. s, 1H, *N*H^6a/b^), 5.76 (br. s, 1H, *N*H^6a/b^), 5.15 (d, *J*= 8.0 Hz, 1H, *N*H^2^), 4.39 - 4.31 (m, 1H, H^3^), 3.09 (dd, *J*= 13.9, 6.4 Hz, 1H, H^4a^), 2.98 (dd, *J*= 14.2, 7.6 Hz, 1H, H^4b^), 1.38 (s, 9H, H^11, 12, 13^).

**^13^C NMR** (101 MHz, CD_2_Cl_2_, ppm): δ = 174.4 (1C, C^1^), 155.9 (1C, C^7^), 137.5 (1C, C^14^), 129.9 (2C, C^15, 19^), 129.1 (2C, C^16, 18^), 127.4 (1C, C^17^), 80.5 (1C, C^10^), 55.8 (1C, C^3^), 38.7 (1C, C^4^), 28.5 (3C, C^11, 12, 13^).

**HRMS** (ESI): found: [*M*+H]^+^ 265.1523, C_14_H_21_N_2_O_3_^+^ requires 265.1547.

**HPLC** (IC, *n*-hexane/*i*-PrOH 80:20): *t*_R_ = 15.7 min.

### Synthesis of *tert*-butyl (*S*)-(1-amino-3-phenyl-1-thioxopropan-2-yl)carbamate **(*S*)-6a**

**(*S*)-5a** (7.93 g, 30.0 mmol, 1.00 equiv.) and Lawesson's reagent (6.31 g, 15.6 mmol, 0.52 equiv.) were dissolved in dry THF (50 mL) and were stirred under argon for 2 h. The reaction mixture was partitioned between sat. aq. NaHCO_3_ (100 mL) and DCM (250 mL). The aqueous phase was extracted with DCM (3 x 250 mL). The organic phase was dried over Na_2_SO_4_ and concentrated *in vacuo*. The addition of Et_2_O (200 mL) precipitated unwanted side products before the column. After filtration and reconcentration *in vacuo*, the crude product was purified by flash column chromatography (*n*-pentane/EtOAc, 5:2) to obtain **(*S*)-6a** (6.55 g, 23.4 mmol, 78 %) as a white solid.^[23]^

**R*_f_*** (*n*-pentane/EtOAc 3:1) = 0.25.

**^1^H NMR** (400 MHz, CD_3_CN, ppm): δ = 8.26 (br. s, 1H, *N*H^12a/b^), 8.13 (br. s, 1H, *N*H^12a/b^), 7.34 - 7.19 (m, 5H, H^6, 7, 8, 9, 10^), 5.77 (d, *J*= 8.6 Hz, 1H, *N*H^2^), 4.61 (td, *J*= 8.8, 5.4 Hz, 1H, H^3^), 3.19 (dd, *J*= 13.8, 5.3 Hz, 1H, H^4a^), 2.91 (dd, *J*= 13.8, 9.1 Hz, 1H, H^4b^), 1.34 (s, 9H, H^17, 18, 19^).

**^13^C NMR** (201 MHz, CD_3_CN, ppm): δ = 210.1 (1C, C^1^), 156.1 (1C, C^13^), 138.4 (1C, C^5^), 130.2 (2C, C^6, 10^), 129.3 (2C, C^7, 9^), 127.6 (1C, C^8^), 80.1 (1C, C^16^), 62.4 (1C, C^3^), 41.9 (1C, C^4^), 28.5 (3C, C^17, 18, 19^).

**HRMS** (ESI): found: [*M*+H]^+^ 281.1317, C_14_H_21_N_2_O_2_S^+^ requires 281.1318.

**HPLC** (IC, *n*-hexane/*i*-PrOH 80:20): *t*_R_ = 5.1 min.

### Synthesis of *tert*-butyl (*R*)-(1-amino-3-phenyl-1-thioxopropan-2-yl)carbamate **(*R*)-6a**

**(*R*)-5a** (1.43 g, 5.40 mmol, 1.00 equiv.) and Lawesson's reagent (1.14 g, 2.81 mmol, 0.52 equiv.) were dissolved in dry THF (0.4 m, 13.5 mL) and were stirred under argon for 2 h. The reaction mixture was partitioned between sat. aq. NaHCO_3_ (30 mL) and DCM (50 mL). The aqueous phase was extracted with DCM (3 x 30 mL). The organic phase was dried over Na_2_SO_4_ and concentrated *in vacuo*. The addition of Et_2_O (50 mL) precipitated unwanted side products before the column. After filtration and reconcentration *in vacuo*, the crude product was purified by flash column chromatography (*n*-pentane/EtOAc, 5:2) to obtain **(*R*)-6a** (1.23 g, 4.40 mmol, 81 %) as a white solid.^[23]^

**R*_f_*** (*n*-pentane/EtOAc 3:1) = 0.25.

**^1^H NMR** (400 MHz, CD_2_Cl_2_, ppm): δ = 7.50 (br. s, 2H, *N*H^12^), 7.35 - 7.21 (m, 5H, H^6, 7, 8, 9, 10^), 5.28 (br. s, 1H, *N*H^2^), 4.68 - 4.58 (m, 1H, H^3^), 3.18 (dd, *J*= 13.0, 6.8 Hz, 1H, H^4a^), 3.08 (dd, *J*= 13.7, 7.8 Hz, 1H, H^4b^), 1.38 (s, 9H, H^17, 18, 19^).

**^13^C NMR** (101 MHz, CD_2_Cl_2_, ppm): δ = 209.0 (1C, C^1^), 155.7 (1C, C^13^), 137.1 (1C, C^5^), 129.7 (2C, C^6, 10^), 129.0 (2C, C^7, 9^), 127.4 (1C, C^8^), 80.7 (1C, C^16^), 61.8 (1C, C^3^), 41.9 (1C, C^4^), 28.4 (3C, C^17, 18, 19^).

**HRMS** (ESI): found: [*M*+H]^+^ 281.1316, C_14_H_21_N_2_O_2_S^+^ requires 281.1318.

**HPLC** (IC, *n*-hexane/*i*-PrOH 80:20): *t*_R_ = 5.6 min.

### Synthesis of (*S*)-1-amino-3-phenyl-1-thioxopropan-2-aminium chloride **(*S*)-7a**

**(*S*)-6a** (4.44 g, 15.8 mmol, 1.00 equiv.) was dissolved in dry DCM (50 mL) under argon. HCl in dioxane (4.0 m, 15.84 mL, 63.3 mmol, 4.00 equiv.) was added and the solution stirred for 3 h. The solution was filtered off and the precipitate washed with dry Et_2_O (5 × 25 mL) under argon. The precipitate was dried under high vacuum. The product **(*S*)-7a** (3.12 g, 14.4 mmol, 91 %) was obtained as a white solid and stored under argon.

**^1^H NMR** (400 MHz, DMSO-*d*_6_, ppm): δ = 9.92 (br. s, 1H, *N*H^11a/b^), 9.83 (br. s, 1H, *N*H^11a/b^), 8.49 (br. s, 3H, *N*H^10^), 7.33 - 7.22 (m, 5H, H^5,6,7,8,9^), 4.32 (d, *J* = 7.8 Hz, 1H, H^2^), 3.15 (dd, *J* = 13.3, 6.1 Hz, 1H, H^3a^), 3.06 (dd, J = 13.3, 8.3 Hz, 1H, H^3b^).

**^13^C NMR** (101 MHz, DMSO-*d*_6_, ppm): δ = 200.9 (1C, C^1^), 134.9 (1C, C^4^), 129.5 (1C, C^5, 9^), 128.4 (1C, C^6, 8^), 127.1 (1C, C^7^), 58.2 (1C, C^2^), 39.4 (1C, C^3^).

**HRMS** (ESI): found: [*M*−Cl]^+^ 181.0793, C_9_H_13_N_2_S^+^ requires 181.0794.

### Synthesis of (*R*)-1-amino-3-phenyl-1-thioxopropan-2-aminium chloride **(*R*)-7a**

**(*R*)-6a** (4.44 g, 15.8 mmol, 1.00 equiv.) was dissolved in dry DCM (50 mL) under argon. HCl in dioxane (4.0 m, 15.84 mL, 63.3 mmol, 4.00 equiv.) was added and the solution stirred for 3 h. The solution was filtered off and the precipitate washed with dry Et_2_O (5 × 25 mL) under argon. The precipitate was dried under high vacuum. The product **(*R*)-7a** (3.01 g, 13.9 mmol, 86 %) was obtained as a white solid and stored under argon.

**^1^H NMR** (400 MHz, DMSO-*d*_6_, ppm): δ = 9.93 (br. s, 1H, *N*H^11a/b^), 9.73 (br. s, 1H, *N*H^11a/b^), 8.41 (br. s, 3H, *N*H^10^), 7.37 - 7.19 (m, 5H, H^5, 6, 7, 8, 9^), 4.26 (s, 1H, H^2^), 3.09 (qd, *J*= 13.4, 7.2 Hz, 2H, H^3^).

**^13^C NMR** (101 MHz, DMSO-*d*_6_, ppm): δ = 201.0 (1C, C^1^), 134.8 (1C, C^4^), 129.5 (2C, C^5, 9^), 128.4 (2C, C^6, 8^), 127.1 (1C, C^7^), 58.3 (1C, C^2^), 39.1 (1C, C^3^).

**HRMS** (ESI): found: [*M*−Cl]^+^ 181.0793, C_9_H_13_N_2_S^+^ requires 181.0794.

## Pathway to (*S*)-1-(methylamino)-3-phenyl-1-thioxopropan-2-aminium chloride **7b**

### Synthesis of *tert*-butyl (*S*)-(1-(methylamino)-3-phenyl-1-thioxopropan-2-yl)carbamate **(*S*)-5b**

Boc-d-Phe-OH (2.65 g, 10.0 mmol, 1.00 eq.) was dissolved in dry THF (40 mL) under argon and cooled to 0 °C. NEt_3_ (4.18 mL, 30.0 mmol, 3.00 eq.) was added, then ethyl chloroformate was added dropwise under vigorous stirring. After 30 min at 0 °C, an aqueous solution of MeNH_3_Cl (1.00 m, 15.0 mL, 15.0 mmol, 1.50 eq.) was added and the solution stirred for 30 min at 0 °C. The reaction mixture was partitioned between H_2_O (50 mL), sat. aq. NaCl (20 mL) and EtOAc (150 mL). The aqueous phase was extracted with EtOAc (4 x 150 mL), the combined organic phase was dried over Na_2_SO_4_ and was concentrated *in vacuo*. The crude product was purified by flash column chromatography (puriflash column: PF-15SIHP-F0120, *c*-Hex/EtOAc, 88:12 -> 6:94, over 15 column volumes) to obtain **(*S*)-5b** (2.26 g, 8.12 mmol, 81 %) as a white solid.^[22]^

**R*_f_*** (*c*-Hex/EtOAc 1:1) = 0.23.

**^1^H NMR** (400 MHz, CD_2_Cl_2_, ppm): δ = 7.35 - 7.27 (m, 2H, H^17, 19^), 7.27 - 7.22 (m, 1H, H^18^), 7.22 - 7.15 (m, 2H, H^16, 20^), 5.81 (br. s, 1H, *N*H^9^), 5.00 (br. s, 1H, *N*H^2^), 4.25 (d, *J*= 7.8 Hz, 1H, H^3^), 3.06 (dd, *J*= 13.7, 6.7 Hz, 1H, H^4a^), 2.98 (dd, *J*= 13.8, 6.9 Hz, 1H, H^4b^), 2.70 (d, *J*= 4.9 Hz, 3H, H^15^), 1.38 (s, 9H, H^11, 12, 13^).

**^13^C NMR** (101 MHz, CD_2_Cl_2_, ppm): δ = 172.0 (1C, C^1^), 155.7 (1C, C^7^), 137.5 (1C, C^14^), 129.7 (2C, C^16, 20^), 128.9 (2C, C^17, 19^), 127.1 (1C, C^18^), 80.2 (1C, C^10^), 56.2 (1C, C^3^), 39.0 (1C, C^4^), 28.4 (3C, C^11, 12, 13^), 26.3 (1C, C^15^).

**HRMS** (ESI): found: [*M*+H]^+^ 279.1703, C_15_H_23_N_2_O_3_^+^ requires 279.1703.

**HPLC** (IC, *n*-hexane/*i*-PrOH 80:20): *t*_R_ = 17.5 min.

### Synthesis of *tert*-butyl (*S*)-(1-(methylamino)-3-phenyl-1-thioxopropan-2-yl)carbamate **(*S*)-6b**

**(*S*)-5b** (1.31 g, 4.72 mmol, 1.00 equiv.) and Lawesson's reagent (1.05 g, 2.59 mmol, 0.55 equiv.) were dissolved in dry THF (12 mL) and were stirred under argon for 2 h. The reaction mixture was partitioned between sat. aq. NaHCO_3_ (40 mL) and DCM (70 mL). The aqueous phase was extracted with DCM (3 x 70 mL). The organic phase was dried over Na_2_SO_4_ and concentrated *in vacuo*. The addition of Et_2_O (100 mL) precipitated unwanted side products before the column. After filtration and reconcentration *in vacuo*, the crude product was purified by flash column chromatography (*n*-pentane/EtOAc, 5:2) to obtain **(*S*)-6b** (907 mg, 3.08 mmol, 65 %) as a white solid.^[23]^

**R*_f_*** (*n*-pentane/EtOAc 3:1) = 0.35.

**^1^H NMR** (400 MHz, CD_2_Cl_2_, ppm): δ = 7.94 (br. s, 1H, *N*H^6^), 7.37 - 7.12 (m, 5H, H^16, 17, 18, 19, 20^), 5.37 (br. s, 1H, *N*H^2^), 4.57 (dtd, *J*= 8.2, 7.1, 1.2 Hz, 1H, H^3^), 3.09 (td, *J*= 13.0, 7.3 Hz, 2H, H^4a,b^), 2.99 (d, *J*= 4.8 Hz, 3H, H^15^), 1.37 (s, 9H, H^11, 12, 13^).

**^13^C NMR** (101 MHz, CD_2_Cl_2_, ppm): δ = 204.3 (1C, C^1^), 155.6 (1C, C^7^), 137.3 (1C, C^14^), 129.6 (2C, C^16, 20^), 128.9 (2C, C^17, 19^), 127.3 (1C, C^18^), 80.6 (1C, C^10^), 62.8 (1C, C^3^), 42.3 (1C, C^4^), 32.6 (1C, C^15^), 28.4 (3C, C^11, 12, 13^).

**HRMS** (ESI): found: [*M*+H]^+^ 295.1473, C_15_H_23_N_2_O_2_^S+^ requires 295.1475.

**HPLC** (IC, *n*-hexane/*i*-PrOH 80:20): *t*_R_ = 5.6 min.

### Synthesis of (*S*)-1-(methylamino)-3-phenyl-1-thioxopropan-2-aminium chloride **(*S*)-7b**

**(*S*)-6b** (907 mg, 3.08 mmol, 1.00 eq.) was dissolved in dry DCM (9 mL) under argon. Then, HCl in dioxane (4 m, 4.00 mL, 16.0 mmol, 4.00 eq.) was added and stirred for 1 h. All volatiles were removed *in vacuo,* and the crude product was washed with dry Et_2_O (3 x 20 mL) to obtain **(*S*)-7b** (573 mg, 2.48 mmol, 81 %) as a white solid.

**^1^H NMR** (400 MHz, DMSO-*d*_6_, ppm): δ = 10.88 (q, *J*= 4.7 Hz, 1H, *N*H^6^), 8.56 (br. s, 3H, *N*H^2^), 7.35 - 7.18 (m, 5H, H^9, 10, 11, 12, 13^), 4.39 (m, 1H, H^3^), 3.16 (dd, *J*= 13.2, 5.8 Hz, 1H, H^4a^), 3.06 (dd, *J*= 13.2, 8.7 Hz, 1H, H^4b^), 2.82 (d, *J*= 4.3, 3H, H^8^).

**^13^C NMR** (101 MHz, DMSO-*d*_6_, ppm): δ = 135.0 (1C, C^1^), 129.4 (2C, C^10, 12^), 128.4 (2C, C^9, 13^), 127.1 (1C, C^11^), 58.5 (1C, C^3^), 39.6 (1C, C^4^), 31.9 (1C, C^8^).

**HRMS** (ESI): found: [*M*−Cl]^+^ 195.0948, C_10_H_15_N_2_S^+^ requires 195.0950.

## Synthesis of different imidazolidine-4-thione organocatalysts

### Synthesis of (*S*)-5-benzyl-2,2,3-trimethylimidazolidine-4-thione **(*S*)-3a**

**(*S*)-7b** (529 mg, 2.29 mmol, 1.00 eq.) was neutralized with sat. aq. NaHCO_3_ and extracted with DCM (3 x 50 mL). The organic phase was dried over Na_2_SO_4_ and concentrated *in vacuo*. The free base was dissolved in MeOH (0.4 m, 5.7 mL) and *p-*TsOH (4.3 mg, 22.9 µmol, 0.01 eq.) was added. Then, acetone (3.40 mL, 45.9 mmol, 20 eq.) was added and the reaction was stirred for 4 h. All volatiles were removed *in vacuo* and the crude product purified by flash column chromatography (*c*-Hex/EtOAc, 1:1) to afford **(*S*)-3a** (582 mg, 2.45 mmol, 68 %) as a yellow oil.

**R*_f_*** (*c*-Hex/EtOAc 1:1) = 0.3.

**^1^H NMR** (400 MHz, CD_2_Cl_2_, ppm): δ = 7.34 – 7.19 (m, 5H, H^12, 13, 14, 15, 16^), 4.08 (dd, *J* = 8.4, 4.0 Hz, 1H, H^1^), 3.59 (dd, *J* = 14.3, 4.1 Hz, 1H, H^7a^), 3.14 (d, *J* = 1.0 Hz, 3H, H^8^), 2.92 (dd, *J* = 14.3, 8.5 Hz, 1H, H^7b^), 1.75 (br. s, 1H, *N*H^2^), 1.35 (s, 3H, H^10^), 1.29 (s, 3H, H^11^).

**^13^C NMR** (101 MHz, CD_2_Cl_2_, ppm): δ = 200.3 (1C, C^5^), 138.4 (1C, C^9^), 129.7 (2C, C^12, 16^), 128.8 (2C, C^13, 15^), 126.9 (1C, C^14^), 84.2 (1C, C^3^), 70.6 (1C, C^1^), 40.3 (1C, C^7^), 31.1 (1C, C^8^), 26.8 (1C, C^10^), 24.7 (1C, C^11^).

**HRMS** (ESI): found: [*M*+H]^+^ 235.1262, C_13_H_19_N_2_S^+^ requires 235.1263.

**HPLC** (IC, *n*-hexane/*i*-PrOH 80:20): *t*_R_ = 5.1 min (98.8 % *ee*).


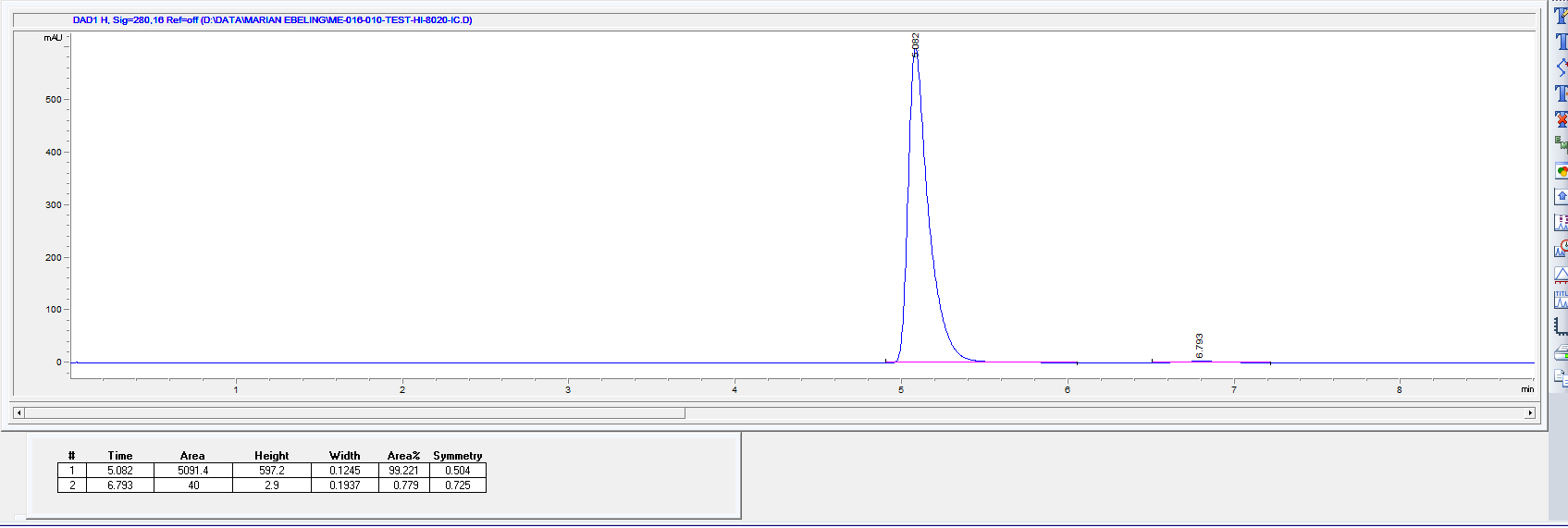


### Synthesis of (*S*)-5-benzyl-2,2-dimethylimidazolidine-4-thione **(*S*)-3b**

**(*S*)-7a** (268 mg, 1.24 mmol, 1.00 eq.) and *p-*TsOH (2.4 mg, 12.4 µmol, 0.01 eq.) were dissolved in MeOH (0.33 m, 3.75 mL). First, NEt_3_ (167 µL, 1.20 mmol, 0.97 eq.) then acetone (2.75 mL, 37.1 mmol, 30.0 eq.) was added under stirring. After 2 h, all volatiles were removed *in vacuo*. The crude product was purified by flash column chromatography (SiO_2_, *n*-pentane/EtOAc, 2:1) to afford **(*S*)-3b** (200 mg, 0.934 mmol, 75 %) as a yellow solid.

**R*_f_*** (*n*-pentane/EtOAc 2:1) = 0.21.

**^1^H NMR** (400 MHz, CD_2_Cl_2_, ppm): δ = 8.32 (s, 1H, *N*H^3^), 7.31 (m, 4H, H^11, 12, 14, 15^), 7.24 (m, 1H, H^13^), 4.14 (dd, *J* = 8.3, 4.2 Hz, 1H, H^1^), 3.49 (dd, *J*= 14.3, 4.2 Hz, 1H, H^5a^), 2.96 (dd, *J* = 14.3, 8.2 Hz, 1H, H^5b^), 1.91 (br. s, 1H, *N*H^6^), 1.36 (s, 3H, H^9^), 1.34 (s, 3H, H^10^).

**^13^C NMR** (101 MHz, CD_2_Cl_2_, ppm): δ = 204.4 (1C, C^2^), 138.2 (1C, C^7^), 129.8 (2C, C^11, 15^), 128.9 (2C, C^12, 14^), 127.1 (1C, C^13^), 80.0 (1C, C^8^), 71.4 (1C, C^1^), 39.9 (1C, C^5^), 28.7 (1C, C^10^), 28.0 (1C, C^9^).

**HRMS** (ESI): found: [*M*+H]^+^ 221.1106, C_12_H_17_N_2_S^+^ requires 221.1107.

**HPLC** (IC, *n*-hexane/*i*-PrOH 80:20): *t*_R_ = 5.0 min (98.3 % *ee*).


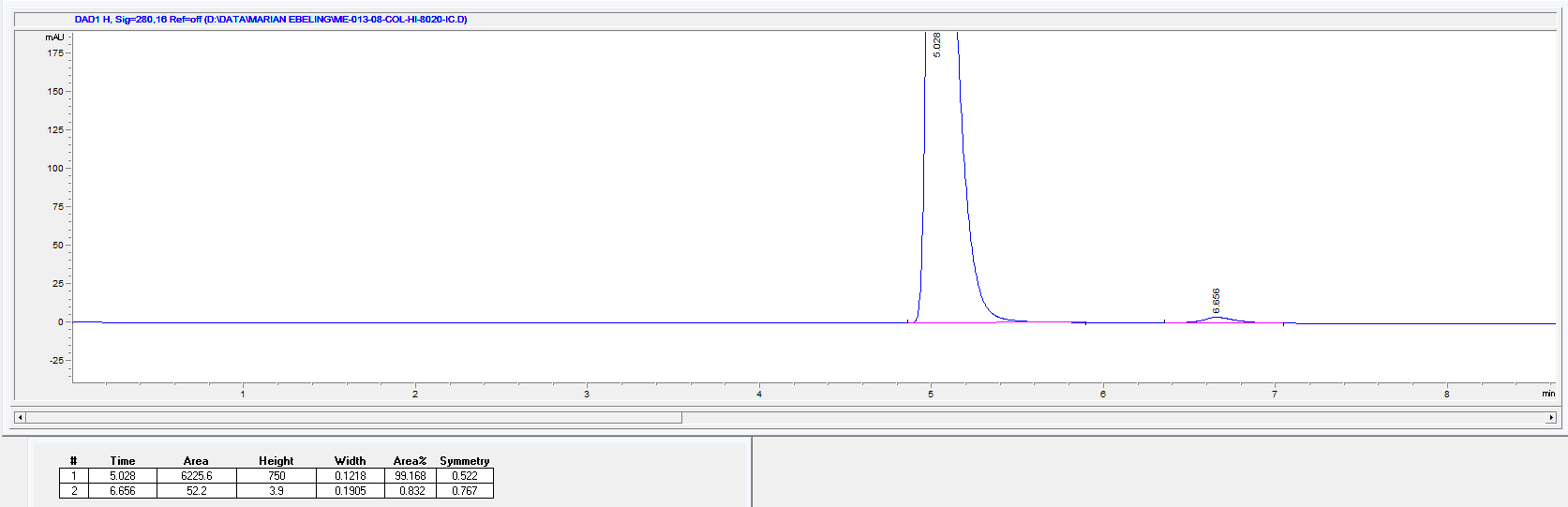


### Synthesis of (*R*)-5-benzyl-2,2-dimethylimidazolidine-4-thione **(*R*)-3b**

**(*R*)-7a** (895 mg, 4.13 mmol, 1.00 eq.) and *p*TsOH (8.0 mg, 41.3 µmol, 0.01 eq.) were dissolved in MeOH (0.33 m, 12.5 mL). First, NEt_3_ (556 µL, 4.00 mmol, 0.97 eq.) then acetone (9.18 mL, 124 mmol, 30.0 eq.) was added under stirring. After 2 h, all volatiles were removed *in vacuo*. The crude product was purified by flash column chromatography (SiO_2_, *n*-pentane/EtOAc, 2:1) to afford **(*R*)-3b** (622 mg, 2.89 mmol, 70 %) as a yellow solid.

**R*_f_*** (*n*-pentane/EtOAc 2:1) = 0.21.

**^1^H NMR** (600 MHz, CD_2_Cl_2_, ppm): δ = 9.18 (br. s, 1H, *N*H^3^), 7.35 - 7.29 (m, 4H, H^11, 12, 14, 15^), 7.27 - 7.23 (m, 1H, H^13^), 4.15 (dd, *J*= 8.3, 4.2 Hz, 1H, H^1^), 3.49 (dd, *J*= 14.4, 4.3 Hz, 1H, H^5a^), 2.96 (dd, *J*= 14.3, 8.2 Hz, 1H, H^5b^), 1.90 (br. s, 1H, *N*H^6^), 1.36 (s, 3H, H^10^), 1.34 (s, 3H, H^9^).

**^13^C NMR** (150 MHz, CD_2_Cl_2_, ppm): δ = 203.8 (1C, C2), 138.1 (1C, C^7^), 129.7 (2C, C^11, 15^), 128.9 (2C, C^12, 14^), 127.1 (1C, C^13^), 80.3 (1C, C^8^), 71.4 (1C, C^1^), 39.8 (C1, C^5^), 28.5 (1C, C^9^), 27.9 (1C, C^10^).

**HRMS** (ESI): found: [*M*+H]^+^ 221.1106, C_12_H_17_N_2_S^+^ requires 221.1107.

**HPLC** (IC, *n*-hexane/*i*-PrOH 80:20): *t*_R_ = 5.0 min (95.4 %*ee*).


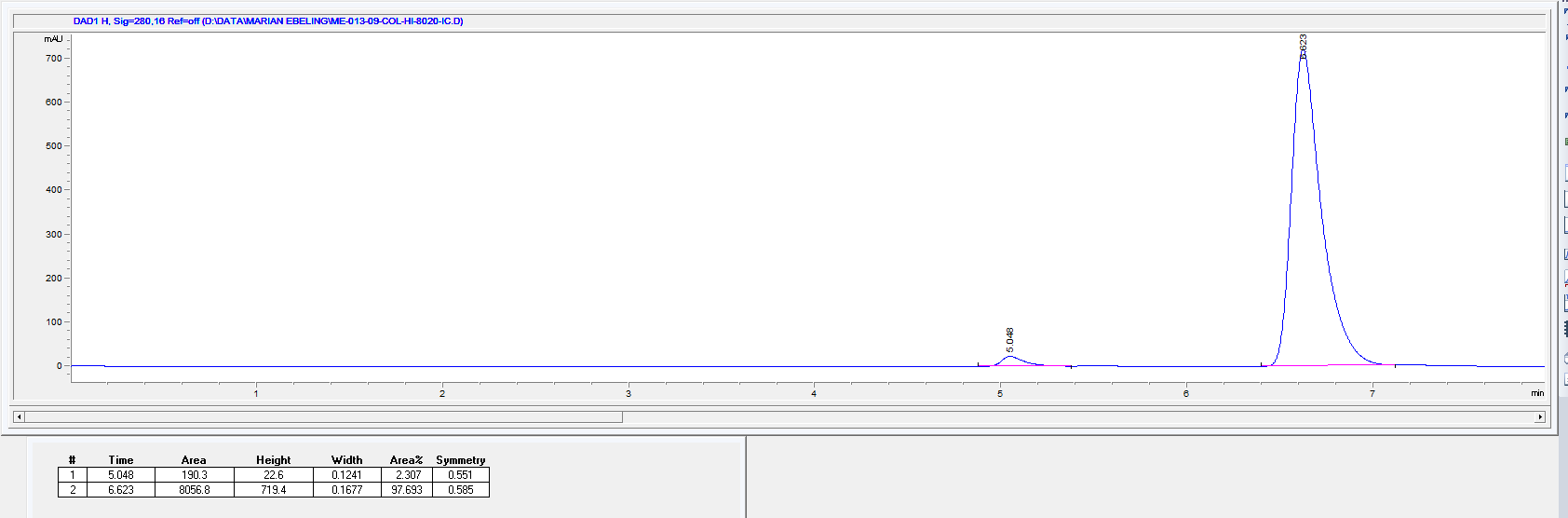


### Synthesis of (2*R*,5*S*)-5-benzyl-2-(*tert*-butyl)imidazolidine-4-thione ***trans*-3c** and (2*S*,5*S*)-5-benzyl-2-(*tert*-butyl)imidazolidine-4-thione ***cis*-3c**

**(*S*)-7a** (852 mg, 3.93 mmol, 1.00 eq.) was neutralized with sat. aq. NaHCO_3_ and extracted with DCM (3 x 100 mL). The organic phase was dried over Na_2_SO_4_ and concentrated *in vacuo*. The free base was dissolved in MeOH (0.4 m, 10 mL) and *p-*TsOH (7.5 mg, 39.3 µmol, 0.01 eq.) was added. Then, pivaldehyde (0.640 mL, 5.90 mmol, 1.50 eq.) was added and the reaction was stirred for 2 h. All volatiles were removed *in vacuo* and the crude product purified by flash column chromatography (puriflash column: PF-15SIHP-F0040, *c*-Hex/EtOAc, 97:3 -> 73:27, over 18 column volumes). ***trans*-3c** (364 mg, 1.47 mmol, 37 %) was obtained as a yellow oil and ***cis*-3c** (128 mg, 0.515 mmol, 13 %) as a white solid.

***trans*-3c:**

**R*_f_*** (*n*-pentane/EtOAc 4:1) = 0.46.

**^1^H NMR** (400 MHz, CD_2_Cl_2_, ppm): δ = 8.41 (br. s, 1H, *N*H^4^), 7.36 – 7.21 (m, 5H, H^13, 14, 15, 16, 17^), 4.28 (d, *J* = 2.3 Hz, 1H, H^3^), 4.18 – 4.12 (m, 1H, H^1^), 3.37 (dd, *J* = 14.2, 4.2 Hz, 1H, H^8a^), 2.95 (dd, *J* = 14.2, 8.1 Hz, 1H, H^8b^), 2.17 (br. s, 1H, *N*H^2^), 0.88 (s, 9H, H^10, 11, 12^).

**^13^C NMR** (101 MHz, CD_2_Cl_2_, ppm): δ = 205.8 (1C, C^5^), 138.0 (1C, C^9^), 129.9 (2C, C^13, 17^), 128.9 (2C, C^14, 16^), 127.2 (1C, C^15^), 85.2 (1C, C^3^), 72.2 (1C, C^1^), 40.7 (1C, C^8^), 36.7 (1C, C^7^), 24.8 (3C, C^10, 11, 12^).

**HRMS** (ESI): found: [*M*+H]^+^ 249.1418, C_14_H_21_N_2_S^+^ requires 249.1420.

**HPLC** (IC, *n*-hexane/*i*-PrOH 80:20): *t*_R_ = 4.6 min (*trans*/*cis* >99).


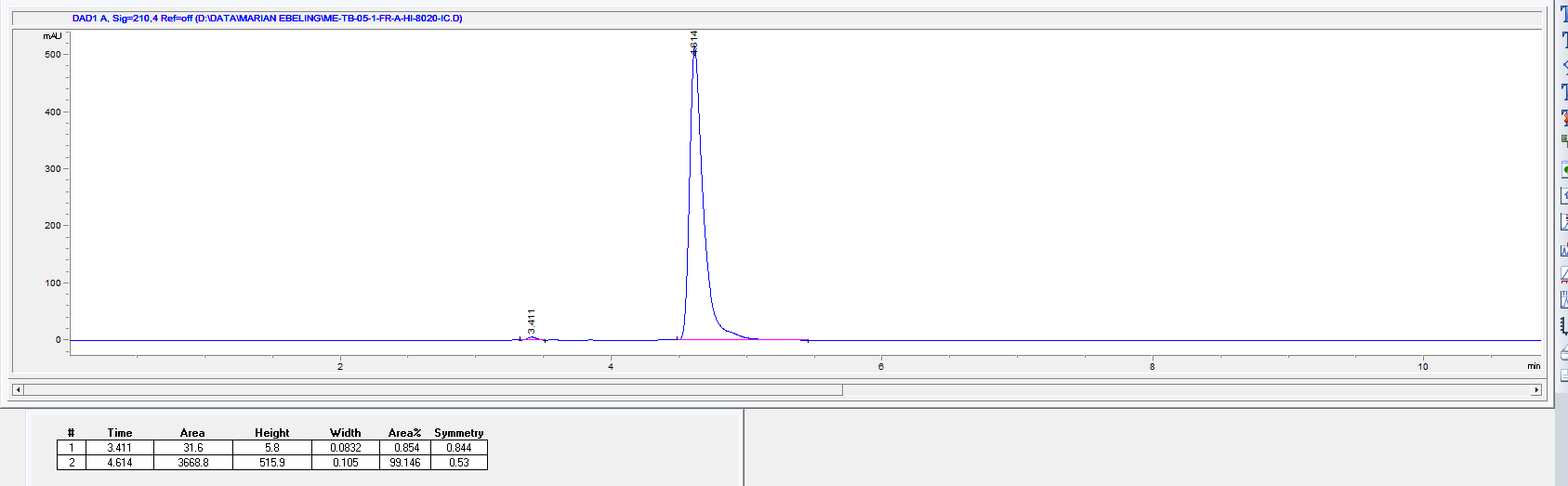


***cis*-3c:**

**R*_f_*** (*n*-pentane/EtOAc 4:1) = 0.26.

**^1^H NMR** (400 MHz, CD_2_Cl_2_, ppm): δ = 8.17 (s, 1H, *N*H^4^), 7.35 - 7.20 (m, 5H, H^13, 14, 15, 16, 17^), 4.41 (d, *J*= 2.3 Hz, 1H, H^3^), 4.07 (dt, *J*= 7.1, 3.1 Hz, 1H, H^1^), 3.44 (dd, *J*= 13.9, 4.0 Hz, 1H, H^8a^), 3.00 (dd, *J*= 13.9, 8.0 Hz, 1H, H^8b^), 1.92 (s, 1H, *N*H^2^), 0.82 (s, 9H, H^10, 11, 12^).

**^13^C NMR** (101 MHz, CD_2_Cl_2_, ppm): δ = 205.8 (1C, C5), 138.4 (1C, C^9^), 130.0 (2C, C^13, 17^), 128.9 (2C, C^14, 16^), 127.0 (1C, C^15^), 84.3 (1C, C^3^), 71.5 (1C, C^1^), 40.0 (1C, C^8^), 34.1 (1C, C^7^), 24.9 (3C, C^10, 11, 12^).

**HRMS** (ESI): found: [*M*+H]^+^ 249.1419, C_14_H_21_N_2_S^+^ requires 249.1420.

**HPLC** (IC, *n*-hexane/*i*-PrOH 80:20): *t*_R_ = 4.9 min (*trans*/*cis* 98:02).


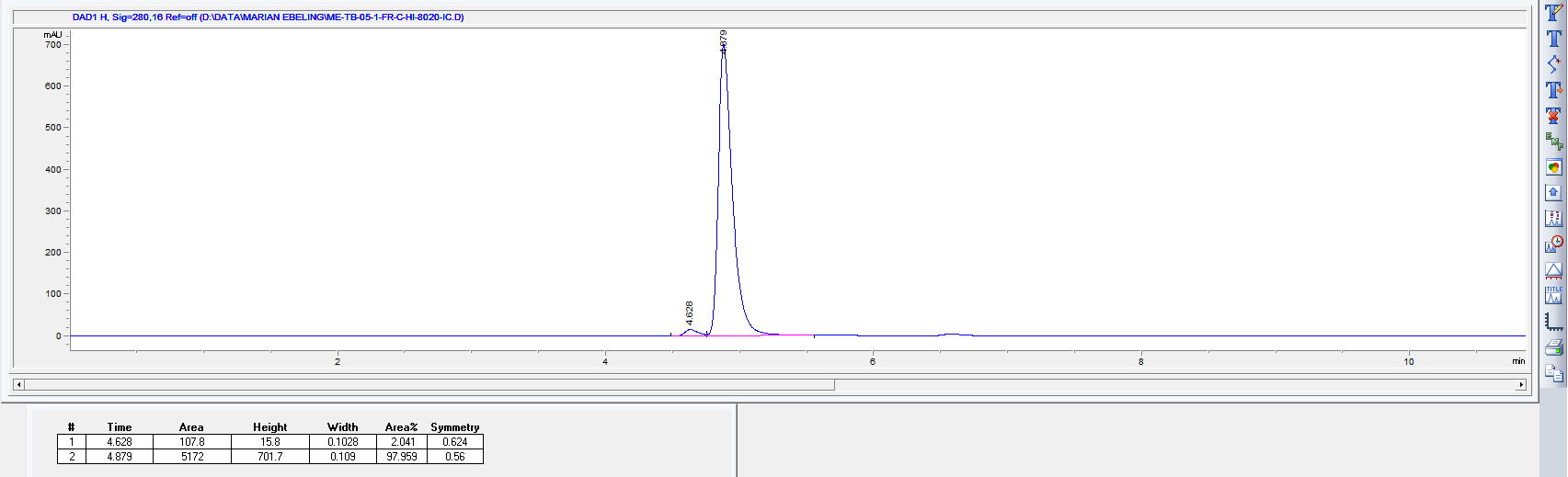


**Crystal structure** of ***cis*-3c**


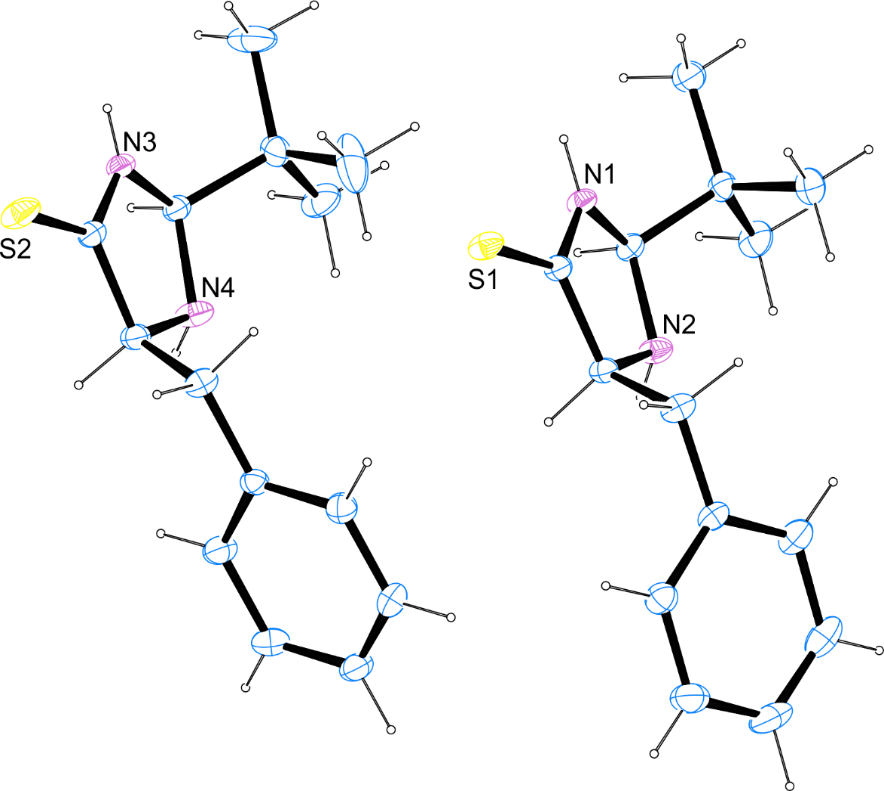


### Synthesis of (*S*)-5-benzyl-2,2-dimethyl-4-(methylthio)-2,5-dihydro-1*H*-imidazole **8**

**(*S*)-3b** (241 mg, 1.10 mmol, 1.00 eq.) and DBU (0.072 mL, 0.484 mmol, 1.05 eq.) were dissolved in dry MeOH (0.25 m, 2 mL) under argon. Then, MeI (0.030 mL, 0.484 mmol, 1.05 eq.) was added and the reaction mixture stirred for 4 h under exclusion of light, until no more educt was detected by TLC. All volatiles were removed under high vacuum. The crude product was purified by flash column chromatography (SiO_2_, *n*‑pentane/EtOAc, 1:1) to obtain **8** (96.9 mg, 0.413 mmol, 90 %) as a yellow oil.

**R*_f_*** (*n*-pentane/EtOAc 1:1) = 0.26.

**^1^H NMR** (400 MHz, CD_2_Cl_2_, ppm): δ = 7.33 - 7.19 (m, 5H, H^1, 2, 3, 4, 5^), 4.22 (dd, *J*= 8.4, 4.5 Hz, 1H, H^10^), 3.10 (dd, *J*= 14.1, 4.5 Hz, 1H, H^7a^), 2.74 (dd, *J*= 14.1, 8.4 Hz, 1H, H^7b^), 2.46 (s, 3H, H^16^), 2.08 (br. s, 1H, *N*H^9^), 1.27 (s, 3H, H^14^), 1.23 (s, 3H, H^15^).

**^13^C NMR** (101 MHz, CD_2_Cl_2_, ppm): δ = 171.1 (1C, C^11^), 138.3 (1C, C^6^), 129.7 (2C, C^2, 4^), 128.8 (2C, C^1, 5^), 126.9 (1C, C^3^), 89.8 (1C, C^13^), 70.1 (1C, C^10^), 40.1 (1C, C^7^), 30.0 (1C, C^15^), 28.6 (1C, C^14^), 14.1 (1C, C^16^).

**HRMS** (ESI): found: [*M*+H]^+^ 235.1263, C_13_H_19_N_2_S^+^ requires 235.1263.

**HPLC** (IC, *n*-hexane/*i*-PrOH 80:20): *t*_R_ = 4.4 min (98.6 % *ee*).


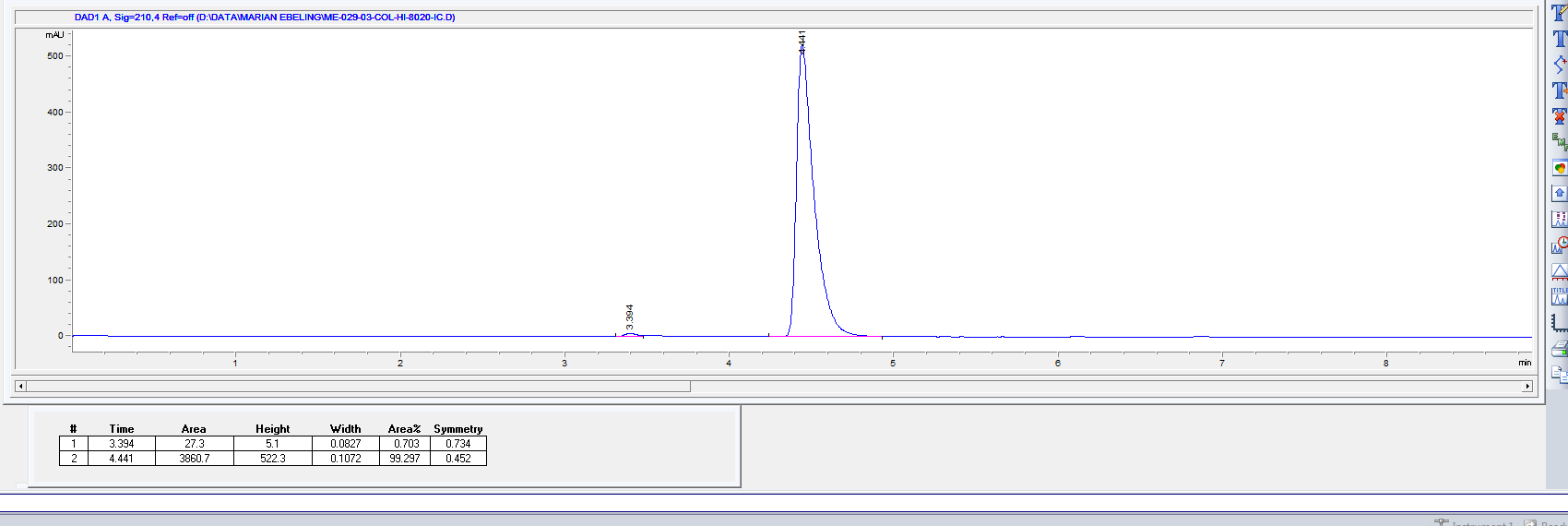


### Synthesis of (*S*)-2-((5-benzyl-2,2-dimethyl-2,5-dihydro-1*H*-imidazol-4-yl)thio)acetonitrile **9**

**(*S*)-3b** (471 mg, 2.14 mmol, 1.00 eq.) and DBU (0.320 mL, 2.14 mmol, 1.00 eq.) were dissolved in dry MeOH (0.25 m, 8.6 mL) under argon. Then, bromoacetonitrile (0.150 mL, 2.14 mmol, 1.00 eq.) was added and the reaction mixture stirred for 4 h under exclusion of light. All volatiles were removed under high vacuum. The crude product was purified by flash column chromatography (SiO_2_, *n*-pentane/EtOAc, 1:1) to obtain **9** (445 mg, 1.71 mmol, 80 %) as an orange oil.

**R*_f_*** (*n*-pentane/EtOAc 1:1) = 0.3.

**^1^H NMR** (400 MHz, CD_2_Cl_2_, ppm): δ = 7.35 - 7.21 (m, 5H, H^14, 15, 16, 17, 18^), 4.30 (dd, *J*= 8.1, 5.1 Hz, 1H, H^1^), 3.90 (d, *J*= 16.5 Hz, 1H, H^11a^), 3.85 (d, *J*= 16.5 Hz, 1H, H^11b^), 3.03 (dd, *J*= 14.1, 5.1 Hz, 1H, H^7a^), 2.81 (dd, *J*= 14.1, 8.0 Hz, 1H, H^7b^), 1.83 (br. s, 1H, *N*H^2^), 1.31 (s, 3H, H^9^), 1.28 (s, 3H, H^10^).

**^13^C NMR** (101 MHz, CD_2_Cl_2_, ppm): δ = 167.2 (1C, C^5^), 137.6 (1C, C^8^), 129.7 (2C, C^14, 18^), 128.9 (2C, C^15, 17^), 127.2 (1C, C^16^), 116.7 (1C, C^12^), 90.4 (1C, C^3^), 69.9 (1C, C^1^), 39.9 (1C, C^7^), 29.8 (1C, C^10^), 28.5 (1C, C^9^), 16.9 (1C, C^11^).

**HRMS** (ESI): found: [*M*+H]^+^ 260.1212, C_14_H_18_N_3_S^+^ requires 260.1216.

**HPLC** (IC, *n*-hexane/*i*-PrOH 80:20): *t*_R_ = 7.4 min (98.8 % *ee*).


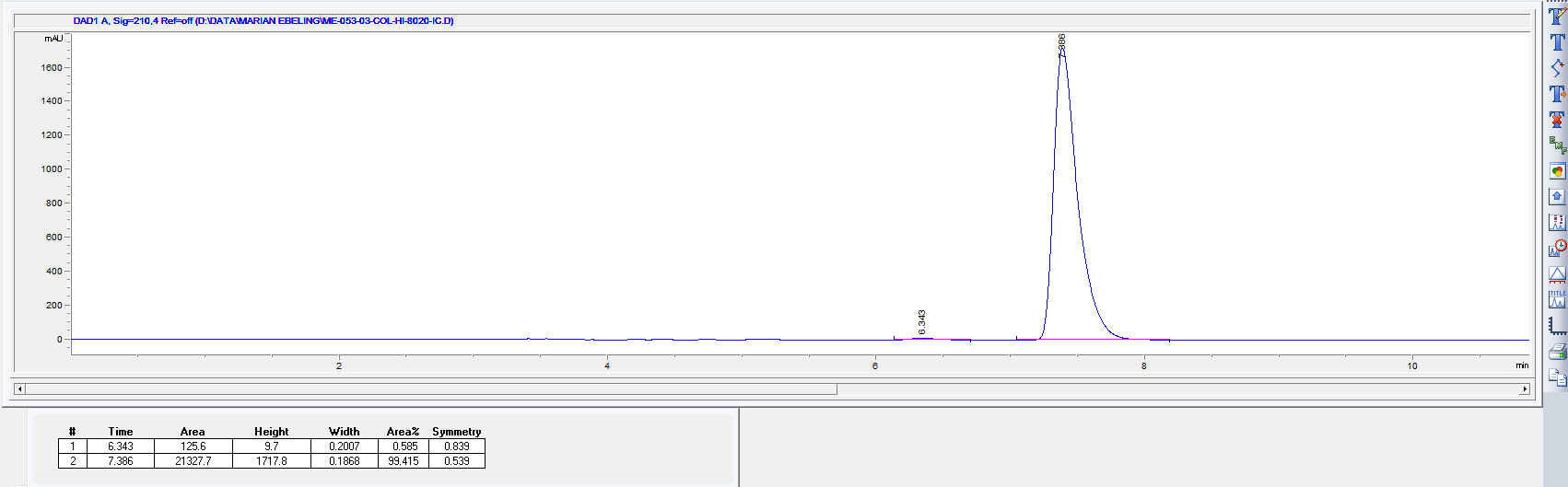


## Diels-Alder reactions

### Preparation of cyclopenta-1,3-diene from dicyclopentadiene

A flask filled with dicyclopentadiene and a catalytic amount of iron turnings was connected via a vigreux column to a short path distillation bridge. The system was flushed with N_2_ and the flask was heated to 200 °C in a sand bath. The collection schlenk-flask was cooled with dry ice (−78 °C) and covered in aluminium foil to prevent dimerization. Until needed, cyclopenta-1,3-diene was stored under argon at −20 °C for up to 3 d.

**^1^H NMR** (400 MHz, CDCl_3_, ppm): δ = 6.60 (ddd, *J*= 5.7, 2.9, 1.6 Hz, 2H, H^1,4/2,3^), 6.49 (dtd, *J*= 5.2, 1.6, 1.0 Hz, 2H, H^1,4/2,3^), 3.01 (td, *J*= 1.2, 0.8 Hz, 2H, H^5^).

**^13^C NMR** (101 MHz, CDCl_3_, ppm): δ = 133.3 (2C, C^2,3/1,4^), 132.4 (2C, C^2,3/1,4^), 41.8 (1C, C^5^).

### Preparation of racemic aldehydes ***endo*-2** and ***exo*-2**

The catalyst **(*rac*)-1** (25.2 mg, 99.3 μmol, 0.05 eq.) was dissolved in MeOH/H_2_O (95:05, 1 m, 2 mL) and *trans*-cinnamaldehyde (250 μL, 1.99 mmol, 1.00 eq.) was added under stirring. After 2 min, cyclopenta-1,3-diene (500 μL, 5.96 mmol, 3.00 eq.) was added and the vial was sealed. After 21 h the reaction was partitioned between Et_2_O (20 mL/mmol) and water (10 mL/mmol). The aqueous phase was discarded and the organic phase was washed with aq. sat. NaCl (20 mL). The organic phase was dried over Na_2_SO_4_ and concentrated *in vacuo*. The crude product was diluted in DCM (4 mL) and water (2 mL) was added. Then, TFA (2 mL) was added slowly and the suspension was stirred for 2 h. After neutralization with aq. sat. NaHCO_3_, the aqueous phase was extracted with Et_2_O (3 x 20 mL), dried over Na_2_SO_4_ and concentrated *in vacuo*. The crude product was purified by automated flash column chromatography (puriflash column: PF-15SIHP-F0040, *n‑*pentane/Et_2_O, 97:03, over 14 column volumes) to yield **(*rac*)-*endo*-2** and **(*rac*)-*exo*-2** (1:1.45) as a colorless oil.

***endo*-2:**

**^1^H NMR** (400 MHz, CD_2_Cl_2_, ppm): δ = 9.60 (d, *J*= 2.2 Hz, 1H), 7.37 - 7.09 (m, 5H), 6.42 (dd, *J*= 5.7, 3.2 Hz, 1H), 6.17 (dd, *J*= 5.7, 2.8 Hz, 1H), 3.34 (dddt, *J*= 4.2, 3.3, 2.4, 1.1 Hz, 1H), 3.13 (dt, *J*= 3.4, 1.7 Hz, 1H), 3.09 (dd, *J*= 5.0, 1.7 Hz, 1H), 2.99 (ddd, *J*= 4.9, 3.5, 2.2 Hz, 1H), 1.85 - 1.78 (m, 1H), 1.65 - 1.59 (m, 1H).

**^13^C NMR** (101 MHz, CD_2_Cl_2_, ppm): δ = 203.7 (1C), 143.7 (1C), 139.4 (1C), 133.9 (1C), 128.7 (2C), 127.5 (2C), 126.3 (1C), 61.0 (1C), 48.6 (1C), 47.3 (1C), 45.8 (1C), 45.3 (1C).

***exo*-2:**

**^1^H NMR** (400 MHz, CD_2_Cl_2_, ppm): δ = 9.93 (d, *J*= 2.1 Hz, 1H), 7.37 - 7.09 (m, 5H), 6.34 (td, *J*= 3.7, 1.6 Hz, 1H), 6.11 - 6.04 (m, 1H), 3.73 (dd, *J*= 5.4, 3.3 Hz, 1H), 3.22 (tq, *J*= 4.0, 1.7 Hz, 2H), 2.60 (dt, *J*= 5.4, 1.9 Hz, 1H), 1.65 - 1.59 (m, 1H), 1.56 (dq, *J*= 8.8, 1.7 Hz, 1H).

**^13^C NMR** (101 MHz, CD_2_Cl_2_, ppm): δ = 203.0 (1C), 142.7 (1C), 136.7 (1C), 136.4 (1C), 128.3 (2C), 128.0 (2C), 126.5 (1C), 59.6 (1C), 48.5 (1C), 47.7 (1C), 45.6 (1C), 45.5 (1C).

**GC-MS** (EI): [*M*]^+^**^∙^** 198.20, C_14_H_14_O^+^**^∙^** requires 198.10; [*M*-C_5_H_7_**^∙^**]^+^ 131.02, C_9_H_7_O^+^ requires 131.05; [*M*-C_6_H_7_O**^∙^**]^+^ 103.08, C_8_H_7_^+^ requires 103.05; [*M*-C_8_H_9_O**^∙^**]^+^ 77.03, C_6_H_5_^+^ requires 77.04; [*M*-C_9_H_8_O**^∙^**]^+^ 66.06, C_5_H_6_^+^ requires 66.05.

**GC-FID** (88 °C, 100 kPa, Chirasil-β-dex, l = 10 m): (2*R*)-*endo*-**2** (99.5 min), (2*R*)-*exo*-**2** (102.9 min), (2*S*)-*endo*-**2** (114.7 min), (2*S*)-*exo*-**2** (130.6 min).

The NMR spectra were consistent with values reported in literature.^[41]^

The procedure was based on a literature synthesis.^[4]^

### Organocatalyzed Diels-Alder Reaction of cyclopenta-1,3-diene and *trans*-cinnamaldehyde – general procedures

General Procedure – GP1

The catalyst (0.05 eq.) was dissolved in MeOH/H_2_O (95:05, 1 m) and *trans*-cinnamaldehyde (1.00 eq.) was added under stirring. After 2 min, cyclopenta-1,3-diene (3.00 eq.) was added and the vial was sealed. After 21 h, *n*-heptadecane (155 μL/mmol, 0.50 eq.) was added and the reaction was partitioned between Et_2_O (20 mL/mmol) and water (10 mL/mmol). The aqueous phase was discarded and the organic phase was washed with aq. sat. NaCl (10 mL/mmol). The organic phase was dried over Na_2_SO_4_ and concentrated *in vacuo*. The crude product was diluted in DCM (2 mL/mmol) and water (1 mL/mmol) was added. Then, TFA (1 mL/mmol) was added slowly and the suspension was stirred for 2 h. After neutralization with aq. sat. NaHCO_3_, the aqueous phase was extracted with Et_2_O (3 x 10 mL/mmol), dried over Na_2_SO_4_ and concentrated *in vacuo*. The sample of the crude product **2** was objected to GC-analysis.

General Procedure – GP2

The free base catalyst (0.05 eq.) was dissolved in MeOH/1 m HCl (95:05, 1 m) and *trans*-cinnamaldehyde (1.00 eq.) was added under stirring. After 2 min, cyclopenta-1,3-diene (3.00 eq.) was added and the vial was sealed. After 21 h, *n*-heptadecane (155 μL/mmol, 0.50 eq.) was added and the reaction was partitioned between Et_2_O (20 mL/mmol) and water (10 mL/mmol). The aqueous phase was discarded and the organic phase was washed with aq. sat. NaCl (10 mL/mmol). The organic phase was dried over Na_2_SO_4_ and concentrated *in vacuo*. The crude product was diluted in DCM (2 mL/mmol) and water (1 mL/mmol) was added. Then, TFA (1 mL/mmol) was added slowly and the suspension was stirred for 2 h. After neutralization with aq. sat. NaHCO_3_, the aqueous phase was extracted with Et_2_O (3 x 10 mL/mmol), dried over Na_2_SO_4_ and concentrated *in vacuo*. The sample of the crude product **2** was subjected to GC-analysis.

**Figure S1** Exemplary GC-Analysis of the crude Diels-Alder products with n-heptadecane (C17) at 80.1 min.

### GC – linear fit

For the determination of the yield, *n*-heptadecane (C17) was chosen as an internal standard. To calculate the correction factor for the GC measurements, different Diels-Alder-product **2** / *n*‑heptadecane ratios were prepared and analysed by GC. Here, a racemic mixture of **2** was used. An isothermal method at 88 °C with a Chirasil-β-Dex column (l = 10 m, ID = 250 μm, FT = 500 nm) was used for all measurements. The tested ratios were listed in **Table S1** and the resulting linear fit of product **2**/C17 ratio against the molar ratio of product **2**/C17 in **Figure S2**. The linear fit gave following equation:

molar ratio (product **2**/C17) = 1.53682 * GC ratio (**2**/C17) – 0.07476

**Table S1** Molar ratios of Diels-Alder product **2** to internal standard C17 and resulting ratios by integration of GC peaks.

| C17  [μL] | C17 [mmol] | **2** (0.4 m in EtOAc)  [μL] | **2** [mmol] | molar ratio  **2**/C17 | measured GC ratio **2**/C17 | corresponding theor. yield [%] |
| --- | --- | --- | --- | --- | --- | --- |
| 25.0 | 0.08078 | 480 | 0.192 | 2.376945174 | 1.53503638 | 118.8 |
| 25.0 | 0.08078 | 440 | 0.176 | 2.178866409 | 1.48008966 | 108.9 |
| 25.0 | 0.08078 | 420 | 0.168 | 2.079827027 | 1.36430291 | 104.0 |
| 25.0 | 0.08078 | 400 | 0.160 | 1.980787645 | 1.32877788 | 99.0 |
| 25.0 | 0.08078 | 360 | 0.144 | 1.78270888 | 1.23389098 | 89.1 |
| 25.0 | 0.08078 | 340 | 0.136 | 1.683669498 | 1.18083943 | 84.2 |
| 25.0 | 0.08078 | 320 | 0.128 | 1.584630116 | 1.07149241 | 79.2 |
| 25.0 | 0.08078 | 280 | 0.112 | 1.386551351 | 0.96982992 | 69.3 |
| 25.0 | 0.08078 | 240 | 0.096 | 1.188472587 | 0.88851927 | 59.4 |
| 25.0 | 0.08078 | 200 | 0.080 | 0.990393822 | 0.70371208 | 49.5 |
| 25.0 | 0.08078 | 160 | 0.064 | 0.792315058 | 0.56420471 | 39.6 |
| 25.0 | 0.08078 | 120 | 0.048 | 0.594236293 | 0.4226609 | 29.7 |
| 25.0 | 0.08078 | 80 | 0.032 | 0.396157529 | 0.28255698 | 19.8 |
| 25.0 | 0.08078 | 40 | 0.016 | 0.198078764 | 0.15729147 | 9.9 |

**Figure S2** Plot of **Table S1**: molar ratio of product **2**/C17 against GC ratio (product **2**/C17) and the resulting linear fit.

### Screening of catalysts – yield, *exo*:*endo* and *ee*

According to the general procedures (GP1 / GP2), the following catalysts were tested. 310 μL (1.00 mmol) of *n*‑heptadecane (C17) were added to the reaction mixture after 21 h, which correlates to 0.5 equivalents. After the workup, the crude product was analysed by GC and the total integral of all 4 product isomers of **2** was divided by the integral of C17 (**Figure S1**). By using the linear fit of **Figure S2**, the molar amount of the product was determined by calculating the molar ratio of **2**/C17 and then multiplying with the amount of C17 used (1.00 mmol). The yield was calculated by division of the molar amount of product with 1.99 mmol of the inital *trans*-cinnamaldehyde.

Yield [%] = $\frac{\left( 1.54 \cdot GC ratio \left[ \frac{\boldsymbol{Diels-Alder product 2}}{C17} \right]-0.0748 \right) \cdot1.00 mmol}{1.99 mmol}$

Exemplary calculation for **Table S2**, entry 1a:

total GC integral of **2**: 34645046

GC integral C17: 30322956

GC ratio **2**/C17: 1.1425352

yield [mmol] = $(1.54\cdot1.1425352-0.0748) \cdot1.00 mmol = 1.68 mmol$

yield [%] = 1.68 mmol / 1.99 mmol = 84.6 %

**Table S2** Overview of all catalysts tested for Diels-Alder reaction of trans-cinnamaldehyde and cyclopenta-1,3-diene according to GP1 or GP2. All reaction entries were performed thrice (entry a, b, c). The yield was determined by referencing to the internal standard n-heptadecane in the GC-analysis. The exo:endo ratio and all ee were determined by GC.

| entry | catalyst | catalyst*ee* [%] | aldehyde[mmol] | general procedure | GC ratio  **2**/C17 | yield [%] | *exo*:*endo* | *exo ee* [%] | | *endo ee* [%] | |
| --- | --- | --- | --- | --- | --- | --- | --- | --- | --- | --- | --- |
| 1a | ****  **(*S*)-1** | >99 | 1.99 | GP1 | 1.1320688 | 83.8 | 1.33 | 92.4 | (2*S*) | 93.7 | (2*S*) |
| 1b |  |  |  |  | 1.1929592 | 88.5 | 1.32 | 91.6 | (2*S*) | 94.0 | (2*S*) |
| 1c |  |  |  |  | 1.1712568 | 86.8 | 1.31 | 92.0 | (2*S*) | 93.9 | (2*S*) |
| 2a | **(*R*)-1** | >99 | 1.99 | GP1 | 1.1813335 | 87.6 | 1.45 | 93.2 | (2*R*) | 92.7 | (2*R*) |
| 2b |  |  |  |  | 1.2734229 | 94.7 | 1.63 | 92.7 | (2*R*) | 92.2 | (2*R*) |
| 2c |  |  |  |  | 1.2123203 | 90.0 | 1.56 | 93.7 | (2*R*) | 92.3 | (2*R*) |
| 3a | **(*S*)-3a** | 98.8 | 1.99 | GP2 | 1.1193489 | 82.8 | 1.35 | 88.5 | (2*S*) | 90.9 | (2*S*) |
| 3b |  |  |  |  | 1.1455453 | 84.8 | 1.35 | 88.3 | (2*S*) | 90.3 | (2*S*) |
| 3c |  |  |  |  | 1.1686518 | 86.6 | 1.35 | 88.0 | (2*S*) | 89.8 | (2*S*) |
| 4a | **(*S*)-3b** | 98.3 | 1.99 | GP2 | 1.0103136 | 74.4 | 1.30 | 80.4 | (2*S*) | 82.8 | (2*S*) |
| 4b |  |  |  |  | 1.0320286 | 76.1 | 1.35 | 82.6 | (2*S*) | 82.7 | (2*S*) |
| 4c |  |  |  |  | 1.0854614 | 80.2 | 1.35 | 82.5 | (2*S*) | 82.9 | (2*S*) |
| 5a | ****  **(*R*)-3b** | 95.4 | 1.99 | GP2 | 1.0672632 | 78.8 | 1.44 | 79.4 | (2*R*) | 78.3 | (2*R*) |
| 5b |  |  |  |  | 1.0093342 | 74.3 | 1.47 | 76.7 | (2*R*) | 76.9 | (2*R*) |
| 5c |  |  |  |  | 0.9592956 | 70.4 | 1.33 | 78.1 | (2*R*) | 71.4 | (2*R*) |
| 6a | ****  ***trans*-3c** | *cis*/*trans*  1:99 | 1.99 | GP2 | 0.6822566 | 49.0 | 1.95 | 56.4 | (2*S*) | 8.4 | (2*S*) |
| 6b |  |  |  |  | 0.72001 | 51.9 | 2.00 | 56.7 | (2*S*) | 8.0 | (2*S*) |
| 6c |  |  |  |  | 0.712453 | 51.3 | 2.00 | 54.6 | (2*S*) | 1.6 | (2*S*) |
| 7a | ****  ***cis*-3c** | *cis*/*trans*  98:2 | 1.99 | GP2 | 0.7648334 | 55.4 | 2.12 | 55.4 | (2*S*) | 10.7 | (2*S*) |
| 7b |  |  |  |  | 0.8048647 | 58.5 | 2.05 | 55.9 | (2*S*) | 7.5 | (2*S*) |
| 7c |  |  |  |  | 0.7589779 | 54.9 | 2.04 | 57.0 | (2*S*) | 7.2 | (2*S*) |
| 8a | ****  **8** | 98.6 | 1.99 | GP2 | 0.5460185 | 38.5 | 1.28 | 73.7 | (2*S*) | 70.9 | (2*S*) |
| 8b |  |  |  |  | 0.5551711 | 39.2 | 1.32 | 79.0 | (2*S*) | 78.5 | (2*S*) |
| 8c |  |  |  |  | 0.5872071 | 41.7 | 1.32 | 80.1 | (2*S*) | 81.3 | (2*S*) |
| 9a | ****  **9** | 98.8 | 1.99 | GP2 | 0.7739436 | 56.1 | 1.32 | 79.7 | (2*S*) | 82.2 | (2*S*) |
| 9b |  |  |  |  | 0.7746891 | 56.2 | 1.36 | 79.0 | (2*S*) | 80.8 | (2*S*) |
| 9c |  |  |  |  | 0.8148788 | 59.3 | 1.35 | 78.7 | (2*S*) | 80.6 | (2*S*) |

**Table S3** Raw data integrals of Diels-Alder catalyst screening of C17, (2R)-endo-**2**, (2R)-exo-**2**, (2S)-endo-**2** and (2S)-exo-**2.**

| entry | C17 | (2*R*)-*endo*-**2** | (2*R*)-*exo*-**2** | (2*S*)-*endo*-**2** | (2*S*)-*exo*-**2** |
| --- | --- | --- | --- | --- | --- |
| 1a | 30365229.9 | 461914.647 | 749808.654 | 14308893.3 | 18854914 |
| 1b | 20105548.5 | 310245.185 | 576713.951 | 10020217.1 | 13077922.7 |
| 1c | 11802694.3 | 183648.906 | 312638.036 | 5800772.84 | 7526925.74 |
| 2a | 22296956.9 | 10337764.8 | 15082763 | 391984.136 | 527629.589 |
| 2b | 37300507.2 | 17370087.5 | 28353609.6 | 704936.174 | 1070687.09 |
| 2c | 34100859.7 | 15511825.5 | 24416562 | 617493.142 | 795283.988 |
| 3a | 131614957 | 2855046.35 | 4874667.83 | 59756529.6 | 79836818.7 |
| 3b | 90043343.9 | 2126483.62 | 3476877.83 | 41688556.1 | 55856807.7 |
| 3c | 99344768.9 | 2561340.08 | 3958640.76 | 47669474.1 | 61909992.5 |
| 4a | 82421658.3 | 3107710.87 | 4616953.83 | 33105667.8 | 42441388.4 |
| 4b | 49697509.7 | 1880172.94 | 2561745.29 | 19927816.7 | 26919517.4 |
| 4c | 84869397.5 | 3346820.67 | 4633834.25 | 35846740.5 | 48295058.5 |
| 5a | 24696368.2 | 9628662.21 | 13952297.9 | 1173180.81 | 1603385.01 |
| 5b | 15013883 | 5424922.65 | 7970308.06 | 708160.85 | 1050633.82 |
| 5c | 7335130.15 | 2586193.86 | 3579730.36 | 431153.462 | 439480.745 |
| 6a | 14926541.5 | 1580154.68 | 1466170.1 | 1871628.71 | 5265778.07 |
| 6b | 18231220.1 | 2007410.55 | 1898322.62 | 2354152.82 | 6866774.22 |
| 6c | 13202377.4 | 1539974.76 | 1423944.58 | 1589285.76 | 4852867.69 |
| 7a | 119926381 | 13137398.1 | 13904507.5 | 16292442.4 | 48389354.7 |
| 7b | 61397600.7 | 7487318.82 | 7331222.13 | 8708617.47 | 25889600.6 |
| 7c | 51564156.4 | 5981532.72 | 5645129.42 | 6903519.45 | 20605871.8 |
| 8a | 91470023.3 | 3180559.08 | 3694299.97 | 18681925.1 | 24387542.3 |
| 8b | 81535351.5 | 2096393.06 | 2699566.04 | 17403374.8 | 23066734.3 |
| 8c | 64819308.6 | 1533709.26 | 2154356.46 | 14844538.3 | 19529751.8 |
| 9a | 42617088.1 | 1260637.25 | 1905163.83 | 12943205.9 | 16874214.3 |
| 9b | 24104892.3 | 758451.006 | 1132753.38 | 7141364.22 | 9641228.44 |
| 9c | 36425824.4 | 1224838.55 | 1815264.62 | 11402743.2 | 15239786.6 |

## Step-wise cyclization of *trans*-cinnamaldehyde and imidazolidine-4-thione **(*S*)-3b**

### Synthesis of bicycle-acetal **10** and bicycle-aldehyde **11**

**(*S*)-3b** (110 mg, 500 μmol, 1.00 eq.) was dissolved in MeOH/1 m aq. HCl (95:5, 1.25 mL) and *trans*-cinnamaldehyde (125 μL, 1.00 mmol, 2.00 eq.) was added under stirring. The solution was stirred for 4 d, diluted with H_2_O (10 mL) and extracted with EtOAc (3 x 30 mL). The combined organic phases were dried over Na_2_SO_4_ and concentrated *in vacuo*. The crude product was purified by flash column chromatography (puriflash column: PF-15SIHP-F0040, *c-*Hex/EtOAc, 95:05 -> 50:50, over 17 column volumes) to obtain the acetal **10** (103.7 mg, 202 μmol, 40 %, 41 % *ee*) as a yellow solid and the aldehyde **11** (62.8 mg, 135 μmol, 27 %, 76 % *ee*) as a yellow solid.

**R*_f_*** (*c*-Hex/EtOAc 4:1) = 0.47.

**^1^H NMR** (400 MHz, CD_2_Cl_2_, ppm): δ = 8.35 (d, *J*= 24.7 Hz, 1H, H^3^), 7.49 - 7.16 (m, 15H, H^17-31^), 6.74 (dd, *J*= 15.7, 1.4 Hz, 1H, H^11^), 6.53 (dd, *J*= 15.8, 5.5 Hz, 1H, H^10^), 4.38 (ddd, *J*= 8.9, 5.6, 1.4 Hz, 1H, H^9^), 4.11 (d, *J*= 2.6 Hz, 1H, H^16^), 3.34 (s, 3H, H^32^), 3.32 (d, *J*= 12.4 Hz, 1H, H^14^), 3.30 (s, 3H, H^33^), 3.10 - 3.05 (m, 1H, H^13^), 3.07 - 2.99 (m, 1H, H^5a/b^), 2.80 (d, *J*= 13.0 Hz, 1H, H^5a/b^), 1.47 (s, 3H, H^7^), 0.49 (s, 3H, H^8^).

**^13^C NMR** (101 MHz, CD_2_Cl_2_, ppm): δ = 202.0 (1C, C^4^), 138.1 (1C, C^12^), 138.0 (1C, C^6^), 136.8 (1C, C^15^), 134.8 (1C, C^10^), 132.2 (C^Ph^), 129.0 (C^Ph^), 128.9 (1C, C^11^), 127.9 (C^Ph^), 127.9 (C^Ph^), 127.8 (C^Ph^), 127.6 (C^Ph^), 127.5 (C^Ph^), 126.8 (C^Ph^), 126.6 (C^Ph^), 106.6 (1C, C^16^), 86.4 (1C, C^2^), 81.2 (1C, C^1^), 59.4 (1C, C^9^), 58.1 (1C, C^14^), 57.8 (1C, C^32^), 56.2 (1C, C^33^), 54.3 (1C, C^13^), 43.0 (1C, C^5^), 31.3 (1C, C^8^), 26.7 (1C, C^7^).

**HRMS** (ESI): found: [*M*+H]^+^ 513.2573, C_32_H_37_N_2_O_2_S^+^ requires 513.2570.

**HPLC** (IC, *n*-hexane/*i*-PrOH 80:20): *t*_R_ = 4.1 min (major), 7.5 min (minor).


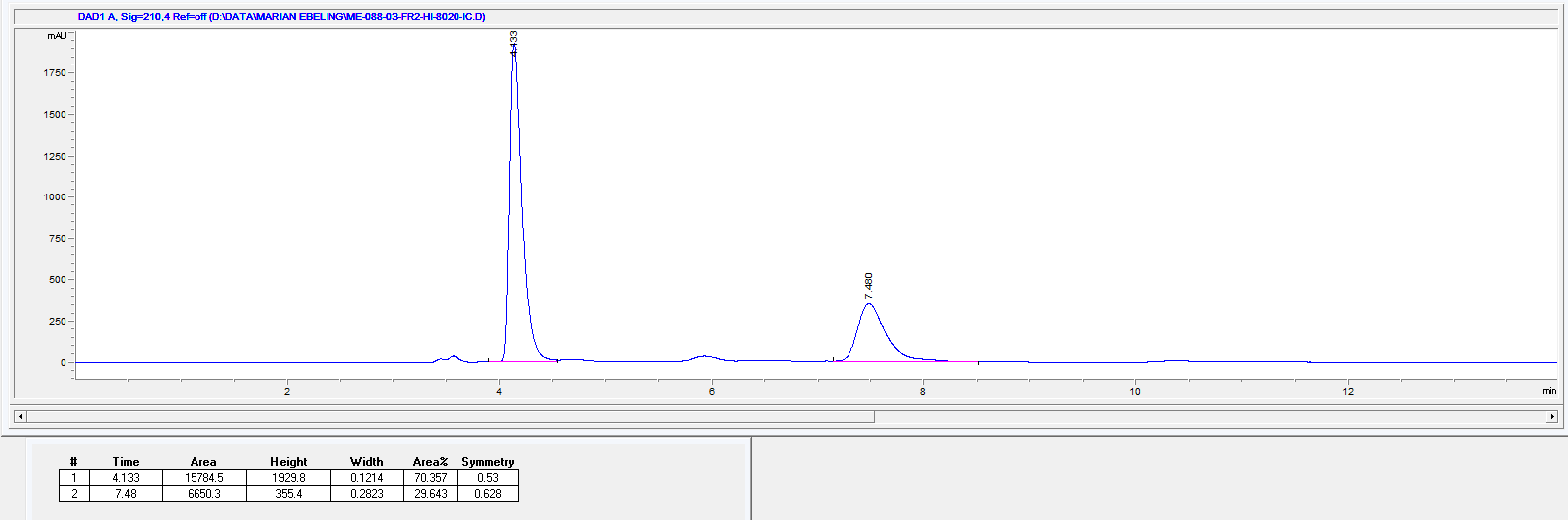

**R*_f_*** (*c*-Hex/EtOAc 4:1) = 0.31.

**^1^H NMR** (400 MHz, CD_2_Cl_2_, ppm): δ = 9.61 (d, *J*= 2.9 Hz, 1H, H^16^), 8.80 (d, *J*= 39.5 Hz, 1H, H^3^), 7.59 - 7.19 (m, 15H, H^17-31^), 6.69 (d, *J*= 15.8 Hz, 1H, H^11^), 6.36 (dd, *J*= 15.8, 6.8 Hz, 1H, H^10^), 4.45 (ddd, *J*= 9.4, 6.8, 1.1 Hz, 1H, H^9^), 3.76 (ddd, *J*= 12.5, 9.3, 2.9 Hz, 1H, H^13^), 3.64 (d, *J*= 12.8 Hz, 1H, H^14^), 3.05 (dd, *J*= 13.1, 1.6 Hz, 1H, H^5a^), 2.82 (dd, *J*= 13.1, 1.6 Hz, 1H, H^5b^), 1.52 (s, 3H, H^7^), 0.45 (s, 3H, H^8^).

**^13^C NMR** (101 MHz, CD_2_Cl_2_, ppm): δ = 201.0 (1C, C^4^), 200.3 (1C, C^16^), 137.3 (1C, C^6^), 136.8 (1C, C^12^), 134.6 (1C, C^15^), 132.1 (C^Ph^), 132.1 (C^Ph^), 131.4 (1C, C^10^), 131.3 (C^Ph^), 131.2 (1C, C^11^), 129.0 (C^Ph^), 128.2 (C^Ph^), 128.1 (C^Ph^), 128.0 (C^Ph^), 127.0 (C^Ph^), 126.9 (C^Ph^), 86.6 (1C, C^1^), 80.9 (1C, C^2^), 62.5 (1C, C^13^), 60.4 (1C, C^9^), 57.2 (1C, C^14^), 43.3 (1C, C^5^), 31.3 (1C, C^8^), 26.6 (1C, C^7^).

**HRMS** (ESI): found: [*M*+H]^+^ 467.2149, C_30_H_31_N_2_OS^+^ requires 467.2152.

**HPLC** (IC, *n*-hexane/*i*-PrOH 80:20): *t*_R_ = 5.8 min (major), 8.9 min (minor).


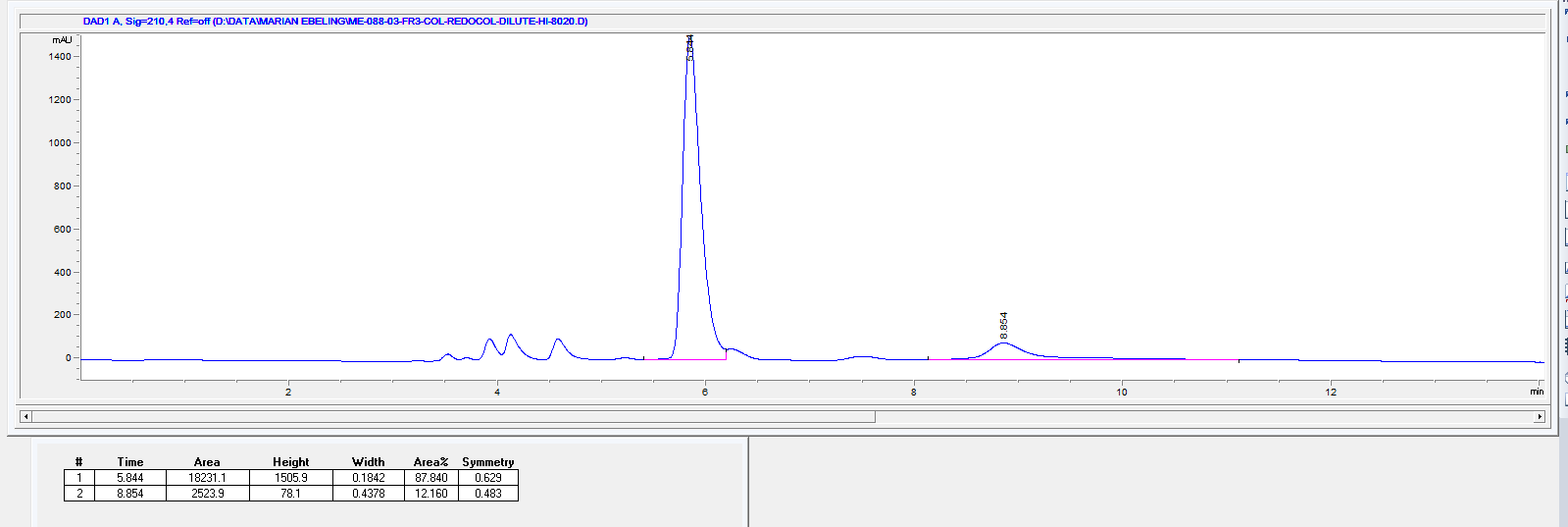


**Crystal structure of 11**:


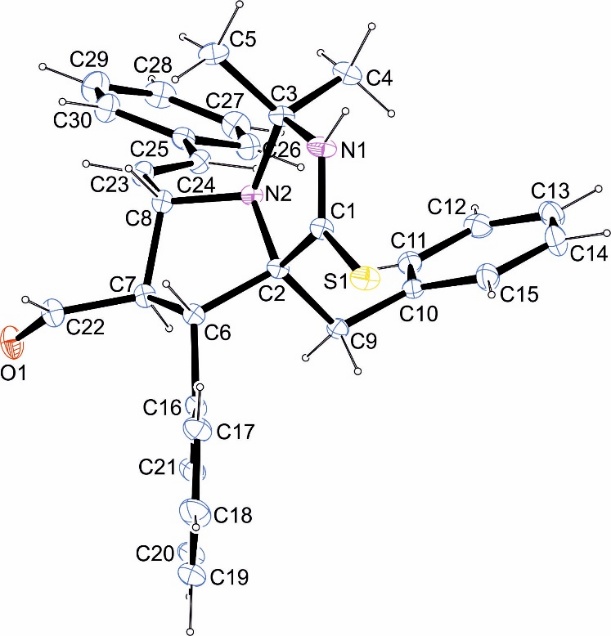


**Figure S3** Crystal structure of aldehyde **11**. Compound **11** and its enantiomer **ent-11** cocrystallize as a racemate. Only component **11** is shown above.

### Optimised synthesis of bicyclic aldehyde **11**:

**(*S*)-3b** (106 mg, 482 μmol, 1.00 eq.) was dissolved in MeCN/1 m aq. HCl (95:5, 0.4 m, 1.14 mL) and *trans*-cinnamaldehyde (121 μL, 1.00 mmol, 2.00 eq.) was added under stirring. The solution was stirred for 1 d, diluted with H_2_O (10 mL) and extracted with EtOAc (3 x 30 mL). The combined organic phases were dried over Na_2_SO_4_ and concentrated *in vacuo*. The crude product was purified by flash column chromatography (puriflash column: PF-15SIHP-F0040, *c-*Hex/EtOAc, 98:02 -> 75:25, over 17 column volumes) to obtain the aldehyde **11** (147 mg, 315 μmol, 65 %, 65 % *ee*) as a yellow solid.


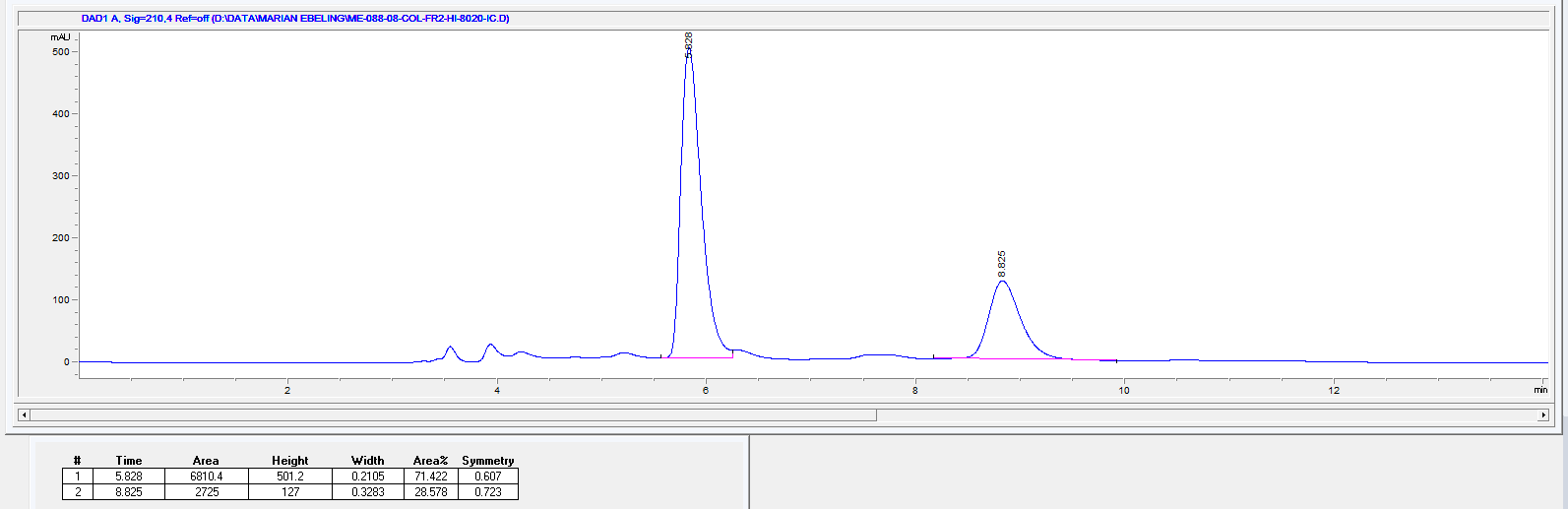


### Acetal formation of compound **10** from aldehyde **11**

To show the selective protection of the aldehyde **11** to the acetal **10**, **11** was dissolved in MeOH and a catalytic amount aq. conc. HCl was added. The solution was stirred for 6 h at 60 °C, all volatiles were evaporated under high vacuum and the crude product dissolved in CD_2_Cl_2_ for ^1^H-NMR analysis (**Figure S4**). The crude acetal protection product (no. 1) showed matching signals with the reference spectrum of the acetal **10** (no. 2). The aldehyde signal of **11** (no. 3) is missing in the product.

**Figure S4** Stacked NMR spectra of the acetal protection of the aldehyde **11** (no. 3) to the acetal **10**. Crude product signals (no. 1) match with the reference spectrum of **10** (no. 2).

### Test of reactivity of MacMillan catalyst **(*S*)-1** towards bicycle formation

For a better understanding of the impact of thionation, **(*S*)-1** was tested for bicycle formation as well. Here, the MacMillan catalyst **(*S*)-1** (1 eq.) was dissolved in MeOH/H_2_O (95:05, 0.4 m) and stirred with cinnamaldehyde (2 eq.). After 7 d, a TLC of the reaction mixture still showed no new product formation, but only the two reactants (In comparison: **(*S*)-3b** showed full conversion of **(*S*)-3b** *via* TLC after only 1 d). Only HRMS (ESI) detected small amounts of the proposed bicyclic structure, whereas the catalyst and the catalyst-aldehyde iminium ion were the predominantly found masses (**Figure S5**).

bicyclic aldehyde:

**HRMS** (ESI): found: [*M*+H]^+^ 465.2531, C_31_H_33_N_2_O_2_^+^ requires 465.2537.

bicyclic acetal:

**HRMS** (ESI): found: [*M*+H]^+^ 511.2953, C_33_H_39_N_2_O_3_^+^ requires 511.2955.

catalyst **(*S*)-1**:

**HRMS** (ESI): found: [*M*+H]^+^ 219.1491, C_13_H_19_N_2_O^+^ requires 219.1492.

catalyst-aldehyde iminium ion:

**HRMS** (ESI): found: [*M*]^+^ 333.1961, C_22_H_25_N_2_O^+^ requires 333.1961.


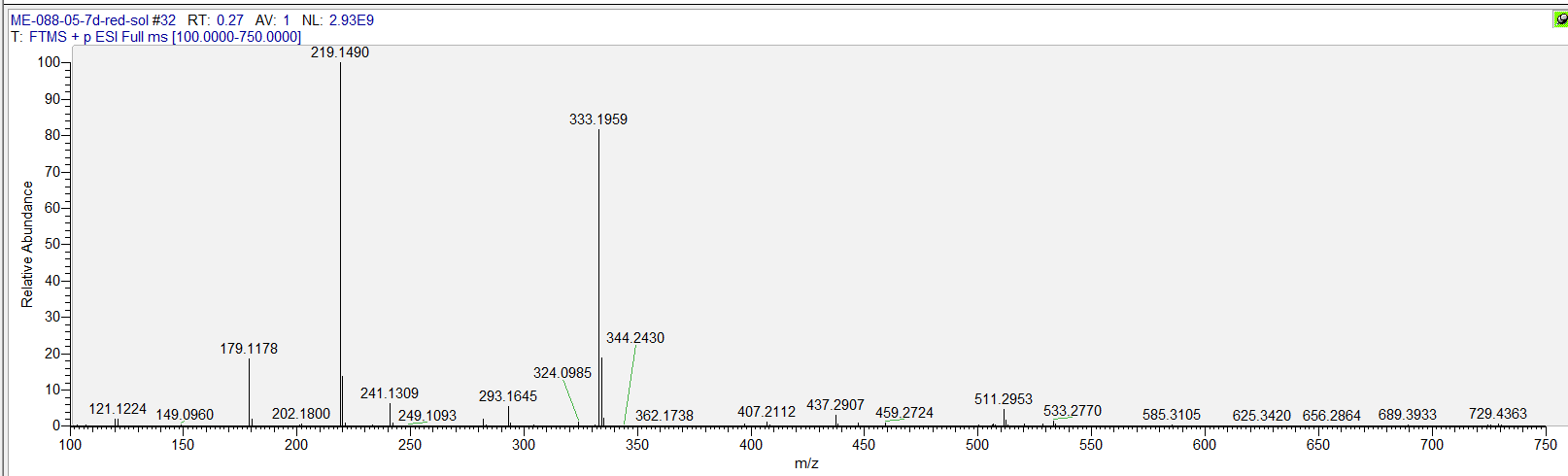


**Figure S5** HRMS spectrum of a reaction mixture of **(S)-1** and trans-cinnamaldehyde in MeOH/H_2_O (95:05, 0.4 M) after 7 d.

### Synthesis of bicycle-aldehyde **14** from crotonaldehyde

Applying the conditions used for the synthesis of **11**, degradation of the aldehyde and unconsumed **(*S*)-3b** can be observed after 1 d. The synthesis of **14** was modified by the omission of aqueous acid and the use of an excess of *trans*-crotonaldehyde, as well as an extended reaction time of 2 d.

**(*S*)-3b** (55.0 mg, 250 μmol, 1.00 eq.) was dissolved in MeCN (625 μL) and *trans*-crotonaldehyde (41 μL, 1.00 mmol, 2.00 eq.) was added under stirring. After 1 d, more *trans*-crotonaldehyde (410 μL, 10.0 mmol, 20.0 eq.) was added. After 2 d of total reaction time, all volatiles were removed under high vacuum and the crude product was directly purified by flash column chromatography (puriflash column: PF-15SIHP-F0040, *c‑*Hex/EtOAc, 95:05 -> 40:60, over 12 column volumes) to obtain the aldehydes ***exo*-14** (19.3 mg, 56.5 μmol, 22.6 %) and ***endo*-14** (11.3 mg, 33.0 μmol, 13.2 %) as a yellow oil.

***exo*-14**:

**^1^H NMR** (400 MHz, CD_2_Cl_2_, ppm): δ = 9.76 (d, *J* = 2.3 Hz, 1H, H^12^), 8.31 (s, 1H, H^3^), 7.34 - 7.18 (m, 5H, H^18, 19, 20, 21, 22^), 5.99 (dqd, *J* = 13.8, 6.5, 0.7 Hz, 1H, H^14^), 5.64 (ddt, *J* = 14.9, 10.2, 1.6 Hz, 1H, H^13^), 4.60 (dd, *J* = 10.1, 10.1 Hz, 1H, H^10^), 3.36 (d, *J*= 13.7 Hz, 1H, H^7a^), 2.92 (ddd, *J* = 9.9, 9.9, 2.4, 1H, H^9^), 2.75 - 2.67 (m, 1H, H^8^), 2.70 (d, *J* = 13.8 Hz, 1H, H^7b^), 1.76 (dd, *J* = 6.5, 1.7 Hz, 3H, H^15^), 1.38 (s, 3H, H^6^), 1.26 (d, *J* = 6.9 Hz, 3H, H^16^), 0.87 (s, 3H, H^5^).

**^13^C NMR** (101 MHz, CD_2_Cl_2_, ppm): δ = 202.6 (1C, C^11^), 201.5 (1C, C^2^), 138.0 (1C, C^17^), 134.1 (1C, C^14^), 131.4 (2C, C^19, 21^), 127.9 (2C, C^18, 22^), 126.7 (1C, C^20^), 125.1 (1C, C^13^), 85.4 (1C, C^1^), 80.9 (1C, C^4^), 64.9 (1C, C^9^), 62.6 (1C, C^10^), 45.0 (1C, C^8^), 36.6 (1C, C^7^), 31.7 (1C, C^5^), 30.9 (1C, ^C6^), 18.1 (1C, C^15^), 14.0 (1C, C^16^).

**HRMS** (ESI): found: [*M*+H]^+^ 343.1836, C_20_H_27_N_2_OS^+^ requires 343.1839.

**HPLC** (IC, *n*-hexane/*i*-PrOH 92.5:7.5): *t*_R_ = 9.8 min (enantiomer 1)

11.8 min (enantiomer 2)

-> racemic


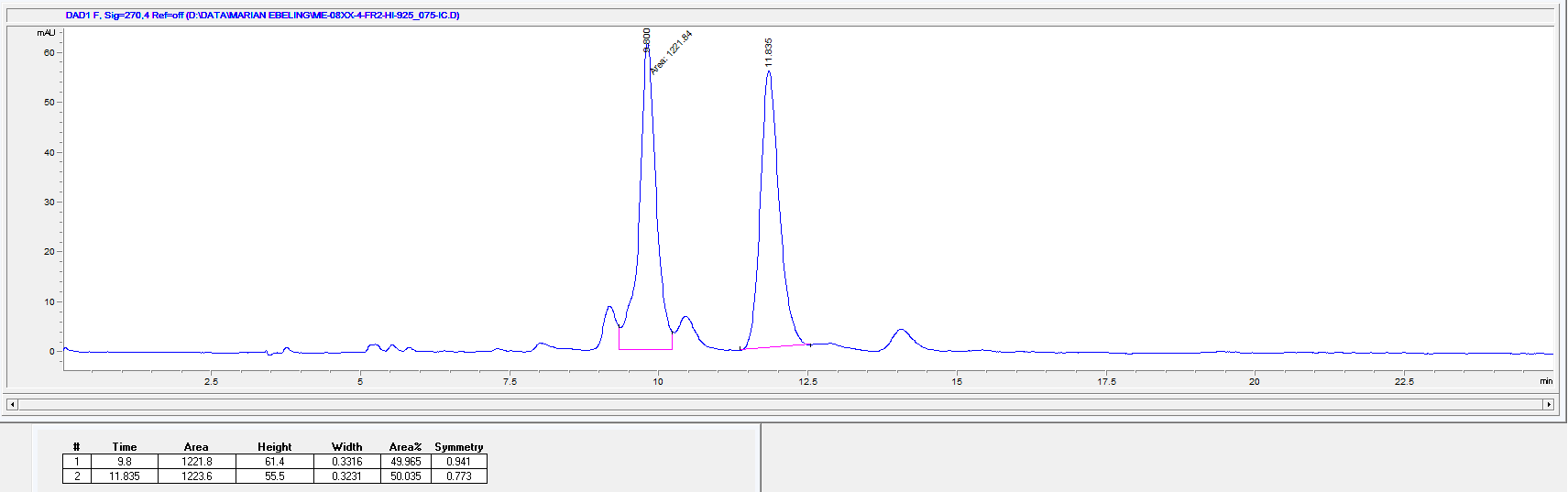


***endo*-14**:

**^1^H NMR** (400 MHz, CD_2_Cl_2_, ppm): δ = 9.60 (d, *J* = 3.6  Hz, 1H, H^12^), 8.35 (d, *J* = 11.4 Hz, 1H, H^3^), 7.39 - 7.17 (m, 5H, H^18, 19, 20, 21, 22^), 5.57 (dd, *J*= 15.3, 6.4 Hz, 1H, H^14^), 5.47 (ddd, *J*= 15.2, 7.2, 1.4 Hz, 1H, H^13^), 4.05 (dd, *J* = 9.5, 7.2 Hz, 1H, H^10^), 3.46 (d, *J* = 13.0 Hz, 1H, H^7a^), 2.69 (ddd, *J* = 11.9, 9.5, 3.6 Hz, 1H, H^9^), 2.56 (d, *J* = 13.0 Hz, 1H, H^7b^), 2.39 (dq, *J* = 11.9, 6.8 Hz, 1H, H^8^), 1.70 (dd, *J* = 6.3, 1.3 Hz, 3H, H^15^), 1.38 (s, 3H, H^6^), 1.35 (d, *J* = 6.8 Hz, 3H, H^16^), 0.40 (s, 3H, H^5^).

**^13^C NMR** (101 MHz, CD_2_Cl_2_, ppm): δ = 202.7 (1C, C^2^), 201.4 (1C, C^11^), 137.9 (1C, C^17^), 133.7 (1C, C^13^), 132.2 (2C, C^18, 22^), 128.2 (2C, C^19, 21^), 127.2 (1C, C^14^), 127.0 (1C, C^20^), 86.9 (1C, C^1^), 81.3 (1C, C^4^), 64.9 (1C, C^9^), 61.1 (1C, C^10^), 46.9 (1C, C^8^), 41.5 (1C, C^7^), 31.6 (1C, C^5^), 26.6 (1C, C^6^), 17.8 (1C, C^15^), 12.5 (1C, C^16^).

**HRMS** (ESI): found: [*M*+H]^+^ 343.1836, C_20_H_27_N_2_OS^+^ requires 343.1839.

**HPLC** (IC, *n*-hexane/*i*-PrOH 92.5:7.5): *t*_R_ = 10.5 min (enantiomer 1)

15.3 min (enantiomer 2)

-> 30 % *ee*


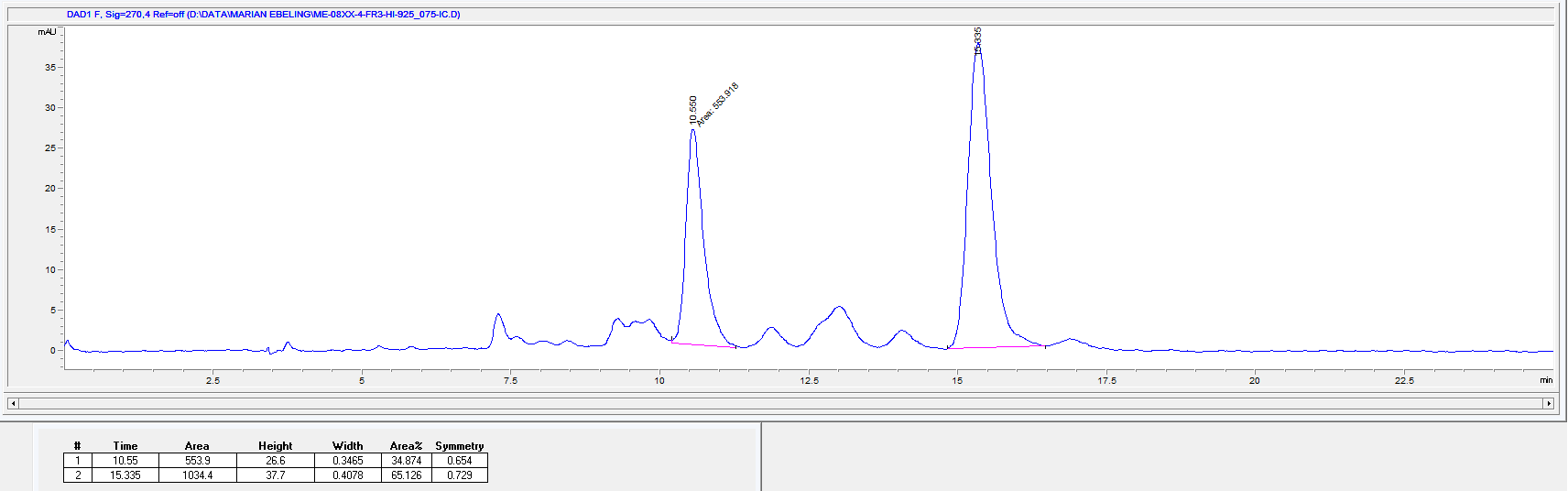


# Computational methods

## General parameters

All calculations were performed using ORCA, Version 6.0.1.^[24]^ Geometries were visualised with Avogadro2 1.100.00.^[42]^ Energy diagrams were created using ChemDraw Energy Diagram Plotter.^[43]^

Conformer search utilised the XTB method^[44,45]^ with the GOAT algorithm and the ALPB solvation model.^[46]^

Structure optimisation, frequency analysis and IRC calculations were performed with the PBEh‑3c composite method.^[25]^ Optimisations used tight convergence criteria. Frequency analysis was used to verify the nature of the geometries (local minima: no imaginary frequencies; transition states: one imaginary frequency)

High-level single point calculations utilised the ωB97X functional^[28]^ with the revised D4 dispersion correction^[29,30]^ and the def2-QZVP basis set.^[31]^

All calculations (except the conformer search) utilised the CPCM solvation model^[26,27]^ (ORCA preset “Acetonitrile”), and the auxiliary def2/J basis.^[47]^ The full workflow is described below:

Local minima (cinnamaldehyde, water, **(*S*)-3b**, **11**, **12**, **13**, **13a, 11-Im4t-adduct, *ent*‑11‑Im4t‑adduct**):

GOAT conformer search from an initial structure resulted in a global energy minimum, which was then optimised at DFT level and the local minimum verified *via* frequency analysis (No imaginary frequencies). This also yielded the thermochemical corrections (*T* = 298.15 K). A high-level single point calculation was performed at this geometry to obtain accurate electronic energies.

Transition states 1 (**11** TS1, ***ent*-11** TS1):

A relaxed scan from the optimised product structure simultaneously stretching the (in the xyz file) C_10_-C_20_ and C_0_-C_21_ bond in 0.1 Å steps was performed. The energetic local maximum (bond lengths: 2.3 ± 0.1 Å) was then optimised to a transition state using DFT and the nature of the geometry verified by frequency analysis (one imaginary frequency). The thermochemical correction (*T* = 298.15 K) was obtained from this, and a high-level single point calculation gave accurate electronic energies at this geometry.

IMs (**11** IM, ***ent*-11** IM)

Starting from the TS1 geometry, an IRC was calculated and followed. The structures obtained from the backwards path were optimised and the energy calculated as described for the local minima.

TS2 (**11** TS2, ***ent*-11** TS2)

The structure of the TS2 were obtained through the NEB-TS (PBEh3c, CPCM(Acetonitrile)) algorithm in ORCA. As input, the corresponding optimised IM and product structure were chosen. The TS2 structures were then optimised and treated as described for TS1.

## ORCA 6.0.1 Keywords

Conformer search:

!GOAT XTB ALPB(Acetonitrile)

Optimisation & Frequency analysis:

!Opt Freq PBEh3c CPCM(Acetonitrile)

%geom Convergence tight END

Single Point:

!wB97X-D4Rev def2-QZVP CPCM(Acetonitrile)

Relaxed Scan:

!Opt PBEh3c CPCM(Acetonitrile)

%geom

Scan B 10 20 = 1.9, 2.5, 7 END

Scan B 0 21 = 1.9, 2.5, 7 END

Simul_Scan True

END

TS Optimisation & Frequency analysis:

!OptTS Freq PBEh3c CPCM(Acetonitrile)

%geom

Convergence tight

Calc_Hess true

END

IRC:

!IRC PBEh3c CPCM(Acetonitrile)

%irc

InitHess read

END

NEB-TS

!NEB-TS PBEh3c CPCM(Acetonitrile)

## Energy calculation

*G*_ges_ = *E*_el_ + *E*_therm.Corr._

With *G*_ges_: solvated Gibbs free energy: *E*_el_ : electronic energy (from single point calc.); *E*_therm.Corr._: thermal correction term (from frequency analysis at 298.15 K; total correction), comprised of zero-point energy, thermal vibrational, rotational and translational correction (combined: inner energy correction), vibrational, rotational and translational entropy (combined: entropic correction).

## Energies of the calculated geometries

**Table S4:** Total energy, electronic energy, thermal corrections and imaginary modes of the geometries calculated in this work.

| Nr. |  | PBEh-3c | | | | ωB97X-D4rev/ def2-QZVP | *G*_ges_  [E_h_] |
| --- | --- | --- | --- | --- | --- | --- | --- |
|  | charge | imag. modes [cm^−1^] | inner energy corr. [E_h_] | entropic corr. [E_h_] | total correction [E_h_] | electronic energy [E_h_] |  |
| cinnamaldehyde | 0 | - | 0.15630681 | −0.04285244 | 0.11439857 | −423.3360326 | −423.2216340 |
| **(*S*)-3b** | 0 | - | 0.28435287 | −0.05365335 | 0.23164373 | −975.1970298 | −974.9653861 |
| water | 0 | - | 0.02475164 | −0.02141935 | 0.00427650 | −76.49290076 | −76.48862426 |
| **11** | 0 | - | 0.57650185 | −0.08599413 | 0.49145192 | −1745.424509 | −1744.933057 |
| **12** | 0 | - | 0.57653517 | −0.08549925 | 0.49198013 | −1745.419364 | −1744.927383 |
| **13a** | 1+ | - | 0.42885001 | −0.06965180 | 0.36014242 | −1322.498599 | −1322.138457 |
| **13** | 1+ | - | 0.42503055 | −0.07184075 | 0.35413401 | −1322.468493 | −1322.114359 |
| **11** TS1 | 2+ | −340.1 | 0.85677301 | −0.11134995 | 0.74636727 | −2644.965543 | −2644.219176 |
| **11** IM | 2+ | - | 0.85896171 | −0.11190522 | 0.74800070 | −2644.985844 | −2644.237843 |
| **11** TS2 | 2+ | −294.2 | 0.85769006 | −0.11049136 | 0.74814291 | −2644.973644 | −2644.225501 |
| **11-Im4t-adduct** | 2+ | - | 0.86000659 | −0.11101153 | 0.74993927 | −2645.011248 | −2644.261308 |
| ***ent*-11** TS1 | 2+ | −322.6 | 0.85667857 | −0.11296522 | 0.74465757 | −2644.957856 | −2644.213198 |
| ***ent*-11** IM | 2+ | - | 0.85949983 | −0.11161442 | 0.74882962 | −2644.982611 | −2644.233781 |
| ***ent*-11** TS2 | 2+ | −348.0 | 0.85799913 | −0.11075266 | 0.74819068 | −2644.965187 | −2644.216996 |
| ***ent*-11-Im4t-adduct** | 2+ | - | 0.86023444 | −0.11140636 | 0.74977229 | −2645.010659 | −2644.260887 |

## Geometries

The final geometries are available in xyz format in a separate file in the Supporting Information.

## Supplementary figures


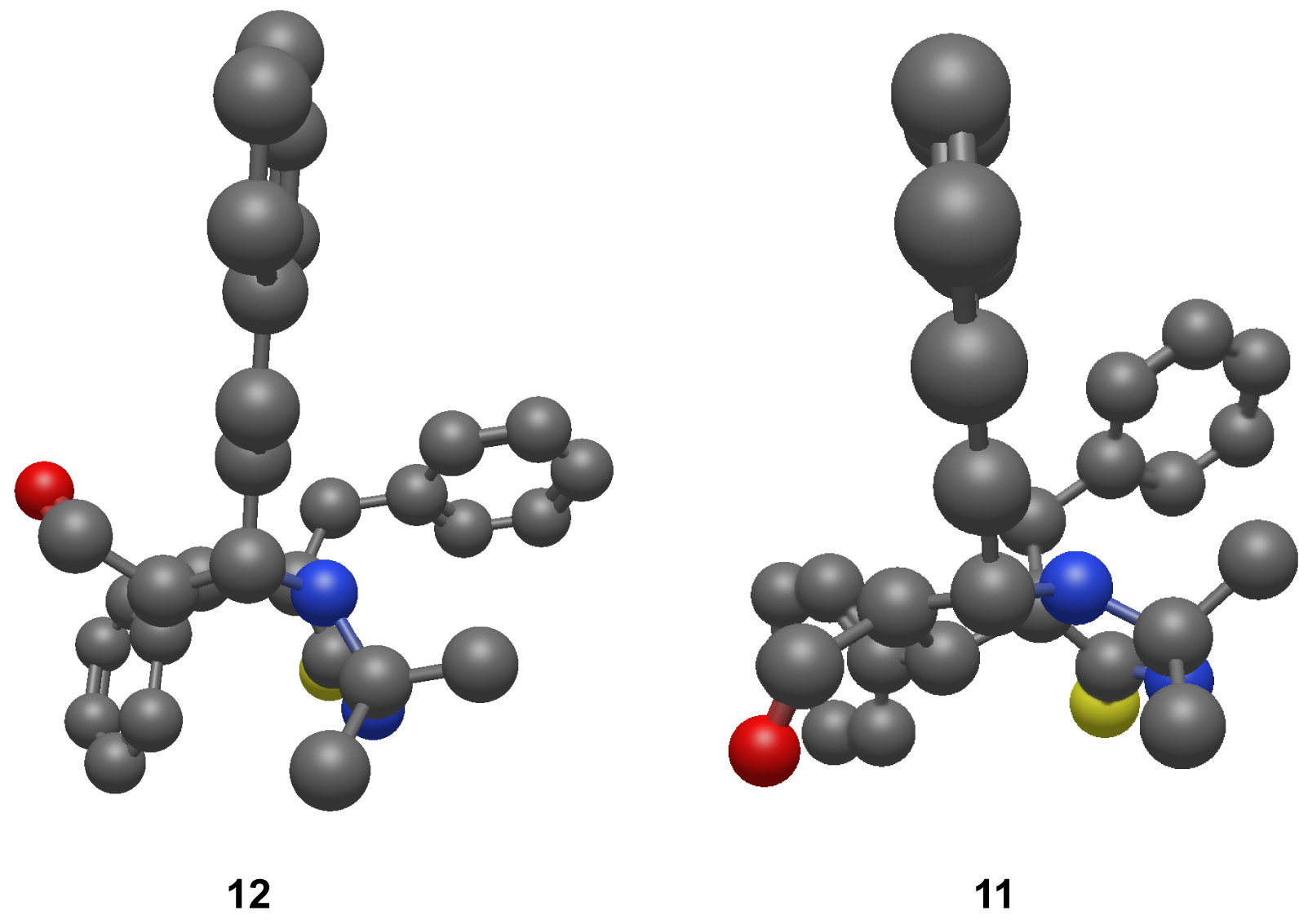


**Figure S6** Visualisation of diastereomeric products **12** and **11**.


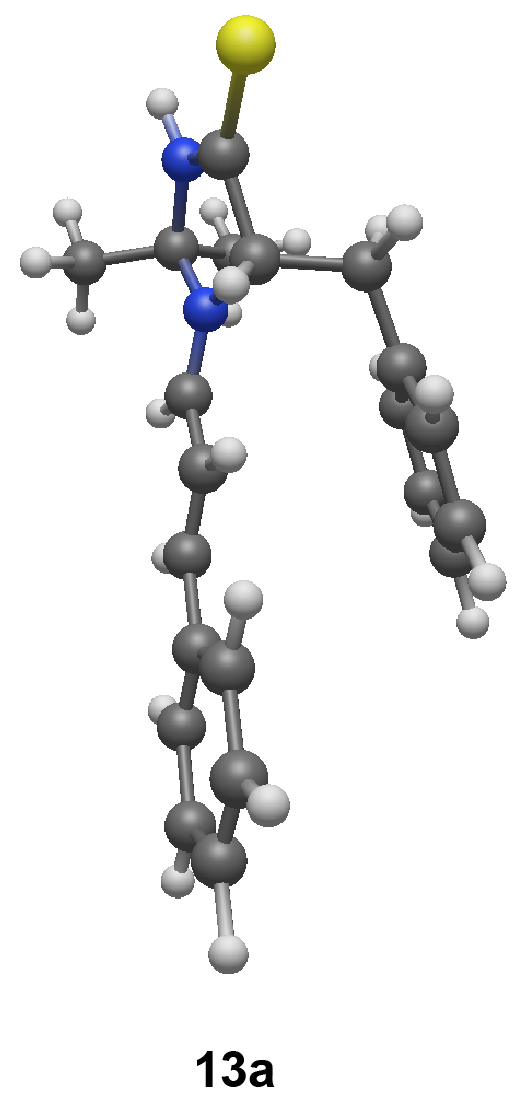


**Figure S7** Visualisation of electrophilic iminium ion **13a** with blocked re-face.


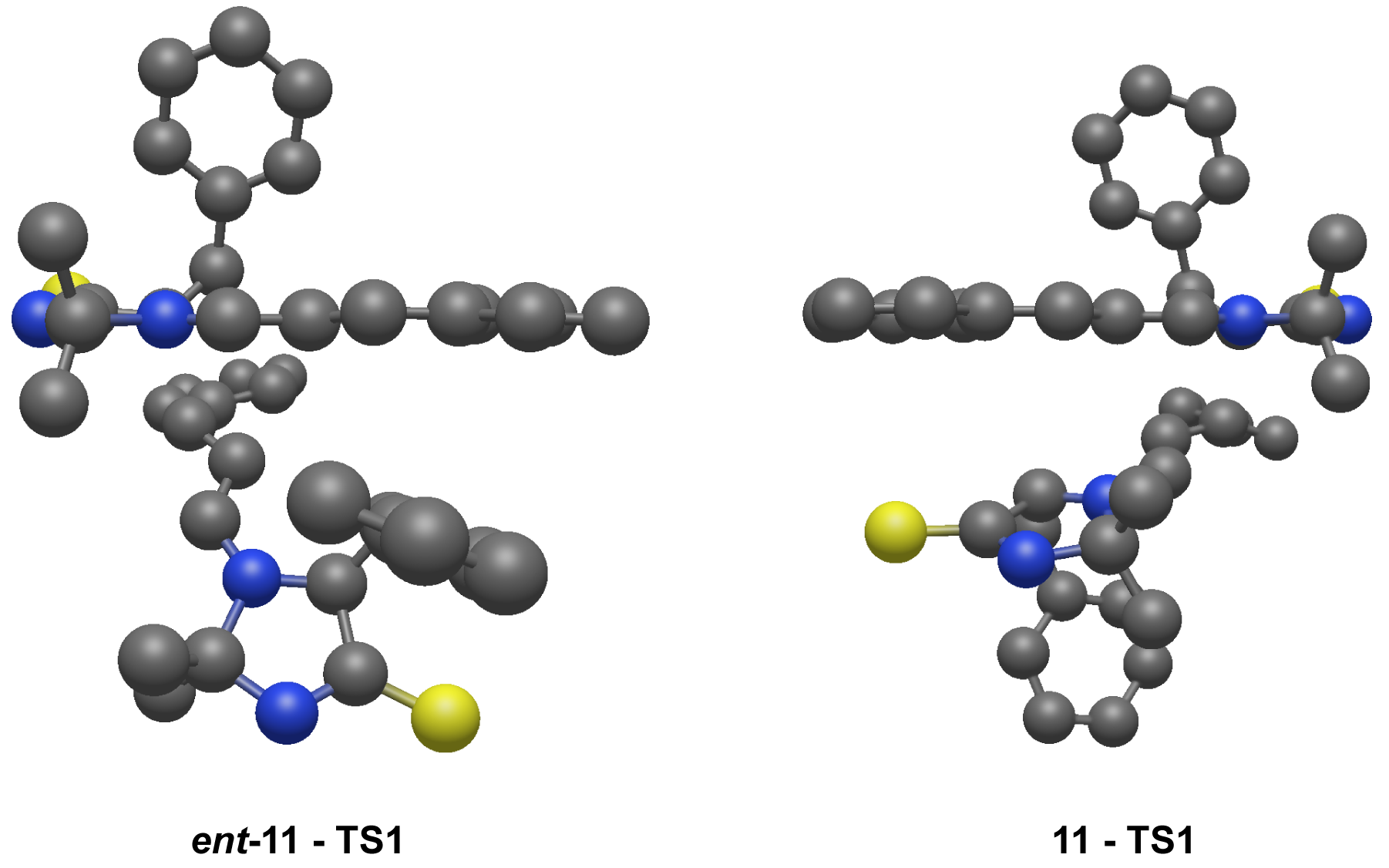


**Figure S8** Visualisation of transition states (TS1) of leading to **ent-11** and **11**.

# Spectra and X-ray structures

## NMR spectra of synthesized compounds

### *tert*-butyl (*S*)-(1-amino-1-oxo-3-phenylpropan-2-yl)carbamate **(*S*)-5a**

### *tert*-butyl (*R*)-(1-amino-1-oxo-3-phenylpropan-2-yl)carbamate **(*R*)-5a**

### *tert*-butyl (*S*)-(1-amino-3-phenyl-1-thioxopropan-2-yl)carbamate **(*S*)-6a**

### *tert*-butyl (*R*)-(1-amino-3-phenyl-1-thioxopropan-2-yl)carbamate **(*R*)-6a**

### (*S*)-1-amino-3-phenyl-1-thioxopropan-2-aminium chloride **(*S*)-7a**

### (*R*)-1-amino-3-phenyl-1-thioxopropan-2-aminium chloride **(*R*)-7a**

### *tert*-butyl (*S*)-(1-(methylamino)-3-phenyl-1-thioxopropan-2-yl)carbamate **(*S*)-5b**

### *tert*-butyl (*S*)-(1-(methylamino)-3-phenyl-1-thioxopropan-2-yl)carbamate **(*S*)-6b**

### (*S*)-1-(methylamino)-3-phenyl-1-thioxopropan-2-aminium chloride **(*S*)-7b**

### (*S*)-5-benzyl-2,2-dimethylimidazolidine-4-thione **(*S*)-3b**

### (*R*)-5-benzyl-2,2-dimethylimidazolidine-4-thione **(*R*)-3b**

### (2*S*,5*S*)-5-benzyl-2-(*tert*-butyl)imidazolidine-4-thione ***trans*-3c**

### (2*R*,5*S*)-5-benzyl-2-(*tert*-butyl)imidazolidine-4-thione ***cis*-3c**

### (*S*)-5-benzyl-2,2,3-trimethylimidazolidine-4-thione **(*S*)-3a**

### (*S*)-5-benzyl-2,2-dimethyl-4-(methylthio)-2,5-dihydro-1*H*-imidazole **8**

### (*S*)-2-((5-benzyl-2,2-dimethyl-2,5-dihydro-1*H*-imidazol-4-yl)thio)acetonitrile **9**

### (*E*)-7a-benzyl-6-(dimethoxymethyl)-3,3-dimethyl-7-phenyl-5-styrylhexahydro-1*H*-pyrrolo[1,2-*c*]imidazole-1-thione **11**

### (*E*)-7a-benzyl-3,3-dimethyl-7-phenyl-5-styryl-1-thioxohexahydro-1*H*-pyrrolo[1,2-*c*]imidazole-6-carbaldehyde **10**

### cyclopenta-1,3-diene

### Compound ***endo*-2** and ***exo*-2**

### Compound ***exo*-14**

### Compound ***endo*-14**

## X-ray crystallography

Crystallographic data have been deposited with the Cambridge Crystallographic Data Centre, CCDC, 12 Union Road, Cambridge CB21EZ, UK. Copies of the data can be obtained free of charge on quoting the depository numbers CCDC-2483175 (**11**) and 2483176 (***cis*-3c**) (<https://www.ccdc.cam.ac.uk/structures/>).^[48]^

### (2*S*,5*S*)-5-benzyl-2-(*tert*-butyl)imidazolidine-4-thione ***cis*-3c**


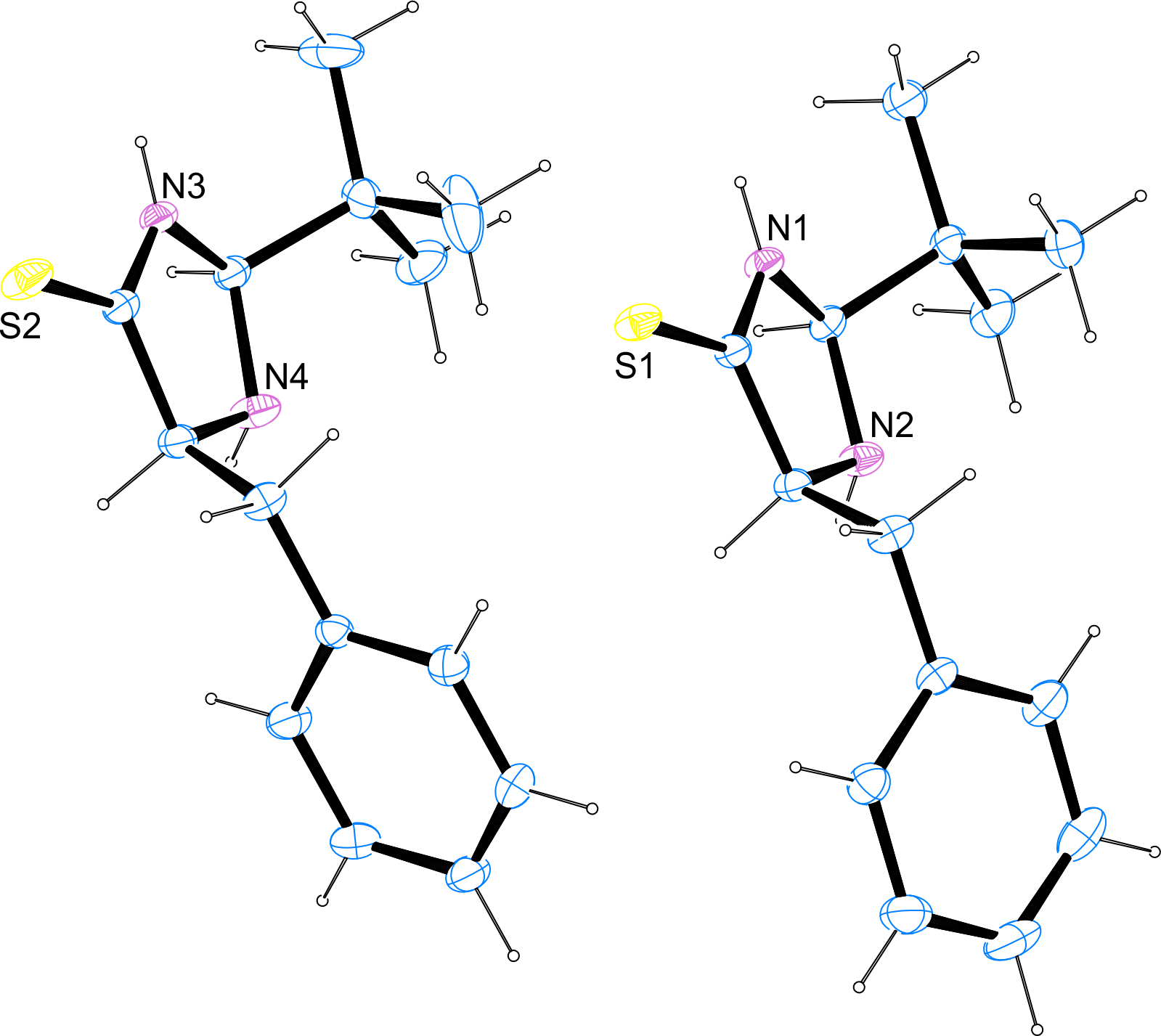


|  | ***cis*-3c** |
| --- | --- |
| net formula | C_14_H_20_N_2_S |
| *M*_r_/g mol^−1^ | 248.38 |
| crystal size/mm | 0.130 × 0.090 × 0.060 |
| *T*/K | 173.(2) |
| radiation | MoKα |
| diffractometer | 'Bruker D8 Venture TXS' |
| crystal system | orthorhombic |
| space group | *P*2_1_2_1_2_1_ |
| *a*/Å | 9.0149(3) |
| *b*/Å | 16.7682(5) |
| *c*/Å | 18.6197(6) |
| α/° | 90 |
| β/° | 90 |
| γ/° | 90 |
| *V*/Å^3^ | 2814.62(16) |
| *Z* | 8 |
| calc. density/g cm^−3^ | 1.172 |
| μ/mm^−1^ | 0.212 |
| absorption correction | Multi-Scan |
| transmission factor range | 0.95–0.99 |
| refls. measured | 49256 |
| *R*_int_ | 0.0335 |
| mean σ(*I*)/*I* | 0.0204 |
| θ range | 2.502–27.103 |
| observed refls. | 5844 |
| *x, y* (weighting scheme) | 0.0385, 0.8079 |
| hydrogen refinement | mixed |
| Flack parameter | 0.003(14) |
| refls in refinement | 6211 |
| parameters | 329 |
| restraints | 0 |
| *R*(*F*_obs_) | 0.0323 |
| *R*_w_(*F*^2^) | 0.0861 |
| *S* | 1.075 |
| shift/error_max_ | 0.001 |
| max electron density/e Å^−3^ | 0.281 |
| min electron density/e Å^−3^ | −0.217 |

### Bicyclization aldehyde compound **11**


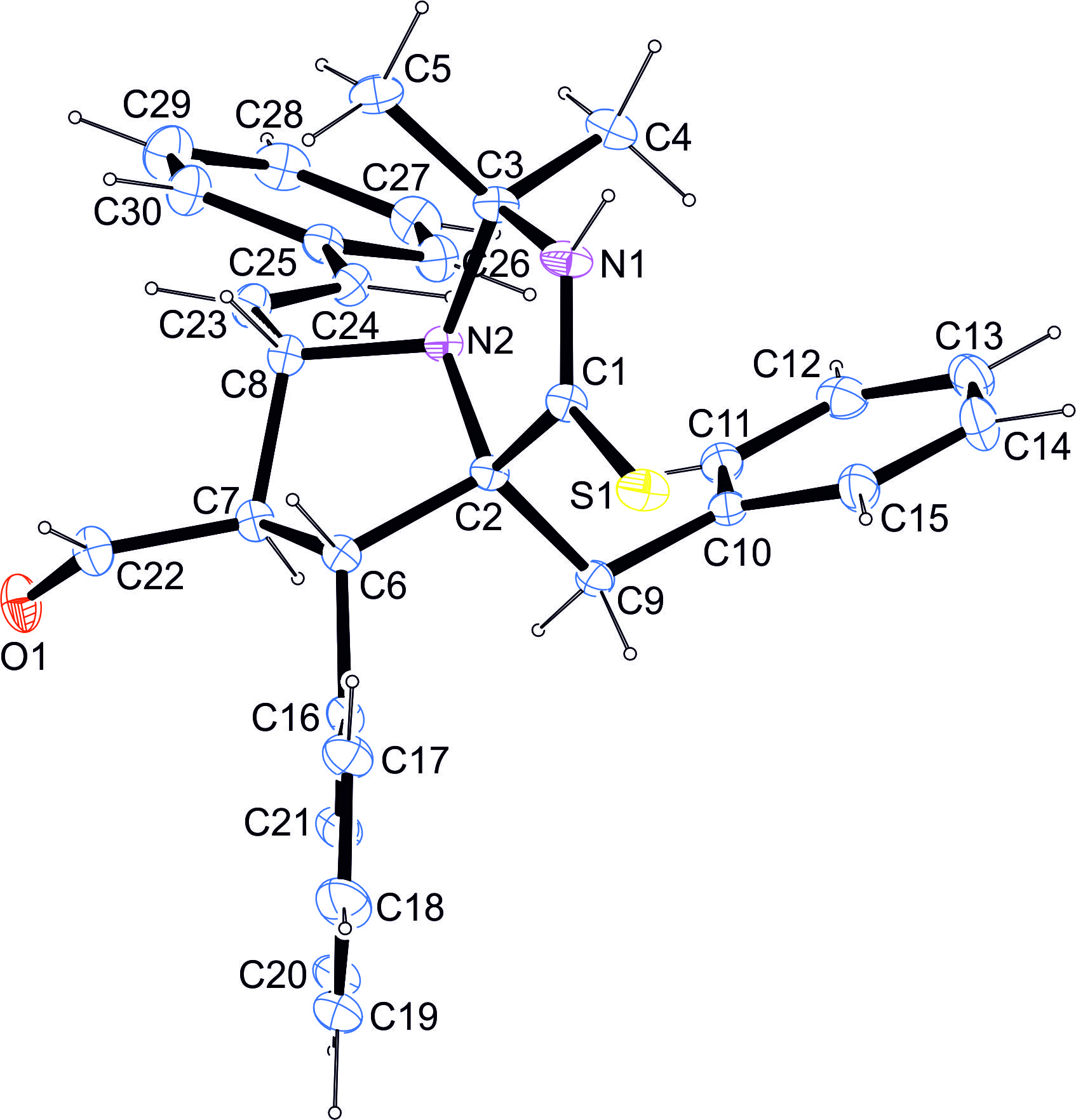


|  | **11** |
| --- | --- |
| net formula | C_30_H_30_N_2_OS |
| *M*_r_/g mol^−1^ | 466.62 |
| crystal size/mm | 0.180 × 0.140 × 0.120 |
| *T*/K | 173.(2) |
| radiation | MoKα |
| diffractometer | 'Bruker D8 Venture TXS' |
| crystal system | orthorhombic |
| space group | *Pbca* |
| *a*/Å | 14.8159(6) |
| *b*/Å | 15.9963(7) |
| *c*/Å | 21.3451(8) |
| α/° | 90 |
| β/° | 90 |
| γ/° | 90 |
| *V*/Å^3^ | 5058.8(4) |
| *Z* | 8 |
| calc. density/g cm^−3^ | 1.225 |
| *μ*/mm^−1^ | 0.153 |
| absorption correction | Multi-Scan |
| transmission factor range | 0.95–0.98 |
| refls. measured | 95731 |
| *R*_int_ | 0.0586 |
| mean σ(*I*)/*I* | 0.0203 |
| θ range | 2.675–27.101 |
| observed refls. | 4692 |
| *x, y* (weighting scheme) | 0.0457, 2.3600 |
| hydrogen refinement | mixed |
| Flack parameter | ? |
| refls in refinement | 5574 |
| parameters | 313 |
| restraints | 0 |
| *R*(*F*_obs_) | 0.0387 |
| *R*_w_(*F*^2^) | 0.1056 |
| *S* | 1.069 |
| shift/error_max_ | 0.001 |
| max electron density/e Å^−3^ | 0.262 |
| min electron density/e Å^−3^ | −0.232 |

# Literature

[4] K. A. Ahrendt, C. J. Borths, D. W. C. MacMillan, *J. Am. Chem. Soc.* **2000**, *122*, 4243-4244.

[22] T. Noguchi, M. Sekine, Y. Yokoo, S. Jung, N. Imai, *Chem. Lett.* **2013**, *42*, 580-582.

[23] R. M. Lemieux, A. J. D. M. Barbosa, J. M. Bentzien, S. R. Brunette, Z. Chen, D. Cogan, D. A. Gao, A. Heim-Riether, J. C. Horan, J. A. Kowalski, M. D. Lawlor, W. Liu, B. McKibben, C. A. Miller, N. Moss, M. A. Tschantz, Z. Xiong, H. Yu, Y. Yu (Boehringer Ingelheim International Gmbh, Boehringer Ingelheim Pharma Gmbh & Co. Kg), WO2009070485A1, **2009**.

[24] F. Neese, *Wiley Interdiscip. Rev. Comput. Mol. Sci.* **2022**, *12*.

[25] S. Grimme, J. G. Brandenburg, C. Bannwarth, A. Hansen, *J. Chem. Phys.* **2015**, *143*.

[26] M. Garcia-Ratés, F. Neese, *J. Comput. Chem.* **2020**, *41*, 922-939.

[27] M. Garcia-Ratés, F. Neese, *J. Comput. Chem.* **2019**, *40*, 1816-1828.

[28] M. Müller, A. Hansen, S. Grimme, *J. Chem. Phys.* **2023**, *158*.

[29] S. Grimme, J. Antony, S. Ehrlich, H. Krieg, *J. Chem. Phys.* **2010**, *132*.

[30] E. Caldeweyher, S. Ehlert, A. Hansen, H. Neugebauer, S. Spicher, C. Bannwarth, S. Grimme, *J. Chem. Phys.* **2019**, *150*.

[31] F. Weigend, R. Ahlrichs, *Phys. Chem. Chem. Phys.* **2005**, *7*, 3297.

[36] G. R. Fulmer, A. J. M. Miller, N. H. Sherden, H. E. Gottlieb, A. Nudelman, B. M. Stoltz, J. E. Bercaw, K. I. Goldberg, *Organometallics* **2010**, *29*, 2176-2179.

[37] Bruker (2012). SAINT. Bruker AXS Inc., Madison, Wisconsin, USA.

[38] G. M. Sheldrick, SADABS, Program for Area Detector Adsorption Correction. University of Gottingen, Göttingen (Germany), **1996**.

[39] G. Sheldrick, *Acta Crystallogr. A* **2015**, *71*, 3-8.

[40] L. Farrugia, *J. Appl. Crystallogr.* **2012**, *45*, 849-854.

[41] K. Ishihara, H. Kurihara, M. Matsumoto, H. Yamamoto, *J. Am. Chem. Soc.* **1998**, *120*, 6920-6930.

[42] M. D. Hanwell, D. E. Curtis, D. C. Lonie, T. Vandermeersch, E. Zurek, G. R. Hutchison, *J. Cheminform.* **2012**, *4*, 17.

[43] L. Yuanhe, “Energy Diagram Plotter CDXML”, can be found under https://github.com/liyuanhe211/Energy_Diagram_Plotter_CDXML, **2023** (accessed: 28.08.2025).

[44] C. Bannwarth, E. Caldeweyher, S. Ehlert, A. Hansen, P. Pracht, J. Seibert, S. Spicher, S. Grimme, *Wiley Interdiscip. Rev. Comput. Mol. Sci.* **2021**, *11*.

[45] C. Bannwarth, S. Ehlert, S. Grimme, *J. Chem. Theory Comput.* **2019**, *15*, 1652-1671.

[46] S. Ehlert, M. Stahn, S. Spicher, S. Grimme, *J. Chem. Theory Comput.* **2021**, *17*, 4250-4261.

[47] F. Weigend, *Phys. Chem. Chem. Phys.* **2006**, *8*, 1057.

[48] Deposition numbers 2483176 (for ***cis*-3b**) and 2483175 (for **11**) contain the supplementary crystallographic data for this paper. These data are provided free of charge by the joint Cambridge Crystallographic Data Centre and Fachinformationszentrum Karlsruhe [Access Structures](http://www.ccdc.cam.ac.uk/structures) service.
