# Supplementary material for: Dual‐Mode Thio‐MacMillan Organocatalysts: Stereoselective Diels–Alder Reactions or Sacrificial Self‐Cyclization to N‐Bridged Bicyclic Lactams
Source: Chemistry. 2025 Dec 18;32(5):e03017. doi: 10.1002/chem.202503017 (PMC12865155; doi:10.1002/chem.202503017)
Supplement: Supplementary file 3 — Supporting File 3: chem70572‐sup‐0003‐SuppMat.docx [file CHEM-32-e03017-s002.docx]

**Geometries (in xyz format)**

Cinnamaldehyde

18

*E*_El_(SP) = −423.3360326 E_h_

C -14.34071604270703 -0.41463365216322 0.19754136248255

C -14.27060888421907 -1.78852226482749 -0.01195175593677

C -13.04064565286331 -2.39048527162444 -0.23590702296435

C -11.88716173321386 -1.62156330863512 -0.24901993369708

C -11.94277013248715 -0.24106832544585 -0.03921198713454

C -13.19002155308471 0.35288197463745 0.18438632660290

C -10.69961633824284 0.51686399012156 -0.06141834725628

C -10.54406081678747 1.83772678840715 0.11963769505104

C -9.20584330777299 2.40857151337168 0.06158307976796

O -8.95191533872131 3.58431849368580 0.21060831718504

H -15.29843396925022 0.05786421111489 0.37163479584125

H -15.17356609671494 -2.38488478037026 -0.00076531346912

H -12.97870750211827 -3.45803219316087 -0.40061791576526

H -10.92850089931832 -2.09488410839863 -0.42457765544364

H -13.26729611758215 1.41997440433573 0.34894135347900

H -9.80482132362030 -0.07330831586958 -0.24601188837148

H -11.37467095020444 2.50795543860204 0.30964815938952

H -8.40135834245083 1.67505340785471 -0.13449927505251

## **(*S*)-3b**

31

*E*_El_(SP) = −975.1970298 E_h_

C -10.17506757841631 1.19444129517344 1.10537495673699

N -11.20365634646498 1.73735658298510 0.23034640481715

C -10.87069059119032 1.48010911442118 -1.16886012593136

N -9.60400101670006 0.75656104560446 -1.05305489412072

C -9.14560398195994 0.61039797058870 0.16549689410174

S -7.69389188202289 -0.05615764077954 0.61734712990305

C -10.64244576402361 2.78085041658186 -1.93184486821016

C -11.91641050577143 0.61817757832900 -1.86485058923513

C -10.68868312246366 0.17011545892541 2.13259339387045

C -11.50093181144852 -0.93801109505385 1.52403781431354

C -12.89280298770957 -0.86590339064392 1.51939517054784

C -13.65981967392525 -1.84940610036713 0.90868105973734

C -13.04203120303196 -2.92890837940501 0.29364476098373

C -11.65577871189595 -3.02129025325976 0.30489136776151

C -10.89321905398194 -2.03597401246607 0.91590456835700

H -9.67549078838764 1.98774787995628 1.67356925469855

H -12.11549722920642 1.35938780531383 0.44823539958411

H -9.07240070469364 0.48030370501447 -1.86737988722658

H -11.57480524525438 3.34291078102848 -1.98424516573688

H -9.89799314124916 3.39696655865270 -1.42744602902642

H -10.30485550914254 2.59033441254107 -2.95185494428187

H -12.87218864181559 1.14307823531808 -1.88951748023424

H -11.62588959882967 0.40961653177009 -2.89465357788505

H -12.05353564505795 -0.32890703700190 -1.34182800356533

H -11.29364041499401 0.70871144522447 2.86498656918583

H -9.82079155338172 -0.22187379821193 2.66596084664021

H -13.38453498847012 -0.03080493673430 2.00649583364152

H -14.73949130305085 -1.77173786552062 0.91612244190696

H -13.63553135047358 -3.69795631033300 -0.18369943991313

H -11.16558584427135 -3.86628965999928 -0.16169280602279

H -9.81340381840068 -2.12183232514928 0.92260393295024

## Water

3

*E*_El_(SP) = −76.49290076 E_h_

O -0.00000000000228 -0.10188921756703 0.00000000000000

H -0.75627046011301 0.48919460878289 0.00000000000000

H 0.75627046011528 0.48919460878414 0.00000000000000

## **11**

64

*E*_El_(SP) = −1745.424509 E_h_

N -7.85376887248354 2.02801098629827 -0.00552840563888

C -8.97927358562296 2.68296983720407 -0.15940138069848

C -10.08931546489753 1.65973174557253 -0.23270259838512

N -9.38773202388148 0.38375695914798 -0.14084191259683

C -7.94714971397887 0.57020170469453 0.02341606356227

C -9.89789390977970 -0.59234792861617 -1.09195393142186

C -11.10501549646355 1.87622205215439 0.90827655478358

C -10.24598630384173 -1.88794544634383 -0.43262898322491

C -9.78492714253452 -3.07012940910107 -0.84172707233067

C -11.12388057903735 0.11800375454449 -1.71066370051060

C -10.77364396715748 1.59818584988353 -1.63054008941964

C -11.39627100776972 -0.40363268830527 -3.08781447333522

C -7.45734513812531 0.04004217437827 1.36779357447384

C -7.08019431647249 0.00469071253216 -1.09968587495527

S -9.15095787664247 4.32991764932124 -0.24777235390310

O -11.36936832898559 0.26005780202432 -4.09234021593402

C -10.09043374895018 -4.38158684023371 -0.26297453615397

C -9.48918278414581 -5.51556301356582 -0.81320471851655

C -9.74475135633196 -6.78144699292219 -0.30526519075339

C -10.61041022212764 -6.93803704487395 0.76734941360710

C -11.21637909723141 -5.81762313226785 1.32623465047100

C -10.96110462252682 -4.55534183789577 0.81791214470586

C -10.54265249101854 1.64852374250665 2.28170613214461

C -10.67065474837321 0.40279798069470 2.89296657327107

C -10.14082846632329 0.16407798719041 4.15264937776277

C -9.46565899042773 1.17382231961523 4.82573215050398

C -9.34013630311522 2.42339138695313 4.23343379005612

C -9.87913022131510 2.65858084272079 2.97539263870564

C -11.90513069233663 2.54873707882609 -1.91785816987006

C -11.65023498630939 3.70614009116342 -2.65199776169125

C -12.66000263675611 4.61030477514060 -2.94456899354621

C -13.95632369146815 4.37158698870553 -2.50822834978144

C -14.22892445642445 3.21720478947577 -1.78853714709525

C -13.21429280314623 2.31343554672822 -1.50167057387084

H -6.96465446668204 2.50024975802122 0.08875101222530

H -9.17484232313814 -0.78662079517256 -1.89135085359967

H -11.50503710627892 2.88773230666487 0.82024599288326

H -11.94011238780207 1.18904270524159 0.75964202879331

H -10.91935366778468 -1.80600846793481 0.41600746396752

H -9.10769278574298 -3.08772365480371 -1.69237800165333

H -12.00491158452283 -0.12591918399964 -1.10426956195247

H -9.98599991475856 1.79066896907632 -2.36742434179803

H -11.62471202743515 -1.48403187182662 -3.12660070807194

H -8.01471175241256 0.48615314657473 2.18911685804564

H -6.39536066495466 0.24933525123375 1.50583971199312

H -7.59541428681369 -1.04081946562878 1.40498135161511

H -7.10587136950942 -1.08490132599569 -1.11242356409180

H -7.38451956948008 0.37846516553306 -2.07744038352207

H -6.04432553615242 0.30139549182262 -0.93305071710084

H -8.81104560872312 -5.40242665038577 -1.65103651800015

H -9.26661402892318 -7.64510335286272 -0.74906208150046

H -10.81339467190808 -7.92317408916201 1.16662849352933

H -11.89350482858500 -5.92984702408109 2.16327083177566

H -11.44769835655487 -3.70039040679067 1.27015440652153

H -11.19079265150798 -0.39176296030396 2.37173762586240

H -10.25234442045536 -0.81224206231248 4.60693786643574

H -9.04823754101243 0.99083233664302 5.80757321264336

H -8.82598985518184 3.22118880449119 4.75438479516339

H -9.78190512373605 3.63977496651402 2.52710306213955

H -10.64314121704574 3.90132521422065 -2.99770069913187

H -12.43398796681415 5.50023141310734 -3.51791715379789

H -14.74797451643884 5.07387463289143 -2.73536156515783

H -15.23708888316900 3.01173689309390 -1.45234542978962

H -13.46874685562373 1.41606881774495 -0.95218677615427

## **12**

64

*E*_El_(SP) = −1745.419364 E_h_

N -9.35066211323156 1.15233420163452 1.78080600913407

C -10.09622901815011 2.02725504934733 1.14776201256835

C -11.03585697830001 1.24936003653015 0.24912205984897

N -10.71676171615848 -0.14186921648439 0.54631320488044

C -9.60542444493647 -0.25356085810770 1.49238611085520

C -10.72936847520188 -0.93202983052740 -0.66935856310229

C -12.49990998996346 1.61800772604572 0.59681682162578

C -12.08620405121422 -1.52264504017282 -0.92567229919911

C -12.30035453087583 -2.83547306877729 -1.01473702904196

C -10.30161898985405 0.07116686160366 -1.76875112957127

C -10.81904594758055 1.43208616077351 -1.30381032159090

C -10.73946965236925 -0.35094582283849 -3.13883618467437

C -10.03979968769210 -0.97787490398643 2.76325296125674

C -8.32471874151301 -0.88859696978245 0.95121697869944

S -10.04806628580426 3.67104088006255 1.34992530498308

O -11.43671891281090 0.29652996544196 -3.87801932303342

C -13.58734975468777 -3.49888772341173 -1.24344534911599

C -14.80940767128155 -2.81911197437256 -1.22403716373305

C -15.99812804323724 -3.49331430277965 -1.44714849162460

C -15.99633640333674 -4.86260091534654 -1.69110225562182

C -14.79216735466040 -5.55139292904898 -1.70620090051866

C -13.60175333290942 -4.87476563198471 -1.48172670259628

C -12.93624731898826 1.19202653171629 1.96850372987211

C -12.77265632109004 2.01664486871329 3.07956054482552

C -13.15614195372202 1.59306453192345 4.34564741375660

C -13.72078914906848 0.33739683083976 4.52126249581686

C -13.90726283350853 -0.48689326981010 3.41897592749912

C -13.51596486522378 -0.06145295787675 2.15789358344724

C -9.96196221154703 2.57752565385647 -1.77156279166467

C -10.55003399928429 3.65418024758385 -2.42751213279129

C -9.78602343794804 4.72044980001613 -2.88274828894594

C -8.41136142501042 4.72061845807797 -2.69506740679155

C -7.81048421418029 3.64612711434845 -2.05093141221137

C -8.57936735693947 2.58653431060604 -1.59602687700187

H -8.67098029100562 1.43112890415758 2.47517084294451

H -10.01592853180001 -1.75358563535392 -0.60231775336073

H -12.60616801307618 2.69737399023051 0.46780785286993

H -13.15100718486281 1.15196204733447 -0.14501565566789

H -12.90868776435436 -0.82456383204192 -1.05015845855606

H -11.44870876875253 -3.50376393512511 -0.91237201903196

H -9.20583901354155 0.06074170751821 -1.79790555707632

H -11.81146332466983 1.59006118821757 -1.73405172216622

H -10.35419378269150 -1.34021434859121 -3.44512180551697

H -10.91511577514794 -0.50123745001958 3.20177101169047

H -9.23698022156491 -0.99519012133743 3.50218159807754

H -10.29667395841477 -2.00980266742498 2.52207457851790

H -7.52812093903362 -0.76314650351908 1.68514185027975

H -8.44497577342113 -1.95929309031397 0.79099719308407

H -7.99859761866178 -0.42470535775948 0.02106713819616

H -14.84064407120362 -1.75504529811220 -1.02509552193669

H -16.93340695788134 -2.94890448241534 -1.42577754616490

H -16.92749536058668 -5.38644712335749 -1.86329893995224

H -14.77741632607365 -6.61765636144175 -1.89141968754982

H -12.66574617152700 -5.42098087424955 -1.49402285656458

H -12.33941781155495 3.00102028048386 2.95440952465347

H -13.01923231104964 2.24995555563618 5.19531274185843

H -14.02287875932975 0.00762326851274 5.50708304528151

H -14.35694806606171 -1.46415968249314 3.54107236814048

H -13.66811184777155 -0.71326269093841 1.30605113786729

H -11.62289577559456 3.66156646768450 -2.58071581610958

H -10.26599194831092 5.54848845481273 -3.38856329452678

H -7.81135454727941 5.54806123634629 -3.05113220797347

H -6.73864127068230 3.63422835619153 -1.89977181211929

H -8.08884465614021 1.76583520512060 -1.08576875074974

## **13a**

47

*E*_El_(SP) = −1322.498599 E_h_

S 2.67057477943137 -0.11274788163325 -4.73803296062516

C 1.21584745083853 -0.18772224226766 -3.97497998891602

N 0.01527791244554 -0.24627243107721 -4.51666993839757

C -1.11805369009057 -0.27745157744337 -3.60932600110293

C -2.06927601863180 0.86636473921343 -3.92667391381586

C -1.82461949232144 -1.62431311696773 -3.63078143731861

N -0.39991009307398 -0.05901810704352 -2.32728417889833

C 1.03964752024152 -0.26233793325718 -2.48064847483837

H 1.59073532666174 0.53952251754360 -1.98834403359907

C 1.52164639258117 -1.64342183731965 -1.97630174090927

C 1.18456777308801 -1.90832940697441 -0.54006798790565

C 2.01793207126690 -1.45071442595874 0.47918310918105

C 1.66763815968381 -1.62608277345146 1.80889834030777

C 0.47874073917968 -2.26600738917831 2.13699252538774

C -0.34515555899709 -2.74735748583841 1.12972383670474

C 0.00867263628213 -2.57178626986579 -0.20089806116780

C -1.00716187650353 0.32628981722134 -1.24847639539612

C -0.39752151811102 0.73862963478790 -0.04244253360240

C -1.19417556548364 1.00706065720776 1.02015885861904

C -0.76620040441382 1.44899735529969 2.32657157767032

C -1.73934468231477 1.62940225596821 3.31603991840692

C -1.38492851935503 2.04690977647098 4.58780593077830

C -0.05171808903081 2.29283923436737 4.88593942654115

C 0.92650636743123 2.12098738875691 3.90988826530545

C 0.57597919577607 1.70220279307589 2.64147049119035

H -0.12792060112645 -0.25878563349746 -5.51804911894051

H -2.94415701807194 0.85457098960875 -3.27812013518981

H -1.56862260307976 1.82968078004635 -3.84046509971939

H -2.42653431921245 0.74912521544232 -4.94942513718708

H -2.62331015567244 -1.64554708651148 -2.88944775019866

H -2.27239615689687 -1.78028529164118 -4.61166770283605

H -1.13544175610617 -2.44304406520282 -3.43151799204756

H 1.10004816362143 -2.42163343395228 -2.61490328192392

H 2.60137097212501 -1.65293390377859 -2.13028381097555

H 2.94041056821347 -0.94068354631694 0.22604320738163

H 2.31945276724198 -1.25803464630584 2.59057102649653

H 0.20140446651661 -2.39565625751524 3.17502675233473

H -1.26568704981861 -3.25930633575033 1.37848041519560

H -0.63868770878779 -2.94961884127453 -0.98348198883620

H -2.09015027764114 0.35634381371306 -1.30166676472359

H 0.67645725857731 0.83310516800613 0.01915592908186

H -2.26562558530887 0.87382737408360 0.89850998354568

H -2.77934208706473 1.43748567618817 3.08205091153219

H -2.14595144258666 2.18071897878465 5.34460412409715

H 0.22927200096804 2.61955137140197 5.87854723852134

H 1.96455601308144 2.31487673463552 4.14422567525963

H 1.35045374593023 1.57399964589383 1.89716889255771

## **13**

47

*E*_El_(SP) = −1322.468493 E_h_

S 4.54040139701626 1.29405991201514 -0.37442129787531

C 2.91076415224941 1.67559887632034 0.08696167593312

C 1.82120960173590 0.93228208072948 -0.23292101773856

C 1.80371714228113 -0.44092737493370 -0.80157186786823

C 1.28806444808113 -1.50488476236212 0.14527598854934

C 0.68512428137485 -2.65002899448959 -0.36934892140660

C 0.22540218547779 -3.65129828450413 0.47358812485947

C 0.36325677676865 -3.52015160065914 1.84922879663491

C 0.96729905315157 -2.38464544411078 2.37042265532333

C 1.42832194592906 -1.38463519612946 1.52398067829006

N 0.70168051321176 1.63554120113378 0.23772084400574

C 1.16091543872814 2.87286008674384 0.96211173185957

C 0.62850388288819 4.11528200053628 0.26885552146541

C 0.76948463173170 2.79652014747426 2.42881930087536

N 2.60448222581769 2.77416153255770 0.81870949286330

C -0.57182683414808 1.37652333243603 0.16769075030814

C -1.23333661376493 0.31444379402661 -0.50445326955769

C -2.57553329146139 0.20690308154498 -0.37891290345860

C -3.42597257335820 -0.79804750009604 -0.98408016673652

C -4.80339741897939 -0.72800973847981 -0.74626217762582

C -5.66771051155023 -1.66210641318093 -1.29298410657489

C -5.16794000655493 -2.68502537280830 -2.08708320234482

C -3.80044791762644 -2.76790580910745 -2.33216973337462

C -2.93631054857099 -1.83618662383441 -1.78823102805267

H 5.13819401817517 1.93174102834012 0.64046394536657

H 1.25684582642120 -0.47743401866682 -1.74699704551773

H 2.83391206461158 -0.68847174533075 -1.07221615140168

H 0.56774837556530 -2.75645850225170 -1.44222448998081

H -0.24456141021808 -4.53185487524796 0.05488949456059

H 0.00136609139373 -4.29704297017450 2.50996630834856

H 1.08100262412764 -2.27372358838971 3.44111621236879

H 1.89903454265819 -0.50603028058872 1.94969552087103

H -0.45504420848548 4.18385799626691 0.35966346465266

H 1.05397007334339 4.99934107136840 0.74330755676123

H 0.89709905189772 4.12007182189541 -0.78694940696800

H 1.21407931830141 3.64028722151112 2.95524140464919

H -0.31060413206481 2.85306100851688 2.56002498306302

H 1.13518212028255 1.87398676323226 2.87827819656926

H 3.12075712803335 3.63132202963619 0.67402691495699

H -1.20751255749183 2.08383604154784 0.68644326767812

H -0.68374043063979 -0.37630024320700 -1.12123749775790

H -3.08934161089625 0.93638378199735 0.24080696154084

H -5.19558100916628 0.06873482181429 -0.12589265052803

H -6.72973523531226 -1.59248527994072 -1.09909841246155

H -5.83946386338878 -3.41749206291380 -2.51535305608573

H -3.40906676818398 -3.56424930395569 -2.95112927020154

H -1.87759196248338 -1.92330365110323 -1.99435209634868

**11** TS1

94

*E*_El_(SP) = −2644.965543 E_h_

C -4.29677950916027 0.47590437935821 2.64342489931224

N -3.20776494147360 -0.34939780893076 2.26472302631303

C -1.96085817223569 0.01112208174852 3.01378130386981

N -2.48299633207510 1.07562957584194 3.85241464178795

C -3.77432972591586 1.24244438865470 3.72781893460955

S -4.76548422184314 2.23353405274849 4.70679594131885

C -5.70172355109863 -0.06596092469373 2.71094597388229

C -1.49706783332395 -1.15052777516787 3.88118909021111

C -5.83817467762643 -1.39913015839433 3.41322265738513

C -0.85687433853515 0.52251480381742 2.10558101350066

C -3.16528611549681 -1.32356896429232 1.39564482125761

C -4.20404546775119 -1.87907911084762 0.61313935891571

C -3.90892651252435 -2.92753678891149 -0.19244369138725

C -4.81512490875025 -3.60213086347931 -1.09347001831114

C -6.17051144970295 -3.26330879353094 -1.21164072572730

C -6.98311402242148 -3.93571489759210 -2.10448578190157

C -6.46359105854094 -4.95855053618780 -2.89229912545939

C -5.12353830584144 -5.30579955993769 -2.78504125183005

C -4.30645457532316 -4.63242690793585 -1.89350179765548

C -3.39386138109939 0.84985212488872 -0.88767444998902

C -4.49972916438862 1.15775394890865 -0.12811539341220

C -4.36372478229244 1.93742877180456 1.03915646716910

C -5.42463727233681 2.86161512915821 1.45708817912161

C -6.77944547280012 2.63470648396772 1.19398471494015

C -6.00730946716290 4.99484678291388 2.44117839823138

C -5.05454347841929 4.05326752295501 2.08515234760855

N -3.38252464529298 0.23196821488393 -2.05755314289004

C -7.73161000343844 3.56500192893069 1.57166422101464

C -7.34938106960907 4.74855571177870 2.19392443163885

C -4.55909012181006 -0.21410952990153 -2.79083547344170

C -3.95072371733437 -0.98350144597703 -3.93600627756101

N -2.64685823252361 -0.82395585116064 -3.88251079426506

C -2.12105881580948 -0.07104959642682 -2.75865083389238

C -5.49758889994837 0.90892290706460 -3.27876301076394

C -6.63053666910735 -2.39502131726027 2.84869222026834

C -6.79788737251211 -3.61864958512796 3.48208640876176

C -6.17157071056231 -3.86430457193219 4.69532774251962

C -5.38761956467486 -2.87443044186268 5.27261995787410

C -5.22618708192265 -1.65118609766938 4.63801212305916

C -1.20806762386861 -0.95848016694385 -1.92196495671403

C -1.39376454875024 1.18198479767785 -3.22862077980421

S -4.80655198371105 -1.85268386456705 -5.04748259206613

C -4.78930251474369 2.04450848082516 -3.95655546661147

C -4.44714453128373 3.18759260091237 -3.23785428821644

C -3.75068730901307 4.22529145443757 -3.84206157658106

C -3.38712928421999 4.13362938201442 -5.17874795033563

C -3.73605996813967 3.00477882045038 -5.90872227483336

C -4.43439743110786 1.96977381093734 -5.30236252277971

H -1.89075896907357 1.55107031000429 4.52058350632658

H -3.80076362369859 2.70868496075257 5.50686627488163

H -6.31359229834252 0.67779639519022 3.22878032521530

H -6.14756624085284 -0.13066293661124 1.71880766869255

H -2.30827250451069 -1.52672325501442 4.50180095810073

H -0.68869641149179 -0.80951897574875 4.52695903301190

H -1.10898968487442 -1.96349754424918 3.26894376394398

H -0.00947641173544 0.82546281368326 2.71974454868046

H -0.51199372658623 -0.26139136801818 1.43345277913349

H -1.17796924211568 1.38294611632418 1.52215451301109

H -2.19285598769143 -1.78988859520095 1.28886079838554

H -5.19240157475993 -1.45353149920412 0.62434211187734

H -2.89240419746695 -3.31028854545368 -0.18049904323608

H -6.59790116669359 -2.47599146878425 -0.60380485354723

H -8.02714135071408 -3.66527935504954 -2.18803435056237

H -7.10481947847177 -5.48159218183242 -3.58943994203972

H -4.71623051559894 -6.09863404230204 -3.39781883568809

H -3.26013688641833 -4.90100824153419 -1.81265649271880

H -2.42064616905343 1.16802965373912 -0.53038989884851

H -5.48802550649292 0.86892326803407 -0.45923965056288

H -6.21140565253611 0.42978393842685 -3.95102265490826

H -7.10529736067311 1.73446621875650 0.68959681194423

H -5.70011282527792 5.91631071181257 2.91743310023022

H -4.00783888881801 4.24930680310473 2.28302754084365

H -8.77669019008913 3.37234033720267 1.36893954755279

H -8.09727375474816 5.47718703204876 2.47782691739159

H -5.15332909822851 -0.90454041865456 -2.18501756882266

H -2.02344067459346 -1.25198265196634 -4.55395094040789

H -3.36187906204686 2.31102017738427 1.23396986860379

H -6.07501593276260 1.28028016929305 -2.43330610174495

H -7.12692915797136 -2.21327784347341 1.90247998001075

H -7.41511706047684 -4.37966546697124 3.02277784820944

H -6.29498962931217 -4.81895850995748 5.18958489470093

H -4.90212679958004 -3.05146114371713 6.22347733888531

H -4.62290146249776 -0.89267936710376 5.12205622383883

H -1.72499154790169 -1.86716582325832 -1.62001425587335

H -0.86006166186991 -0.43486931915078 -1.03197970712658

H -0.32909201722494 -1.23451783394563 -2.50413410674429

H -0.52958704151754 0.89666162809235 -3.82810660330815

H -2.04412555746729 1.81578461892411 -3.82652048045158

H -1.02845810119115 1.75441996819439 -2.37603629265866

H -4.72981148216079 3.27034221143346 -2.19449510060663

H -3.49260377255032 5.10599048550266 -3.26808962111594

H -2.84300599602517 4.94079618929188 -5.65186687353892

H -3.46790655068767 2.93121178038710 -6.95483783946101

H -4.70905290752845 1.09618994375109 -5.88140981647399

**11** IM

94

*E*_El_(SP) = −2644.985844 E_h_

C -4.42756286273035 0.44593594268500 2.43475410318094

N -3.37075450938310 -0.56920216008966 2.27332733286195

C -2.24402688556753 -0.39857028304373 3.22595728127563

N -2.75888613392110 0.71732149813287 4.00908887828423

C -3.90419480308315 1.17306657800434 3.64923459612686

S -4.77002289379083 2.45013040864541 4.36353845975444

C -5.81256367191961 -0.17402376047884 2.73493620391823

C -2.07107202261422 -1.61456001405987 4.12253760135233

C -5.76874045531447 -1.25675951871679 3.78432663516785

C -0.95210948063894 0.00311111248360 2.53121542120801

C -3.30830720576896 -1.54531534522280 1.40908024865483

C -4.26268257174429 -2.02278663184224 0.49120837621346

C -3.87659196465158 -3.01525065305401 -0.35291636677890

C -4.67981183554174 -3.62426298666847 -1.38218275524666

C -6.04413545690889 -3.34444330140605 -1.55108268294579

C -6.75738237345383 -3.95063418885942 -2.56685867514203

C -6.12613753891120 -4.84130328077543 -3.43042965896091

C -4.77636697756176 -5.12833786427041 -3.27425174626710

C -4.05939346558086 -4.52722638893558 -2.25526811836852

C -3.47927378338171 0.67105752259647 -0.96027522071754

C -4.52473937007705 0.80338336051094 -0.12217583866771

C -4.35916774521132 1.46191404907592 1.20264275752466

C -5.29624147212895 2.64691180928312 1.31845069907676

C -6.67895593536364 2.52745638464792 1.18801566305109

C -5.56102331719403 5.04379659721691 1.51337590082191

C -4.75010260789253 3.92011917701607 1.46593552759110

N -3.49986241291559 0.17680137335069 -2.21640204408642

C -7.49349374850741 3.64918244357997 1.24637893107826

C -6.93870382769526 4.91053320481238 1.41325212239386

C -4.70082342726754 -0.15889364461838 -2.94489380895520

C -4.15101614223753 -0.76972477636477 -4.20920864413726

N -2.84267993300523 -0.65445805114706 -4.17451016604425

C -2.27311793442894 -0.06512267695769 -2.97315612117151

C -5.64783810117352 1.02476883245263 -3.23287987919654

C -5.74331945466480 -2.60100676917674 3.42013855196837

C -5.64272329974745 -3.59928622303561 4.37903712698553

C -5.57614120418688 -3.26938153859448 5.72485129927067

C -5.63819674069906 -1.93575491499822 6.10350238216341

C -5.74052582066468 -0.94031859576227 5.14229112008233

C -1.36174796298529 -1.07577064125146 -2.28455899232508

C -1.51218706703910 1.21334964085173 -3.30768282195894

S -5.06117725328025 -1.45539863571504 -5.40803749680660

C -4.94984345650418 2.23554927654164 -3.77788743268870

C -4.55717652419057 3.26292799573884 -2.92218036080005

C -3.86932579927776 4.36763032098330 -3.40425035812649

C -3.56392557448607 4.46255862142626 -4.75547060213390

C -3.96236987922033 3.45150853030544 -5.62019373663902

C -4.65246008074605 2.34898657854337 -5.13464122515728

H -2.20799470071179 1.08606500855941 4.78005463976214

H -3.96289710113794 2.63485249380950 5.42000093415231

H -6.47170551607857 0.63287411477597 3.05736235669828

H -6.24864486983455 -0.55046804528491 1.81027039100353

H -3.00923396791446 -1.89838582200385 4.59701460267701

H -1.33797289091725 -1.38530038802695 4.89529843812418

H -1.68828193377015 -2.45810517429817 3.55053866631235

H -0.20416043489933 0.25201346599427 3.28292027069019

H -0.56362167787545 -0.82898702395110 1.94587591800396

H -1.10054978891740 0.86098249061350 1.87769877468952

H -2.37231219459921 -2.09346058190685 1.42928384876029

H -5.25285526049626 -1.60387676279376 0.44033304243084

H -2.85730042715418 -3.38221639565423 -0.27418007542021

H -6.55487450940845 -2.66270148326081 -0.88289928357721

H -7.80936103422857 -3.73145166027128 -2.68948593720744

H -6.68991642484206 -5.31066120479546 -4.22575979472306

H -4.28606964796686 -5.81997308712473 -3.94576546201874

H -3.00651324183587 -4.74924877912861 -2.13056596146798

H -2.49809791274062 0.98208591266919 -0.61523914267146

H -5.52581283926153 0.51187286410824 -0.41524023067748

H -6.40411086231896 0.65449751832026 -3.92769060186277

H -7.13979571923614 1.56159652576793 1.02534402259886

H -5.11518726470129 6.02271136560409 1.63127790820388

H -3.67601248678993 4.03343013124138 1.55334456352558

H -8.56534938148745 3.53507660605139 1.15124706677707

H -7.57591162363672 5.78410004925068 1.45514121984190

H -5.28482720260023 -0.92855204286709 -2.42514476390665

H -2.24965138784086 -0.99890827133526 -4.91713282388298

H -3.34149287911274 1.86079637750268 1.24594799737014

H -6.17372368154911 1.28575022318111 -2.31491194841596

H -5.82101752926956 -2.88464814919041 2.37875423863956

H -5.62221953098612 -4.63619661740670 4.07021837142810

H -5.49409409556746 -4.04595670480746 6.47388142465541

H -5.61586724346925 -1.66573710149625 7.15112090894453

H -5.81775853906312 0.08939999997898 5.46660360530486

H -1.89438667018426 -2.00575214887173 -2.09121142553941

H -0.99098557339129 -0.68077150627472 -1.33833717564843

H -0.49614537703892 -1.29336255167252 -2.91077326512917

H -0.66098699932732 0.98313198726462 -3.94875008091082

H -2.15305426794111 1.93022825731353 -3.81745035008425

H -1.12349268247102 1.67492861818466 -2.39996334144589

H -4.79185555655568 3.19638051043872 -1.86598998769695

H -3.57318701576672 5.15546850869599 -2.72335634558821

H -3.02677810666341 5.32268349915503 -5.13392548023750

H -3.73917303652625 3.52233738094290 -6.67709954575140

H -4.96538129641439 1.56938434402606 -5.81896240045391

## **11** TS2

94

*E*_El_(SP) = −2644.973644 E_h_

C -0.15014072818430 0.35116198190183 2.46786758728323

N 0.84072629706286 -0.67618646606463 2.20419517702792

C 2.05794376187622 -0.53006079189264 3.02532319923460

N 1.60165907278823 0.51403287917527 3.94451386330718

C 0.44358130468073 1.00484095186333 3.68426224858896

S -0.37068775203554 2.26152779249469 4.48043115949925

C -1.57213539161021 -0.15957131461523 2.75215203093250

C 2.34330741755110 -1.81277447707323 3.78843272086346

C -1.65151359787645 -1.11410356784533 3.91668409958709

C 3.29087812432617 -0.03601520232079 2.28030530616384

C 0.88803053887377 -1.31053944610743 1.02016728420530

C -0.11873628538002 -2.12037181673304 0.40047335721797

C 0.24653008297106 -2.93573828543230 -0.61115048606168

C -0.63913865821211 -3.70460353027365 -1.46324818406984

C -2.02925027721056 -3.73541471445094 -1.29076694730056

C -2.82741514256119 -4.44793857130755 -2.16588056553266

C -2.25805976123257 -5.13708575530829 -3.23261704117778

C -0.88277107325341 -5.11245548229200 -3.41773254868915

C -0.08034014848093 -4.40432793938258 -2.53824652508255

C 1.04932264982083 0.64498283194008 -0.91713023111254

C 0.00579139401626 0.56040931075824 -0.02631167314472

C -0.01637283524397 1.35670445954065 1.22958880146585

C -1.03169083333057 2.47600356280856 1.17761976497089

C -2.35920945603627 2.26899864053500 0.80458292146346

C -1.48477101581402 4.84651870345374 1.33639726111214

C -0.60632198237694 3.77677826014705 1.43500439605138

N 1.04243362372735 0.19969175531770 -2.16630079634914

C -3.23951396918685 3.33625033044788 0.71056404483721

C -2.80608739295756 4.62863001466957 0.97595228732796

C -0.14309197214707 -0.20356882609077 -2.91458993226848

C 0.44649038393375 -0.67063408168157 -4.22103957144929

N 1.73806022242411 -0.42665362301956 -4.19308366067789

C 2.27452232990700 0.14249905084395 -2.96968590053853

C -1.19367453318710 0.90969250866225 -3.09870025481545

C -1.22949843896123 -2.43788183967084 3.80612353939164

C -1.30472787381407 -3.30016438239318 4.89019647076413

C -1.80834161102744 -2.85432660317393 6.10463996362145

C -2.24890646677703 -1.54417966198476 6.22200145260656

C -2.17360669470308 -0.68438947521877 5.13477979987152

C 3.29837121019901 -0.80251445173190 -2.35378732453388

C 2.87604762580385 1.51802043347836 -3.22683581513959

S -0.41493868313400 -1.36822626212837 -5.44453018591952

C -0.62429039697268 2.21750563186134 -3.56259872635307

C -0.33165321124557 3.22262313073935 -2.64315846472657

C 0.24256007011652 4.41749669741786 -3.05431573034076

C 0.52964648585900 4.62581237830988 -4.39644985131084

C 0.22563728770135 3.63775782032575 -5.32406707138629

C -0.34956182268951 2.44438473252941 -4.90991939394605

H 2.20259919034915 0.82916765550542 4.70102498095872

H 0.54515956607436 2.49547123449233 5.43188338509752

H -2.21328443960094 0.70142414536364 2.94657364809990

H -1.97424161846268 -0.61812785891070 1.84903995175899

H 1.47650653206866 -2.12725923391595 4.36624271464960

H 3.18914929669286 -1.66871876106946 4.45982116193262

H 2.60195935421679 -2.60123461298275 3.08219537925066

H 4.07767486224595 0.17454863132138 3.00376129006402

H 3.67292630426913 -0.80355483232306 1.60930276353648

H 3.08915019916573 0.87038058416352 1.71281630333273

H 1.89690503759724 -1.54975733918006 0.69052221021326

H -1.15569255438625 -2.02107140243155 0.68542370514931

H 1.30256897084083 -3.00180271974167 -0.85552188359017

H -2.49450311662635 -3.20245451044567 -0.47173990997920

H -3.89936740127348 -4.46541335611786 -2.02070418893772

H -2.88818977651402 -5.68903764086497 -3.91753291506805

H -0.43560171506399 -5.64308803463984 -4.24758151096479

H 0.99254727191556 -4.38169066965773 -2.68718716926415

H 1.98236055698341 1.08750689490848 -0.58172020580118

H -0.94314502016885 0.16089222838595 -0.36224774561612

H -1.92526622104849 0.51884876264904 -3.80837275035103

H -2.72152275049079 1.27581992762212 0.57045295330892

H -1.13257970933909 5.84960967182810 1.53802076447616

H 0.42604812493327 3.95840235982389 1.70912498470812

H -4.26626219020815 3.15657494453625 0.42019881236669

H -3.49358947726696 5.46032092261563 0.89588643489612

H -0.63963018199630 -1.05632005974137 -2.44620400646391

H 2.34758778540413 -0.65665127624357 -4.96632392091974

H 0.96599291113060 1.82407919493399 1.34465316018281

H -1.72510008927913 1.04803719792633 -2.15743632808024

H -0.84533456110434 -2.81568346877801 2.86785710401487

H -0.97232210854186 -4.32435966596307 4.78220960473087

H -1.86601163044453 -3.52710058466794 6.95028338037099

H -2.65861457437684 -1.18898098130983 7.15851376219673

H -2.54011173796432 0.32965940567568 5.23508294398061

H 2.86533076441328 -1.78618696010502 -2.17842649301297

H 3.66968153382107 -0.40673887722380 -1.40816499448914

H 4.15126815670184 -0.90914298882071 -3.02368351351134

H 3.72569032893029 1.42280037543168 -3.90295912736636

H 2.14706891062819 2.19250859758996 -3.67132478130545

H 3.24339849761071 1.95720459708866 -2.29966161686453

H -0.56035757361763 3.07561538362140 -1.59344364848288

H 0.46377691335323 5.18601349862888 -2.32473063727994

H 0.97865107822184 5.55606599210204 -4.71968311295044

H 0.43307971455360 3.79781758369714 -6.37431835138115

H -0.59144529234356 1.68300875419468 -5.64203444306476

**11-Im4t-adduct**

94

*E*_El_(SP) = −2645.011248 E_h_

C -4.24972495849750 0.50126149060106 2.58510215238657

N -3.65831433891095 -0.78128776436719 2.25259270580882

C -2.63098372398269 -1.18263888893909 3.21406308877765

N -2.65653987882452 -0.02576514611189 4.13391927080833

C -3.49045981740699 0.89417541245496 3.81704296029502

S -3.76274693634994 2.39237730956098 4.57672871830140

C -5.76804085321546 0.41170131463980 2.85756354937147

C -3.01490691824756 -2.44339269523817 3.97515528921769

C -6.13387495350440 -0.64834317146359 3.85717270202119

C -1.21895716654048 -1.30668331025759 2.65458227848874

C -3.43606945347944 -0.91874584516673 0.82478423531467

C -4.12267780544525 -2.09275265684689 0.20927493597882

C -3.51362768467017 -2.91110299482193 -0.64922461864586

C -4.13608271067997 -4.00012460456460 -1.40434303163007

C -5.41351101103175 -4.49025383916397 -1.11797061674782

C -5.97069529518288 -5.49695552968109 -1.88785428951377

C -5.26553852948863 -6.03573872582139 -2.95887966889750

C -3.99386565063526 -5.56316656559627 -3.24922311849471

C -3.43418906456228 -4.55758864483015 -2.47474010259922

C -3.13348069588436 0.72192676745545 -0.92710661735292

C -4.00064463845284 0.41613999258010 0.22616587907055

C -3.86053082437449 1.41450523514648 1.37849626152917

C -4.55373853012465 2.73848023714900 1.20494682656954

C -5.90152405286652 2.85687861647449 0.86535054735679

C -4.38188435628232 5.15289795146002 1.19060292318282

C -3.80406530960899 3.90284805503475 1.36028308850066

N -3.38376817592057 0.45617460648635 -2.15075105665139

C -6.48355342068205 4.10512281289281 0.70467413282414

C -5.72632569416595 5.25742315370493 0.86571915329239

C -4.65415362544401 -0.07350512392851 -2.68423272917148

C -4.25367325634814 -0.55531915690004 -4.05299328830335

N -3.02528070244305 -0.14182642823004 -4.28804528818626

C -2.31955821127470 0.51887455127089 -3.20844894571160

C -5.78964376856932 0.96336571700348 -2.72993942590258

C -6.50350864184348 -1.91751763270024 3.41888158820603

C -6.81837721614082 -2.92067181314525 4.32348589157901

C -6.76350470693403 -2.66878616595572 5.68780284602788

C -6.41301292530270 -1.40307461811642 6.13679167038107

C -6.10803911378901 -0.39895573823833 5.22732036426577

C -1.10997348587188 -0.30871976284274 -2.79868220167186

C -1.93802055416553 1.94575708281667 -3.55843531169330

S -5.23229325476893 -1.43248876207341 -5.04055898703781

C -5.44103611854880 2.25187648322101 -3.41812932759574

C -5.03739832207468 3.35710419841769 -2.67258637744681

C -4.68672647008587 4.54634341289702 -3.29609378917772

C -4.73233555848864 4.64416738946869 -4.67962884731832

C -5.14432436347384 3.55167068276258 -5.43225490514883

C -5.50160148744790 2.36601097506044 -4.80597801808949

H -2.03116256726669 0.01988324822030 4.93355202922373

H -2.88508492975861 2.25183633473773 5.58243817214215

H -6.14742836681267 1.38702259451835 3.16942647402020

H -6.25422602775019 0.17535467745165 1.90986027081028

H -3.98296960858372 -2.33575858501535 4.45897554712814

H -2.26299687055572 -2.68615340371280 4.72608382693905

H -3.07331921960886 -3.27243228887101 3.27046467793883

H -0.52201179963405 -1.48304847953988 3.47369228567486

H -1.14612216216545 -2.16162853632608 1.98306493722965

H -0.90505874778520 -0.40678054863295 2.12553621131345

H -2.36601936222815 -0.98183357045638 0.59624908881294

H -5.17812153301989 -2.19383030312336 0.44169158760216

H -2.45836091930279 -2.74770659908914 -0.85404753248071

H -5.97787830000665 -4.09300184128181 -0.28344872465683

H -6.95966570807484 -5.86713441396062 -1.65073404077043

H -5.70490102244418 -6.82189774806774 -3.55882486830634

H -3.43504050853406 -5.97733989707610 -4.07824370886952

H -2.44059083582595 -4.19173792878791 -2.70609562049314

H -2.14576305063556 1.11644015656334 -0.70267766937937

H -5.04392344180950 0.27967670666362 -0.05799426744993

H -6.61829967898813 0.45775637972652 -3.22882025129149

H -6.51341537158555 1.97955794768964 0.70350222666974

H -3.77934752110867 6.04332524567895 1.31228298423456

H -2.75337610128682 3.83137332209874 1.61437198327873

H -7.53027009552349 4.17455081911349 0.43974121977794

H -6.18124664618218 6.23014904534965 0.73281597711167

H -4.96835310759875 -0.92835318498089 -2.08387794428980

H -2.54437175586457 -0.34767769859311 -5.15455473171584

H -2.78900944101580 1.61689481851745 1.49616142734959

H -6.12261907387530 1.16082537892366 -1.71109515218669

H -6.54813789987734 -2.12331697983788 2.35672856851098

H -7.10457451542297 -3.90014314782256 3.96255365285567

H -7.00420964516819 -3.45063806927358 6.39633979247286

H -6.38526448321365 -1.19140175581587 7.19783344406102

H -5.86448106684218 0.59013469803821 5.59657757245616

H -1.40366055217277 -1.31310764227108 -2.49758982586971

H -0.55364595565509 0.16495842095615 -1.99081669449240

H -0.43814502113944 -0.38608132644491 -3.65305445013647

H -1.18583279528565 1.91813756497300 -4.34597487014027

H -2.78919050178549 2.52005398407666 -3.91365804831376

H -1.49996366600013 2.44803482964510 -2.69665244501716

H -5.00024975963250 3.29037155390605 -1.59238766974470

H -4.37764888017795 5.39427096377932 -2.69863035290957

H -4.45723125298130 5.56890499573202 -5.16991390690119

H -5.19462337671996 3.62448637807776 -6.51090383103786

H -5.83472759096527 1.52532796306550 -5.40234982656192

***ent*-11** TS1

94

*E*_El_(SP) = −2644.957856 E_h_

C -8.26311758483556 1.87657052929619 -1.77880956936068

N -7.46100317432758 0.75571639208269 -1.43716641080875

C -6.00342338903608 1.08598000170159 -1.45829493096825

N -6.08484195009768 2.49170757135155 -1.81593497480394

C -7.30196847938095 2.88515995421371 -2.08492014559142

S -7.73627225720766 4.41460854174316 -2.72394611330663

C -9.44996690844209 1.69425287732191 -2.69980054295851

C -5.28759353849144 0.30552476015114 -2.55071785590614

C -9.15679686062250 0.83724384391696 -3.91013357524187

C -5.34447006947548 0.91164899071963 -0.10257757789419

C -7.83661353434340 -0.46051214584353 -1.15862991948349

C -9.14508429571173 -0.98746564137097 -1.06985767412122

C -9.27977504726579 -2.32257002927518 -0.88010555944354

C -10.51988989776130 -3.04547815304462 -0.72410757227628

C -11.77665853515131 -2.45210991630464 -0.90461655905095

C -12.92921369453545 -3.18900125507850 -0.71066975143957

C -12.85008667746445 -4.52611769442686 -0.33112256940149

C -11.61129521218897 -5.12664915142880 -0.15002743809846

C -10.45478377492432 -4.39263560809573 -0.34954682186987

C -8.28810706169861 1.30862899269446 2.02081563245880

C -9.21113620262329 1.52295118865557 1.02210256783612

C -9.03673886817871 2.62207039988730 0.14965842483598

C -10.19814280823879 3.40042686110105 -0.29692027479193

C -10.06197275303994 4.78220180310127 -0.45355786731509

C -12.52887619186816 3.60122467108465 -0.90345510021079

C -11.45534811406000 2.82316887035529 -0.50891606283338

N -8.40240845419361 0.50348781275473 3.06900647533957

C -11.14167118629253 5.56198433636852 -0.83848950584022

C -12.37478571231370 4.97240820575380 -1.07514323835349

C -9.59575549743037 -0.26914732837034 3.38698190523887

C -9.34930844042096 -0.69057152984005 4.81188128128920

N -8.15435304672306 -0.26042645940580 5.15605845288319

C -7.47673235374390 0.61139058904754 4.21330438454367

C -9.86398816669415 -1.47521075170829 2.47590610400164

C -9.91763419813100 -0.30112250770321 -4.15731259588172

C -9.67123827380843 -1.09505438766077 -5.27008784335726

C -8.65679921282865 -0.75866183279981 -6.15369447067919

C -7.89875253253432 0.38240428581710 -5.92327131794645

C -8.14975107614778 1.17407758172306 -4.81266880554985

C -7.43002626807446 2.03050362218281 4.77375541055736

C -6.08304290214424 0.09986154011310 3.88655513545730

S -10.41024094253204 -1.55110451346483 5.73504412701616

C -8.83667321978578 -2.56917604329700 2.52022484876346

C -9.14026002193520 -3.79005639333069 3.11896066018962

C -8.21821584589621 -4.82732068671071 3.13482221535355

C -6.97005500369415 -4.65928779729536 2.55063801305754

C -6.65361914978236 -3.44735981118636 1.95234570908156

C -7.58106808518714 -2.41460708648196 1.93684918957381

H -5.25158566633915 3.05800994642233 -1.90855325803791

H -6.49346408502385 4.89929436764954 -2.84496396586126

H -10.30900999283656 1.28989568600088 -2.17115392927693

H -9.77537911135741 2.68816338671421 -3.01660560351576

H -4.27618211308651 0.69472717642927 -2.66253974701540

H -5.20835171402708 -0.74735683151662 -2.28236035299914

H -5.80748371701067 0.39331591766241 -3.50342297655649

H -5.80164762195973 1.55272664721383 0.64526828808208

H -5.39778283373629 -0.12493437521789 0.22844217703663

H -4.29148342778997 1.17844931774087 -0.18507798768736

H -7.02451807899777 -1.16172113103000 -1.00156312413405

H -10.00881150959560 -0.34286103777870 -1.10496244242311

H -8.37793850925527 -2.92390253210066 -0.81667004630167

H -11.85884758230749 -1.41485371636058 -1.20415108274796

H -13.89504085348794 -2.72413751746219 -0.85623373779573

H -13.75616036081010 -5.09785790336929 -0.17939142169710

H -11.54790328916787 -6.16505933918660 0.14594744404962

H -9.48736469547377 -4.85747291307279 -0.20246811466817

H -7.38103583194467 1.90196302654313 2.00746975553726

H -10.15324108431382 0.99234413866652 1.02505738852566

H -8.14902425628538 3.22063641431300 0.33456532129634

H -9.10976568871901 5.25298247319284 -0.24799000694614

H -13.49194619421261 3.13795629802548 -1.07148493924220

H -11.60516060176651 1.75942631391839 -0.37092456991957

H -11.01871642696390 6.63122323512229 -0.94812752580876

H -13.21762267706385 5.57854158411185 -1.38005940393715

H -10.47752473586982 0.38071342200781 3.35329513904932

H -7.77272840450810 -0.41118497389559 6.08039950296337

H -10.83700212717391 -1.86808831587094 2.77298244600842

H -9.98291975219208 -1.11192774058562 1.45561075645607

H -10.71734060201868 -0.57287777465854 -3.47844431696479

H -10.27383688848139 -1.97727970671745 -5.44247198647731

H -8.46004887405421 -1.37725679678865 -7.01949154084635

H -7.11239201873762 0.66101377181940 -6.61260648525086

H -7.55500265957255 2.06774273134704 -4.66653263806455

H -8.43409270950106 2.40815467419204 4.96512095446360

H -6.92099362443200 2.70875033742379 4.08941687720062

H -6.87142484052279 2.03206281273799 5.70957588077745

H -5.45890290273070 0.14531968572346 4.77882825716193

H -6.11459623744727 -0.92671089640509 3.52963234989094

H -5.61565301166566 0.72566411868072 3.12636363852701

H -10.11347144476887 -3.93115126608184 3.57227650578153

H -8.47759552946506 -5.76898182648013 3.60120711405945

H -6.25072009731977 -5.46786787236979 2.55860199559149

H -5.68497125873729 -3.30478966498180 1.49042026185946

H -7.31741690120027 -1.48088986725311 1.45651824739866

***ent*-11** IM

94

*E*_El_(SP) = −2644.982611 E_h_

C -8.42312962780704 1.90239575953050 -1.72043943248199

N -7.43422892388947 0.81173442044085 -1.64276706969891

C -6.05415487819877 1.25731390878164 -1.96584303339257

N -6.35097676121547 2.58992462997611 -2.47661012524104

C -7.58300385488054 2.95047878558909 -2.41233273398610

S -8.25599245666039 4.40737444890421 -2.96857063279039

C -9.67536337466836 1.54679894571628 -2.55010255542817

C -5.41969968902614 0.42535791501757 -3.06976999828450

C -9.42405363474963 0.72830483965755 -3.79360782340075

C -5.17025431060513 1.34319415341888 -0.73169306507644

C -7.62803013209883 -0.44175867783855 -1.34017874683699

C -8.81409245334264 -1.14502109243870 -1.07269482448768

C -8.70403683572475 -2.49439062487550 -0.92766492558596

C -9.77196908826873 -3.43458670578767 -0.71943459588352

C -11.12417768688824 -3.06392389919352 -0.69557887694471

C -12.10022093175294 -4.01237754894753 -0.46308346920560

C -11.74667657495258 -5.34318224071085 -0.25615214071723

C -10.41211880167227 -5.72631326160471 -0.28766129983900

C -9.43183762988155 -4.77862074115766 -0.51934320095722

C -8.38485977621669 1.17480167096419 1.81534009010417

C -9.15314079780678 1.41845162035088 0.73725323553988

C -8.71826519753677 2.45439109932899 -0.25148453061069

C -9.67015564194697 3.63530245378042 -0.22499502817649

C -9.17066156130063 4.89258948183265 0.10740081366251

C -11.87979194062101 4.60442263702480 -0.36141634562509

C -11.04133852953403 3.50189070442138 -0.43802826274856

N -8.67465187073386 0.41643209785107 2.90595055071357

C -10.00778476506294 5.99482573774891 0.19508656176225

C -11.36634789763525 5.85581338600775 -0.04904794601403

C -9.97790973545263 -0.17580525570362 3.11358825854613

C -9.98853205047967 -0.46408016574342 4.59160292217812

N -8.83625629752941 -0.06396504438754 5.08154261883425

C -7.95823064499349 0.64651146751645 4.16514993057325

C -10.23968102146370 -1.43409434761223 2.27683592440443

C -10.03337444170628 -0.51611003598520 -3.93403347486407

C -9.82863479401504 -1.29100449799833 -5.06718822774498

C -9.00938051203783 -0.83020765793859 -6.08654061541974

C -8.41789551158389 0.42031420505031 -5.97261919163576

C -8.63001620785429 1.19423858453987 -4.84097721991008

C -7.91155702933404 2.12311448631718 4.55597670781201

C -6.56488892545444 0.03590501372201 4.16013719776178

S -11.24155072077590 -1.15490690701261 5.41881583221356

C -9.33221134492735 -2.59425807907953 2.56668974216602

C -9.82481863068443 -3.72367993942870 3.21606000869426

C -9.00929113932822 -4.81961112453683 3.46429425561924

C -7.67925536194949 -4.80235737212573 3.06692019656393

C -7.17504531704474 -3.68216234281267 2.42002088642093

C -7.99495028944666 -2.59004829372411 2.17139771263633

H -5.60382937664085 3.16349245751268 -2.85771929968339

H -7.13697195545772 4.92462255357066 -3.49986734093309

H -10.39742910886901 1.03839239677607 -1.91296402011516

H -10.16485096399301 2.48556752205229 -2.81938573540902

H -4.49846885540337 0.91054562396548 -3.38982052217540

H -5.15151108799822 -0.56406761163906 -2.70397284259220

H -6.08515717514933 0.32580395867697 -3.92566977138068

H -5.60842288620594 1.97639017526575 0.03731637519735

H -5.00825040860425 0.34726615043493 -0.32147163735854

H -4.20037688743864 1.75266739053575 -1.01105109047244

H -6.72207779491758 -1.03743244021861 -1.33935084330634

H -9.76535521420968 -0.64574826399104 -1.00906660692498

H -7.70806118610779 -2.92412614415419 -0.97348504363739

H -11.42029154980869 -2.03587407789057 -0.85969523954329

H -13.14117823966754 -3.71956341372514 -0.44446994022499

H -12.51617638698876 -6.08154783741327 -0.07295640481784

H -10.13861363882312 -6.76047376667064 -0.12892373312573

H -8.38979470683920 -5.07325326830208 -0.53619733409417

H -7.40290179736793 1.63612674976606 1.85420393172332

H -10.15853132199992 1.02388420095814 0.64738987393591

H -7.75267463291925 2.83584887417128 0.09179067255999

H -8.11039134735426 5.01455844370948 0.29558544461087

H -12.94028700300144 4.48072190005219 -0.53757043484776

H -11.47856348443630 2.53533291040507 -0.65168765027291

H -9.59587942304672 6.96184608792336 0.45240887881387

H -12.02292824807677 6.71369373391691 0.01323920753214

H -10.77719290115711 0.54680286402787 2.89624929586989

H -8.61739442229937 -0.12603265999281 6.06648857845279

H -11.27859562835746 -1.72093760800189 2.44379913729346

H -10.16552147922898 -1.15437582853198 1.22601813325546

H -10.69157354571445 -0.88536231620050 -3.15761914188468

H -10.31365364710859 -2.25472429768804 -5.14981524596534

H -8.84274647336623 -1.43371628609866 -6.96903340127671

H -7.79524756058526 0.80317291747202 -6.77044634378307

H -8.18498919095618 2.18015733004519 -4.80988194213431

H -8.90825317225576 2.56372485280505 4.52834265615701

H -7.26095372070838 2.68545755340841 3.88665783931397

H -7.51343861245380 2.23000936195499 5.56540391036715

H -6.08229033051144 0.18769451122208 5.12585441701520

H -6.60970782674823 -1.03107691176741 3.95126773346174

H -5.94381391378308 0.51482649894322 3.40256974025387

H -10.86174462007624 -3.74564236843553 3.52725142353691

H -9.41543210009934 -5.68866290977675 3.96593224096434

H -7.04161286572688 -5.65609876438723 3.25695465197084

H -6.14017198403428 -3.65782058763840 2.10282587509150

H -7.58616776237341 -1.72960381417917 1.65911724982783

***ent*-11** TS2

94

*E*_El_(SP) = −2644.965187 E_h_

C 0.35940511923803 1.83252599847825 -1.82799164390136

N 1.21313831729478 0.68340134892170 -1.62971027985678

C 2.63698654873694 0.95198639988772 -1.89743971643830

N 2.51797403223510 2.29269902935266 -2.47117007298396

C 1.33586752783537 2.79462136260804 -2.44959706094464

S 0.84349264564349 4.33402971383286 -2.96577139363490

C -0.84997984297553 1.61795635045738 -2.75830729207136

C 3.17432250446718 -0.01922400034699 -2.93559427407720

C -0.48356093674889 1.10655903373922 -4.12790479478339

C 3.53660984138745 1.01173903335012 -0.66925087978515

C 0.83992579113580 -0.32036828048807 -0.82512227857994

C -0.33752122355725 -1.13744294481563 -0.95491103139277

C -0.26107074533195 -2.42824243541911 -0.57481760720399

C -1.32508879783567 -3.41458132597101 -0.60789184895246

C -2.59377566450019 -3.15557699515429 -1.13931128390183

C -3.57445420652134 -4.13002497233026 -1.11264738473925

C -3.30932960108638 -5.37695636058719 -0.55429662385140

C -2.05497923617777 -5.64647668900843 -0.02462257952346

C -1.06998177862613 -4.67307590292735 -0.05449056412053

C 0.58905644186851 1.11918562398922 1.72509333278766

C -0.19584202668312 1.17902426086174 0.58972147623674

C 0.01262541808023 2.33791736943844 -0.34126846293398

C -1.11981423604527 3.33789727907399 -0.27086882004485

C -0.82911938788107 4.68233637470180 -0.05586167817336

C -3.47110252765850 3.89508746546765 -0.29186295660363

C -2.45678658469071 2.95443879571607 -0.37619021343042

N 0.32570067321378 0.51673524386580 2.87672031593081

C -1.84379124846366 5.62610777095704 0.03181215905544

C -3.16846366716119 5.23587748415005 -0.09061267958956

C -0.97560935166569 -0.03522107697202 3.23671413827154

C -0.87276023624279 -0.18311562974183 4.73225932962852

N 0.34352584861544 0.17166380512948 5.08839772657843

C 1.17790628290727 0.76429953634932 4.05992591777426

C -1.35483421587404 -1.36167945315844 2.56521605423872

C -0.15875998315633 -0.23293398184919 -4.33507381518181

C 0.17423356848836 -0.69546833401498 -5.59967683863851

C 0.18492906484163 0.17459220886436 -6.68159582886024

C -0.15371409980056 1.50626002645511 -6.49121110482494

C -0.48847681944183 1.96555943107154 -5.22452092405523

C 1.33974033604596 2.25797017927721 4.33597751049290

C 2.52913404407847 0.07427731765042 3.96830363955340

S -2.08788896452847 -0.71510469638989 5.71040790326975

C -0.51233342614706 -2.54975543196315 2.93484571652105

C -1.06909755175376 -3.60143659788042 3.65895013653537

C -0.32069436166870 -4.72656508869118 3.97659043777345

C 1.00619611495740 -4.81631516589649 3.57865141279924

C 1.57470030324335 -3.77379348067794 2.85979599157235

C 0.81954677779791 -2.65385242904560 2.53979960916642

H 3.33989766325662 2.77370338075030 -2.82563215059197

H 2.03821015355675 4.76998718443937 -3.39095325197975

H -1.55935366674010 0.94517601243251 -2.28085797810540

H -1.37786535658142 2.56858197069332 -2.84644541253932

H 4.19834609968617 0.24328573548053 -3.19871960935347

H 3.18008608295879 -1.02603244903162 -2.51860531195975

H 2.55916686215985 -0.01375444090962 -3.83342502014643

H 3.18275556070586 1.72890217242656 0.06866639525467

H 3.62410349748749 0.03168493558799 -0.20340971833688

H 4.53726420172153 1.30833145531274 -0.98169195170451

H 1.67112742289026 -0.83372615947170 -0.34906553957640

H -1.25766404758982 -0.72519962846622 -1.34142996535477

H 0.68732613329173 -2.78826763524375 -0.18960226523627

H -2.82041377560945 -2.19293480882798 -1.57914223542803

H -4.55106890935165 -3.91999123854265 -1.52800927178619

H -4.08116718204425 -6.13507088058926 -0.53392182453846

H -1.84444036680423 -6.61344100012082 0.41239962630039

H -0.09329548660882 -4.88175530816767 0.36566105226257

H 1.54033734159377 1.63945866160370 1.70354542769120

H -1.19032621712913 0.75116915870878 0.62318969603211

H 0.91582873801460 2.85955556559664 -0.01130955922138

H 0.20115297185088 4.99906925488738 0.05152398750469

H -4.50188746607445 3.57728722002652 -0.37797926969403

H -2.72380236914591 1.91456605211969 -0.51781615995909

H -1.59482617367384 6.66560199376572 0.20021618346932

H -3.96187919805016 5.96845448502304 -0.02198626794771

H -1.75698171861332 0.69668607173699 3.00152521334291

H 0.64324511446342 0.17114331401716 6.05423691871226

H -2.39413424755102 -1.54759705747191 2.83798935326701

H -1.34695168460616 -1.21449322039074 1.48536075957862

H -0.17289779162308 -0.93358307138795 -3.51023912936057

H 0.42075060346832 -1.73999589450865 -5.73927124214729

H 0.44474974508318 -0.18618410819358 -7.66822262223309

H -0.16552466901615 2.19037947581479 -7.32960976884832

H -0.77187900328822 3.00251894164026 -5.09480213042983

H 0.37350190883195 2.76232453323412 4.34368213877103

H 1.98184449423456 2.73278307389348 3.59459035666161

H 1.81056222413191 2.39733444759912 5.30904391942187

H 3.10257554693050 0.26890624301910 4.87453261221548

H 2.41990824913916 -1.00105639513910 3.84955454498506

H 3.09919252356023 0.46844511672277 3.12673406008442

H -2.10391062028689 -3.54139923101600 3.97256482607388

H -0.77638831610309 -5.53394024791851 4.53518722077764

H 1.59187476892096 -5.69262920775339 3.82433451589364

H 2.60744391284346 -3.83246020730890 2.54060325411988

H 1.27921996582069 -1.85936143639023 1.96741471895179

***ent*-11-Im4t-adduct**

94

*E*_El_(SP) = −2645.010659 E_h_

C -8.32778773035350 1.94720735520819 -1.81828863676798

N -7.80011213014851 0.61080055025405 -2.01702130318211

C -6.60188749039972 0.61070500115780 -2.85506032345956

N -6.43449264859798 2.06167464552248 -3.09067749517044

C -7.31932617312294 2.80112930615296 -2.53178766474211

S -7.43739244347429 4.49895710228467 -2.51692040218933

C -9.74279674252070 2.14891760407578 -2.40845342509505

C -6.82093550916088 -0.09344100984626 -4.18665640285417

C -9.87650587219890 1.68651474452011 -3.83038245101289

C -5.33118188716443 0.09543986908012 -2.18961919403769

C -7.83780117966357 -0.17487455503337 -0.78606389957468

C -8.45247852964873 -1.51727171607235 -1.00657220432373

C -7.80722869810848 -2.65301359669232 -0.73910883314717

C -8.32611931844420 -4.01473712061409 -0.88367821534695

C -9.60842768802263 -4.29383932847254 -1.36580259247891

C -10.05755586451376 -5.59968308791563 -1.46180220344074

C -9.23806606985656 -6.65583021355108 -1.07723302662483

C -7.96344070405832 -6.39335922059467 -0.59698859526904

C -7.51344760985044 -5.08414659208145 -0.50274954021745

C -8.17397360826683 0.59502922279524 1.58120036978321

C -8.64340735209082 0.71663541851277 0.18714479080257

C -8.26303682388072 2.14534607423238 -0.27489174802134

C -9.01245171077178 3.28056700878739 0.36714650769740

C -8.27425681735420 4.33712589041590 0.89757058108296

C -11.03071787990462 4.37612999541130 1.12266715997826

C -10.40193486731129 3.31363170541742 0.49071859344701

N -8.88635238102582 0.55992860644089 2.63882741462273

C -8.90145037522116 5.40309073174926 1.52592718429585

C -10.28353263984596 5.42541115169246 1.64010089411153

C -10.35880547578024 0.50535742260453 2.71186816037708

C -10.62600580252461 0.86033161719564 4.15062348273817

N -9.47318447382748 0.87719504907508 4.78704562590689

C -8.26909199880476 0.72374117301850 3.99731230222741

C -10.95235543687172 -0.85793603483811 2.31811840191545

C -10.32925224724305 0.39682706104282 -4.09802001061034

C -10.44320134256006 -0.06125676994635 -5.40215274505865

C -10.09922396142652 0.76658446410709 -6.46269528377508

C -9.65829615154678 2.05798944323855 -6.20878794920739

C -9.55450102130563 2.51603415507842 -4.90181869770805

C -7.44828498745935 2.00538946852439 4.05001476513519

C -7.46234766244293 -0.49512319291831 4.40565863437953

S -12.12463665387556 1.14882919217824 4.76048251320461

C -10.38689380466304 -2.03102132551899 3.06846991272681

C -10.81611097339509 -2.34389918403639 4.35778176992680

C -10.25298537604344 -3.40668440011705 5.04971597653467

C -9.26177718371746 -4.18055888741919 4.45944747463881

C -8.84359919869013 -3.89044680012259 3.16859967326063

C -9.40380819705707 -2.82237895572380 2.48176865426881

H -5.65958846480800 2.41464966200194 -3.64525988139148

H -6.35614183265686 4.77087020719012 -3.26303161356208

H -10.44073017868471 1.57895389836150 -1.79435806214875

H -10.03363008680336 3.19767064408048 -2.31288616637879

H -5.93532284150313 -0.01272157802293 -4.81723570622964

H -7.00896854131447 -1.14988715592497 -3.99765651845404

H -7.67496625230865 0.31776709437018 -4.71904454396629

H -5.11012066861521 0.61329093546465 -1.25649786191288

H -5.40801983256433 -0.97378728419931 -1.99635791942893

H -4.49033939528744 0.24181548679378 -2.86716236693044

H -6.83427297296746 -0.30978700796946 -0.36762457615539

H -9.46394542465138 -1.51194656016940 -1.40135874553663

H -6.79411870598785 -2.58494444140949 -0.34998434756234

H -10.26650528436602 -3.48954225227829 -1.66947209871241

H -11.05329114793227 -5.79693827602991 -1.83721990801187

H -9.59354199523768 -7.67516053366800 -1.15202316186666

H -7.31736200408477 -7.20707207411084 -0.29429696821026

H -6.51731661221538 -4.88605938595800 -0.12482103017407

H -7.09709990959671 0.63301229513872 1.72368796746604

H -9.71405175029445 0.54191364336675 0.08181523370032

H -7.20185839472669 2.28007360882468 -0.03242349860273

H -7.19349973457179 4.32237773297496 0.82444264381228

H -12.10889636267875 4.37957867724905 1.21179261652099

H -11.01725258462225 2.51260185949305 0.10053149579039

H -8.30762905480863 6.21180346719555 1.93088345807516

H -10.77661583792659 6.25214918912828 2.13419961217223

H -10.77644079249023 1.28254960955720 2.07168652636165

H -9.40634136009058 1.09652721658185 5.77290503082521

H -12.02642055514603 -0.76092248901135 2.48586415429215

H -10.82061936923458 -0.99589305639556 1.24405630429116

H -10.59678447579820 -0.25665599270583 -3.27667570644965

H -10.79827581928830 -1.06627639918119 -5.59023800827377

H -10.18394279773830 0.41095190634893 -7.48132526623067

H -9.40245592746498 2.71573368863331 -7.02932474026138

H -9.23232649451745 3.53519749606367 -4.72337723910184

H -8.02320429037075 2.85357375400404 3.67947029003610

H -6.52664070427809 1.91671457037003 3.47608465598520

H -7.16966590252488 2.19682672410287 5.08576154714323

H -7.05879714007362 -0.32632133713798 5.40339295897838

H -8.07109039874569 -1.39560124320055 4.42420398195529

H -6.62349407020903 -0.64115068981327 3.72666433037679

H -11.59836596223805 -1.75743243330587 4.82358135811073

H -10.59481395651361 -3.63468649107522 6.05098861104228

H -8.82599168406721 -5.01048460502076 5.00034744203916

H -8.08030264716600 -4.49417907168656 2.69436960402792

H -9.07013810181774 -2.59870106379068 1.47710510303751
